# GDF-8 improves *in vitro* implantation and cryo-tolerance by stimulating the ALK5-SMAD2/3 signaling in bovine IVF embryo development

**DOI:** 10.3389/fcell.2024.1345669

**Published:** 2024-03-21

**Authors:** Seon-Min Kang, Muhammad Idrees, Chalani Dilshani Perera, Seo-Hyun Lee, Mingjun Zhang, Xianfeng Yu, Yongxun Jin, Il-Keun Kong

**Affiliations:** ^1^ Division of Applied Life Science (BK21 Four), Graduate School of Applied Life Science, Gyeongsang National University, Jinju, Republic of Korea; ^2^ Division of Animal Science, Institute of Agriculture and Life Science, Gyeongsang National University, Jinju, Republic of Korea; ^3^ Jilin Provincial Key Laboratory of Animal Model, College of Animal Science, Jilin University, Changchun, China

**Keywords:** GDF-8, ALK5-SMAD2/3 signaling, embryo development, *in vitro* implantation, cryotolerance, bovine

## Abstract

Transforming growth factor-beta (TGF-β) plays a critical role in regulating trophoblast invasion and proliferation. Growth differentiation factor-8 (GDF-8) is a member of the TGF-β superfamily and is categorized as a myostatin subtype. It is primarily a secreted protein synthesized in skeletal muscle cells. It is expressed in the placenta, reproductive tissues, and cells. In this study, we investigated the role of GDF-8 in the development and hatching rate of bovine embryos. We noted a notable elevation (*p <* 0.05) in the development and hatching rates compared to the control embryos. Furthermore, the GDF-8 group showed a significantly improved total cell number (*p <* 0.05) and an increase in trophectoderm ratio inner cell mass (trophectoderm: inner cell mass) cells (*p <* 0.001) compared to the control group. Additionally, blastocysts treated with GDF-8 exhibited significantly higher mRNA levels of caudal-type homeobox 2 (*CDX2*) (*p <* 0.05). The trophoblast invasion area was significantly larger in the GDF-8 group than in the control group (*p <* 0.01). Furthermore, qRT-PCR analysis revealed significantly higher mRNA levels (*p <* 0.05) of matrix metalloproteinases 9 (*MMP9*) and follistatin-like 3(*FSTL3*), both of which are associated with the ALK5-SMAD2/3 signaling pathway, in the GDF-8 group than those in the control group. The mRNA expression levels of genes related to tight junctions (TJ) and adherent junctions were higher in the GDF-8 group than those in the control group (*p <* 0.05). After 24 h of thawing, blastocysts were analyzed using 4-kDa FITC-dextran, which revealed a higher TJ integrity in the GDF-8 group (*p <* 0.01). Thus, GDF-8 plays a crucial role in bovine embryonic development, *in vitro* implantation, and cryotolerance.

## 1 Introduction

Embryo transfer and *in vitro* embryo production (IVP) represent indispensable assisted reproductive technologies in the commercial cattle industry for high-capacity cattle and serve as vital tools for controlling gene expression in blastocysts. Assisted reproductive technology has undergone significant advancements, particularly cryopreservation, which is unhindered by spatial and temporal constraints (Thibier, 2005). Nevertheless, the disparities between *in vitro* and *in vivo* embryo culture conditions within the female reproductive tract subject embryos to diverse physical and chemical stimuli, ultimately resulting in limitations regarding their developmental rate and quality (King et al., 1988; Underhill et al., 1991).

Despite extensive efforts to mitigate these differences, disparities persist between developing embryos cultured *in vivo* and those cultured *in vitro*. These disparities include morphological and molecular factors compromising IVP effectiveness (D. Lechniak et al., 1997; Thompson, 1997; Peter Holm, 1998; Khurana and Niemann, 2000; Corcoran et al., 2006; Camargo et al., 2018). Due to its inability to faithfully replicate *in vivo* culture conditions, IVP technology exhibits inherent developmental rate and quality differences. Consequently, the development rate of *in vitro* cultured bovine embryos remains limited to a 20%–30% range (Thompson, 1997; Camargo et al., 2006). Moreover, *in vitro* cultured embryos display a slower cell cycle division rate than *in vivo* cultured embryos, resulting in negative effects (Thompson, 1997). Furthermore, when embryos undergo cryopreservation, they are subjected to significant damage in their morphology and function. The extent of damage varies depending on membrane permeability, shape, and cell size (Marsico et al., 2019). Subsequently, the viability and quality of cryopreserved embryos depend on their cellular competence, which affects their ability to survive cryopreservation, potential for successful *in vitro* implantation, and preservation of structural integrity during *in vitro* culture (Thibier, 2005; Moussa et al., 2014). During the thawing process following cryopreservation, the trophectoderm (TE) cells of bovine blastocysts are susceptible to damage due to cryo-shrinkage, resulting in detrimental effects on their re-expansion capability (Kaidi et al., 2001; Camargo et al., 2011). Recent studies have emphasized the significant contribution of TE cells to early embryonic development and uterine implantation (Gauster et al., 2022). These cells undergo differentiation as part of the embryonic developmental process, controlling the movement of small molecules and H_2_O, which are crucial for forming of the blastocoel cavity. Consequently, if these cells experience damage to their intercellular junctions due to cryo-shrinkage, they cannot maintain pressure within the blastocoel cavity (Choi et al., 2012; Marikawa and Alarcon, 2012). Moreover, previous research has indicated that TE cells are more sensitive to cryopreservation and exhibit a more pronounced response to thawing than inner cell mass (ICM) cells (Kaidi et al., 2001). The preservation of the epithelial integrity of TE cells requires specific conditions.

Transforming growth factor-beta (TGF-β) is also critical in regulating trophoblast invasion and proliferation (Adu-Gyamfi et al., 2020). Additionally, *in vitro* porcine embryo systems enhance blastocyst formation rates during early embryonic development and influence *in vitro* implantation (Massuto et al., 2010). Multiple studies have indicated that the proliferative activity of TE cells substantially affects the developmental ability of embryos. These findings underscore the significance of TGF-β as a key regulatory factor in embryo development and *in vitro* implantation, particularly through its effect on TE cell proliferation. Understanding the specific mechanisms by which TGF-β affects embryo development and *in vitro* implantation can potentially lead to advancements in IVP technology and enhance developmental outcomes.

Growth differentiation factor-8 (GDF-8) is a member of the TGF-β superfamily and is a myostatin subtype, primarily a secreted protein synthesized in skeletal muscle cells (McPherron et al., 1997). It is also expressed in the placenta, reproductive tissues, and cells (McPherron et al., 1997; Wong et al., 2009; Peiris and Mitchell, 2012; Peiris et al., 2014; Xie et al., 2020). Myostatin is a widely recognized inhibitor of muscle development that impedes cell proliferation and differentiation. However, it has been observed that both cell proliferation and migration are increased. Additionally, the elevated presence of myostatin in placental tissues during early pregnancy implies its potential involvement in early pregnancy, possibly contributing to the formation and development of the human placenta (Peiris et al., 2014). GDF-8 is expressed in extra villous trophoblasts and promotes the migration of trophoblasts (Peiris et al., 2014). Trophoblasts essential in regulating embryo development, *in vitro* implantation, and sustaining healthy pregnancies. In hamsters, GDF-8 induces proliferation exclusively in TE cells, stimulating the proliferation of extraembryonic trophoblast cells in preimplantation embryos and facilitating hatching (Wong et al., 2009), which underscores the significance of GDF-8 in regulating various aspects of embryonic development and *in vitro* implantation.

The GDF-8-related pathway involves the ALK5-SMAD2/3 signaling pathway. GDF-8 binds to a specific receptor, ALK5, in the trophoblast membrane. Upon binding of GDF-8 to its receptor, the receptor is activated, resulting in phosphorylation of SMAD2/3. Following phosphorylation, SMAD2/3 forms complexes with SMAD4 and relocates to the nucleus, where it conjugates to target genes, ultimately stimulating the expression of invasion-related genes (Peiris and Mitchell, 2012). The SMAD complex induces the transcription and expression of genes, such as follistatin-like 3 (*FSTL3*), matrix metalloproteinases 9 (*MMP9*), caudal-type homeobox 2 (*CDX2*), and SRY-box transcription factor 2, which increases TE cell proliferation, thereby enhancing their invasive capabilities (Yoon et al., 2019; Xie et al., 2020) (Supplementary Figure S1).

Cell invasion involves two primary processes: control of cell adhesion and migration, degradation of the extracellular matrix (ECM), and remodeling (Fang et al., 2021). Matrix metalloproteinases (MMPs) are also associated with ECM degradation. Matrix metalloproteinases 2 (*MMP2*)-mediated ECM degradation in humans enhances trophoblast cell invasion (Nagase and Woessner, 1999; Cohen et al., 2006). Furthermore, the expression of *MMP2* and *MMP9* is intricately linked to *in vitro* implantation capability, as it affects the capacity of embryos to degrade uterine epithelial ECM (Zhang et al., 2020). *FSTL3* binds to GDF-8, and its deficiency inhibits trophoblast invasion (Schneyer et al., 2004). It is also highly expressed in the human placenta (Xie et al., 2020). GDF-8 has been shown to modulate *FSTL3* expression in human trophoblasts by triggering *FSTL3* expression via the SMAD2/3 signaling pathway SMAD2/3 in human trophoblasts plays a role in upregulating GDF-8-induced *FSTL3* expression (Xie et al., 2020). Under hypoxic conditions, trophoblasts exhibit increased *FSTL3* expression, and the absence of *FSTL3* impedes trophoblast invasion (Xie et al., 2020). Upregulated *FSTL3* expression in trophoblasts under low-oxygen culture conditions is associated with regulating trophoblast functions, including apoptosis, invasion, lipid storage, and migration (Xie et al., 2020).

Tight junctions (TJ) and adherent junctions (AJ) determine the maintenance of TE cell integrity. Sustaining blastocoels is challenging when these structures undergo deformation or damage, and it becomes difficult to maintain the blastocoel (Sidrat et al., 2022). Additionally, the Na^+^/K^+^-ATPase pump involving the ATPase Na^+^/K^+^ Transporting Subunit Alpha 1 (*ATP1a1*) located at the basal membrane of the TE facilitates the movement of Na^+^ into the blastocoel through the TE, creating an osmotic pressure gradient that leads to fluid accumulation and blastocoel formation (Kidder, 2002; Barcroft et al., 2003; Barcroft et al., 2004). Furthermore, aquaporins (AQPs), membrane transporters, play a significant role in water transport (Edashige et al., 2007; Ribeiro et al., 2022). Previous studies have demonstrated that inhibition of AQPs suppresses blastocoel expansion, indicating their role in transporting water across the TE (Barcroft et al., 2003). Moreover, TJ, which seals the TE cells, is crucial for blastocyst formation. Incomplete sealing can result in fluid leakage around cells, reducing blastocoel size (Moriwaki et al., 2007). TJ seal spaces between adjacent cells (Tsukita et al., 2001; Schneyer et al., 2004). TJ comprises proteins, such as occludin and claudin, pivotal for structural integrity and barrier functionality (Wilcox et al., 2001; Ikenouchi et al., 2005; Moriwaki et al., 2007) (Supplementary Figure S2).

GDF-8 supplementation stimulates ALK5-SMAD2/3 signaling pathway activation, resulting in the upregulation of genes linked to implantation and TE cells. Activating this pathway augments the number of TE cells, enhancing *in vitro* implantation competence and functionality. This study investigated the enhancement of TE cell number and functionality through the ALK5-SMAD2/3 signaling pathway and its activation by supplementation with GDF-8, which led to improve *in vitro* implantation and developmental rates. The primary objective of this study was to confirm that the improved function of TE cells achieved through supplementation with GDF-8 enhances the functionality of AJ and TJ, thereby ensuring the preservation of post-thawing embryo survival and hatching rates.

In conclusion, adding GDF-8 to the culture medium could influence the quality of *in vitro* embryos, ultimately contributing to an improved *in vitro* implantation rate and TE cell function.

### 1.1 Experimental design

#### 1.1.1 Experiment 1

To confirm the effect of GDF-8 supplementation on IVP, one control group and four experimental groups (treated with 0.2, 2, 10, or 20 ng/mL GDF-8) were used to evaluate the optimal concentration in bovine embryos. After 8 d, the developmental and hatching rates in the control and experimental groups were evaluated.

#### 1.1.2 Experiment 2

To investigate whether GDF-8 supplementation affected TE cell proliferation, differential staining was performed using day 8 blastocysts, and variations in the mRNA levels of TE-related genes were analyzed in the control and GDF-8 groups.

#### 1.1.3 Experiment 3

Genes associated with cell invasion are crucial to regulating TE cell invasion. Hence, this study aimed to assess the impact of GDF-8 on invasion by stimulating the ALK5-SMAD2/3 signaling pathway, thereby upregulating the genes involved in cell invasion.

#### 1.1.4 Experiment 4

Experiments were conducted to evaluate the relative expression of TJ mRNA, AJ-related genes, and TJ permeability following GDF-8 supplementation. TJ assembly (claudin family, occludin, and actinγ2) was analyzed using day 8 blastocyst, and TJ permeability was investigated using a 4-kDa FITC-dextran assay after thawing blastocysts.

#### 1.1.5 Experiment 5

To determine the effect of GDF-8 supplementation during cryopreservation, blastocysts from the control and GDF-8 groups were recovered and cryopreserved on day 7. The survival rates were assessed at 24 and 48 h. The survival rate was evaluated based on the presence or absence of blastocoels in the cultured post-thaw blastocysts. The hatching rate was estimated at 24 h and 48 h. Furthermore, qRT-PCR was conducted to assess the relative expression of genes involved in the osmotic pressure gradient.

## 2 Materials and methods

All experiments were approved by the Animal Care Facility of the Gyeongsang National University Institute of Animal Care Committee (GNU-130902-A0059). No animals were used directly in this study, and all bovine ovaries for experiments were collected from local slaughterhouse. Recombinant human GDF-8, obtained from a mouse myeloma cell line (88-G8/CF), was purchased from R&D Systems (Minneapolis, MN, United States). Unless otherwise stated, all chemicals and reagents were purchased from Merck (Sigma–Aldrich, St. Louis, MO, United States).

### 2.1 Cumulus–oocyte complex (COC) recovery

Ovaries were collected from a slaughterhouse in Gimhae, immersed in a 0.9% NaCl solution at 37.5°C, and transferred to the laboratory within 1 h. After washing the ovaries with saline, COCs were retrieved from the follicles (2–8 mm in diameter) using an 18-gauge needle connected to a 10-mL disposable syringe. Aspirated follicle fluid was discharged into the 50-mL tube containing Tyrode lactate-HEPES [TL-HEPES, calcium chloride 2 mM (C-7902), HEPES 10 mM (H-6147), 100 IU/mL penicillin, phenol red 1 μL/mL (P-0290), potassium chloride 3.2 mM (P-5405), sodium bicarbonate 2 mM (S-5761), sodium biphosphate 0.34 mM (S-5011), sodium chloride 114 mM (S-5886), sodium lactate 10 mM (L-4263), and 0.1 mg/mL streptomycin] solution at 38.5°C and oocytes were recovered using a stereomicroscope. After washing with TL-HEPES, COCs with three layers of densely compacted cumulus cells were selected.

### 2.2 *In vitro* maturation (IVM)

The COCs were washed six times with IVM medium [Tissue Culture Medium-199 (TCM199, 11,150-059) with cysteine 0.6 mM, epidermal growth factor 10 ng/mL (EGF, E-4127), 10% (v/v) fetal bovine serum (16,000-044), follicle-stimulating hormone 10 μg/mL (HOR-285), 1 μg/mL estradiol-17β, and sodium pyruvate 0.2 mM (P-5280)] and the COCs, typically ranging from 30 to 35 in each well, were contained in 500 µL of IVM medium and then incubated at 38.5°C under a controlled humidified atmosphere of 5% CO_2_ for 22 h.

### 2.3 *In vitro* fertilization (IVF) and *in vitro* culture (IVC)

After IVM, the semen was thawed, washed with sperm Dulbecco’s phosphate-buffered saline, and then centrifuged at 750 × g for 5 min at 25°C. The sperm pellet was diluted with 500 µL of heparin (20 μg/mL) and incubated for 15 min at 38.5°C in humid conditions of 5% CO_2_. Subsequently, it was diluted with IVF medium (Tyrode lactate solution supplemented with 6 mg/mL BSA, 22 μg/mL sodium pyruvate, 100 IU/M penicillin, and 0.1 mg/mL streptomycin) to form 5 mL (final concentration of 1 × 10^6^ sperm/mL) and then matured COCs were transferred to a four-well dish containing sperm for 20 h in IVF medium. After IVF, the presumed zygotes from which the cumulus cells were removed were cultured in four-well dishes filled with 500 µL of synthetic oviductal fluid (SOF) medium (5 ng/mL ITS (insulin, transferrin, sodium selenite, 11074547001), 100 ng/mL EGF, and 4 mg/mL BSA) for 8 d (day 0 = day of IVF).

### 2.4 Supplementation of GDF-8

The solubility of GDF-8 is 4 mM HCl, and a 100 μg/mL GDF-8 stock solution was prepared by diluting 10 µg of GDF-8 in 100 µL of 4 mM HCl. Subsequently, SOF medium containing 10 ng/mL of GDF-8 used in the experiment was prepared by adding 0.5 µL of GDF-8 stock solution to 5 mL of SOF medium. The degree of development at each embryonic culture stage was compared between the control and GDF-8 groups.

### 2.5 Differential staining of blastocysts

Day 8 blastocysts were differentially stained to count ICM and TE cells. Blastocysts were permeabilized by a 20-s incubation in 0.2% Triton X-100 in PBS containing 2 mg/mL BSA, followed by two immediate washes in PBS-BSA solution (n = 5 per group). The ICM cells were stained by incubation in the dark at 37.5°C for 5 min with a PBS-BSA solution containing propidium iodide 30 μg/mL (PI, P-4864) and then washed twice in PBS-BSA solution. TE cells were stained by incubation in the dark for 30 min at 25°C with 4% paraformaldehyde fixation solution containing 10 μg/mL Hoechst 33,342 and washed twice in PBS-BSA solution. The stained samples were imaged using an Olympus IX71 microscope and analyzed using the ImageJ software (National Institutes of Health, Bethesda, MD, United States; https://imagej.nih.gov/ij).

### 2.6 Invasion assay

Day 8 hatched blastocysts were cultured in an invasion chamber insert (6.4 mm; Corning Inc. Life Sciences) to analyze the invasion area. The insert was coated with Matrigel (356,234) and incubated at 37.5°C for 2 h. The blastocysts were transferred to the chamber (three blastocysts per insert) and incubated at 37.5°C for 72 h, and the SOF medium was changed after 48 h. After 10 d of culture, the insert was washed thrice in PBS, fixed with 4% paraformaldehyde fixation solution at 4°C for 30 min, stained with 4’,6-diamidino-2-phenylindole (DAPI) for 5 min, and washed thrice with PBS. Images of the invasion area were captured using an Olympus IX71 microscope and analyzed using the ImageJ software.

### 2.7 Long term culture

To recovery the TE cells, Day 8 Hatched blastocysts were cultured in four-well dish. Four well dish was coated 2% gelatin and dried at least 2 h. The blastocysts were transferred to the well (Three blastocysts per well) and incubated at 37.5°C for 72 h in SOF medium and changed after 48 h. After blastocysts attached on plate beyond Day 8 and use the scrapper for gathering the attached cells.

### 2.8 Extraction of mRNA and complementary DNA (cDNA) synthesis

The manufacturer protocol was used to extract mRNA using the Arcturus PicoPure RNA Isolation Kit (Cat. No. 12204-01; Arcturus, Foster, United States). Day 8 blastocysts and TE cells were washed four times with nuclease-free water, placed in a 1.5 mL tube containing 30 µL nuclease-free water, frozen in liquid nitrogen, and stored at −80°C. The isolated mRNA samples were reverse-transcribed into single-stranded cDNA.

### 2.9 Quantitative reverse transcription polymerase chain reaction (qRT-PCR) analysis of target genes

The expression of the ALK5-SMAD2/3 signaling pathway, TJ, and AJ-related genes was investigated using qRT-PCR. The expression levels of these genes were normalized to that of glyceraldehyde-3-phosphate dehydrogenase (*GAPDH*). Primers designed based on the mRNA sequences of the genes were purchased from Macrogen (Seoul, Korea). qRT-PCR was performed using CFX98 devices (Bio-Rad Laboratories) with 1x iQ SYBR Green Supermix (iQ™SYBR^®^327Green 328 Supermix kit, Bio-Rad Laboratories) and 3 µL of diluted DNA. All cDNA samples were subjected to qRT-PCR to detect variations in the expression of other genes using GAPDH primers. After confirming that GAPDH expression was not significantly different between samples, all transcripts were quantified using qRT-PCR. The cycling conditions were as follows. 95°C for 3 min, 95°C for 15 s, 62°C for 20 s, 72°C for 30 s, and final extension for 5 min. Quantitative analysis was performed using the ^ΔΔ^Ct method. The results are reported as values relative to the mean value of the endogenous control, *GAPDH*, compared with the calibrator after normalization. The intra- and inter-assay variance coefficients were calculated using qRT-PCR for all genes. The primers sequences are mentioned in Table 1.

**TABLE 1:** List of qRT-PCR primers.

Gene name	Primer sequences	Accession number
MMP2	F: CCA​TTG​AGA​CCA​TGC​GGA​AG	NM_174745.2
	R: ACA​TCG​CTC​CAG​ACT​TGG​AA	
MMP9	F: CAC​GCA​CGA​CAT​CTT​TCA​GT	NM_174744.2
	R: TTC​AGG​AGG​TCG​AAG​GTC​AC	
FSTL3	F: GAT​GTA​CCG​TGG​TCG​CTG​C	NM_001075710.2
	R: GTA​GGT​GAC​GTT​GTT​GTT​GCC	
CDX2	F: TGA​GGA​GCA​TGG​ACT​CTG​CTA	NM_001206299.1
	R: GGG​CTA​GGT​CAG​CTG​GTA​AAC	
CLDN2	F: AGC​TAC​AGC​CAG​CAG​ACA​AG	NM_205781.2
	R: TGC​TGG​CAC​CAA​CAT​AGG​AG	
CLDN4	F: TGT​CCT​GGA​CGG​CTA​ACA​AC	NM_001014391.2
	R: TTA​GCG​GAG​TAG​GGC​TTG​TC	
OCLN	F: AAT​CAC​TAC​ACA​CCA​AGC​AAT​GAC	NM_001082433.2
	R: AAG​CAT​AGA​CAG​GAT​CCG​AAT​TAC	
ACTINγ2	F: CCA​CCA​TGT​ACC​CTG​GCA​TT	BT021005.1
	R: ACT​CTG​GCT​TGC​TGA​TCC​AC	
CD44	F: CCG​GAA​CAT​AGG​GTT​TGA​GA	NM_174013.3
	R: GGT​ATA​ACG​GGT​GCC​ATC​AC	
CDH1	F: CCG​TGA​GAG​TTT​TCC​CAC​AT	NM_001002763.1
	R: CAT​TGG​TGA​CTG​GGT​CTG​TG	
AQP3	F: AGA​AGG​AGC​TGG​TGA​ACC​G	NM_001079794.1
	R: ACA​GAG​CCA​CAG​CCA​AAC​AT	
AQP8	F: CAC​TGG​ATC​TAC​TGG​CTG​GG	NM_001206607.3
	R: CAG​GGG​AAG​CGT​ATC​AGT​CA	
AQP9	F: CTG​TTG​TCA​TTG​GCT​TCC​TG	NM_001205833.3
	R: AAC​CAA​AGG​TCC​CAC​TAC​AG	
ATP1a1	F: AAT​GCC​GAA​GTG​CTG​GAA​TC	NM_001076798.1
	R: GTC​CTG​GCG​AAC​ACA​ATC​TC	
ALK5	F: GGC​AGT​AAG​GCA​TGA​TTC​GG	NM_174621.2
	R: ATC​GTC​GAG​CTA​CTT​CCC​AG	
SMAD2	F: AGA​GAC​CTT​CCA​TGC​CTC​AC	NM_001046218.1
	R: CAC​TTA​GGC​ACT​CGG​CAA​AA	
SMAD3	F: AAC​CAT​GTC​GTC​CAT​CCT​G	NM_001205805.1
	R: CAG​TAG​ATG​ACG​TGG​GGG​AG	
GAPDH	F: CCC​AGA​ATA​TCA​TCC​CTG​CT	NM_001034034.2
	R: CTG​CTT​CAC​CAC​CTT​CTT​GA	

### 2.10 Cryopreservation and thawing procedure

An ethylene glycol-based freezing protocol and a conventional gold-standard freezing procedure were used for cryopreservation. Day 7 expanded blastocysts were immediately washed with a 0.5% (w/v) BSA solution prepared in PBS. After washing, the blastocysts were incubated with 0.1 M sucrose and 0.5% BSA solutions in an ethylene glycol cryoprotectant medium for 10 min. The blastocysts were then loaded into a 0.25-mL plastic straw. Embryos were slowly frozen using a controlled freezing system (CL-8800i; Cryo-Logic, Blackburn, Victoria, Australia). The straw was carefully transferred, immersed in liquid N_2_, and stored until further use. The thawing of frozen straw was exposed to air for 10 s and soaked in water at 37.5°C for 20 s. The thawed embryos were washed with SOF medium to remove the cryoprotectant medium and then cultured in a humidified atmosphere of 5% CO_2_ at 38.5°C for 48 h.

### 2.11 Assessment of embryo survival

After thawing, the survival rate was assessed by examining the presence or absence of blastocoels in the surviving blastocysts at 24 and 48 h. Additionally, the hatching rate of each group was evaluated 24 and 48 h after thawing.

### 2.12 Tight junction permeability assay

Post-thaw blastocysts were cultured in SOF medium for 24 h. The re-expanded blastocysts were stained with 1 mg/mL 4-kDa FITC-dextran (CAT# 60842-46-8, Sigma-Aldrich) in SOF medium at 25°C under dark conditions for 10 min and then washed with SOF medium. The fluorescence intensity that detected TJ permeability was measured using an Olympus IX71 microscope. The fluorescence intensity was analyzed using ImageJ software.

### 2.13 Immunofluorescence stain

Day 8 blastocysts were fixed using 4% paraformaldehyde fixation solution at 25°C for 30 min. Blastocysts were washed 3 times with 2% PBS-PVA and proteinase K-treated fixed blastocysts for 5 min to increase permeability. The blastocysts were washed 3 times and incubated with the blocking solution (5% BSA in PBS-PVA) at 25°C for 90 min. Then, the blastocysts were stained overnight with primary antibodies at 4°C. The blastocysts were washed 3 times with PBS-PVA and then incubated with TRITC secondary antibodies (Santa Cruz Biotechnology, Dallas, Texas, United States) at 25°C for 90 min. The nucleus was stained with DAPI at 25°C for 10 min. After washing, all blastocysts were mounted on slide glass. A laser scanning confocal microscope (Fluoview FV 1000; Olympus, Tokyo, Japan) was used for confocal imaging. Fluorescence intensities were measured using ImageJ software (National Institutes of Health, Bethesda, MD, United States; https://imagej.nih.gov/ij).

### 2.14 Statistical analysis

Statistical analysis was performed using one-way analysis of variance (ANOVA) with GraphPad Prism software (GraphPad Software, Franklin Street, Boston, MA; www.graphpad.com) and analyzed by multiple pairwise companies (Tukey’s test) for comparisons between groups. One-way ANOVA was used to compare embryonic development (blastocyst and hatching rates). Differential staining, invasion assays, and qRT-PCR were performed using randomly selected blastocysts from each group (five embryos per replicate). Triplicate sets of experiments were performed to analyze the data. Data concerning blastocyst quality (ICM, TE, TE:ICM ratio) and expression levels of various genes between the control and GDF-8 groups were compared using t-tests. The mean fluorescence intensities from all imaging data were quantified per blastocyst (n = 15–20) in each group. The ImageJ software (version: ij154) (National Institutes of Health, Bethesda, MD, United States) generated all histogram values. Data are expressed as the mean ± standard deviation (mean ± SE). *p < 0.05; **p < 0.01; ***p < 0.001 indicate a significant difference.

## 3 Results

### 3.1 Setting the concentration of GDF-8 and cleavage and developmental rates

We conducted experiments using a range of concentrations to determine the most optimal GDF-8 supplementation concentration. GDF-8 treatment groups were established using GDF-8 concentrations of 0.2, 2, 10, and 20 ng/mL. We observed a notable increase in both development and hatching rates (*p <* 0.05) when the GDF-8 group was set at 10 ng/mL (Control vs Treatment; 31.00 ± 1.82 vs 47.77 ± 3.52) (Control vs Treat; 7.04 ± 2.68 vs 28.04 ± 6.11; Table 2). GDF-8 supplementation to the SOF medium improved the development and hatching rates. Therefore, the GDF-8 treatment group was selected at a 10 ng/mL concentration, and the experimental process was performed accordingly.

**TABLE 2:** Effect of GDF-8 supplementation during *in vitro* culture on embryonic development of *in vitro* fertilization.

Treatments	No. of oocytes^a^	No. of presumed zygotes	No. of embryos cleaved (% ± SE)^b^	No. of blastocysts (% ± SE)^b^	No. of hatched blastocysts (% ± SE)^b^
Control	240	229	201 (87.77 ± 2.34)	71 (31.00 ± 1.82)^c^	5 (7.04 ± 2.68)^c^
0.2 ng/mL GDF-8	240	217	187 (86.18 ± 2.43)	83 (38.25 ± 2.69)^c^ ^,^ ^d^	13 (15.66 ± 4.36)^c^ ^,^ ^d^
2 ng/mL GDF-8	240	210	180 (85.71 ± 3.34)	93 (44.29 ± 2.82)^d^	21 (22.58 ± 3.72)^c^ ^,^ ^d^
10 ng/mL GDF-8	240	224	190 (84.82 ± 2.60)	107 (47.77 ± 3.52)^d^	30 (28.04 ± 6.11)^d^
20 ng/mL GDF-8	240	232	193 (83.19 ± 1.53)	66 (28.45 ± 1.28)^c^	16 (24.24 ± 3.96)^d^

^a^ Six replicates were performed.

^b^ Rates are relative to the number of presumed zygotes.

^c^ *p* < 0.05 with different superscripts indicates the significant difference.

^d^ *p* < 0.05 with different superscripts indicates the significant difference.

### 3.2 Effect of GDF-8 supplementation on the competence-related gene of trophectoderm cells

**FIGURE 1:**
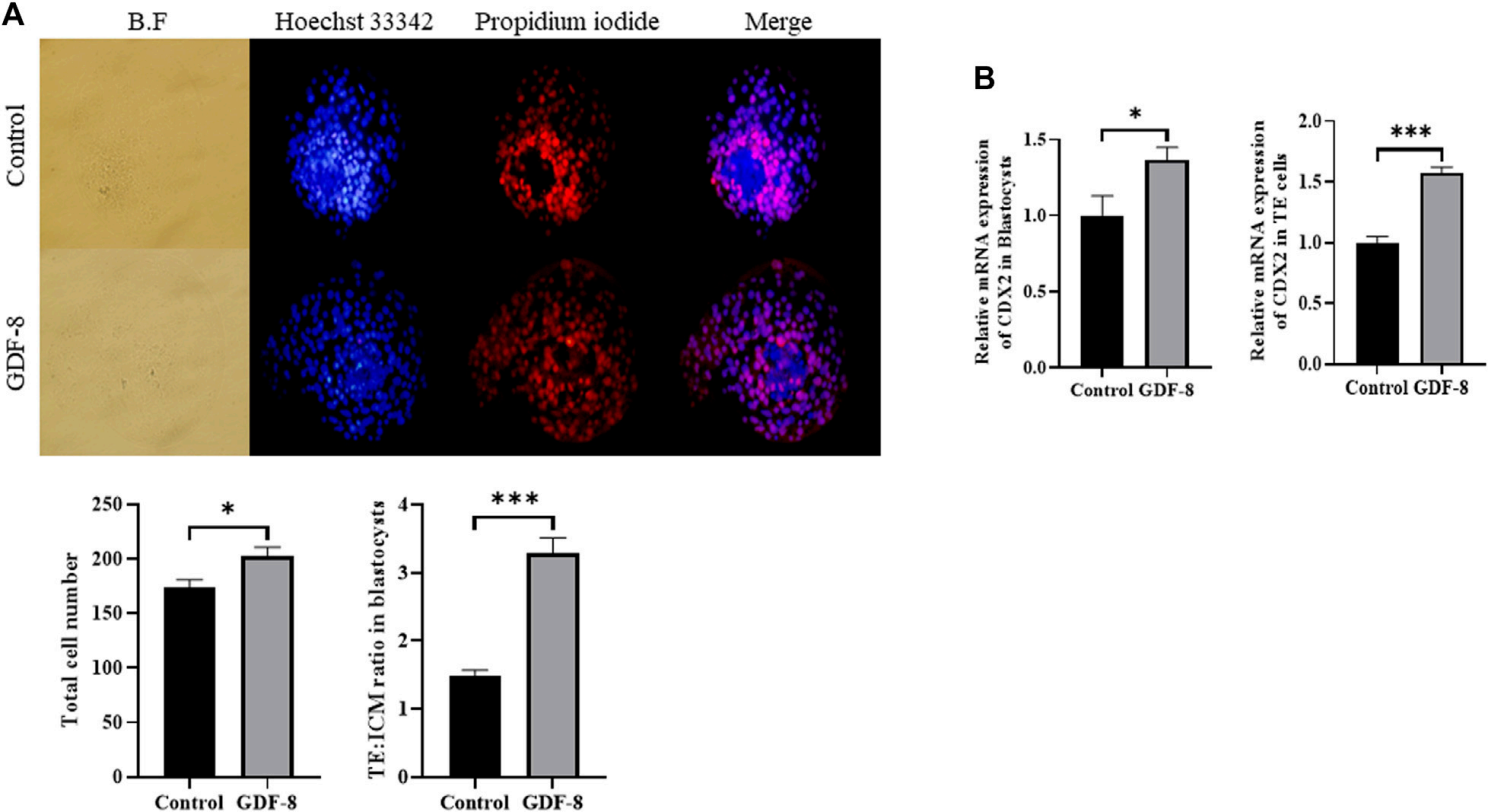
Number of ICM and TE cells in the control group and GDF-8 treatment group treated with Hoechst 33,342 and propidium iodide. **(A)** Differential stain of day 8 blastocysts showing propidium iodide localization in TE cells. Total cells were stained blue with Hoechst 33,342. TE cells were stained red with propidium iodide. Images were obtained using an epifluorescence microscope. Total cell number and TE: ICM cell ratio. Day 8 blastocysts were quantified after differential staining. Original magnification: ×200. Values represent the mean ± SE (n = 20, *p < 0.05, ***p < 0.001). **(B)** Relative mRNA expression levels of *CDX2* related to trophectoderm cell proliferation in the control and GDF-8 groups of blastocysts and TE cells. Three replicates were performed. Values represent the mean ± SE (*p < 0.05, ***p < 0.001). BF, bright field; GDF-8, 10 ng/mL GDF-8; ICM, inner cell mass; TE, trophectoderm.

The GDF-8 treatment group showed significantly improved total cell numbers (*p* < 0.05) compared to the control group. Moreover, the control group exhibited more TE cells than did GDF-8 group, leading to a significant difference (*p <* 0.01) in the ratio of ICM cells to TE cell numbers between the control and GDF-8 groups (Figure 1A). Furthermore, blastocysts and TE cells treated with GDF-8 had significantly higher (Control, Treat; *p <* 0.05, *p* < 0.001) mRNA levels of *CDX2* (Figure 1B). These findings suggest that the addition of GDF-8 to the SOF medium stimulates TE cell proliferation by enhancing the expression of genes associated with TE cells.

### 3.3 GDF-8 supplementation improved *in vitro* implantation ability and related genes

**FIGURE 2:**
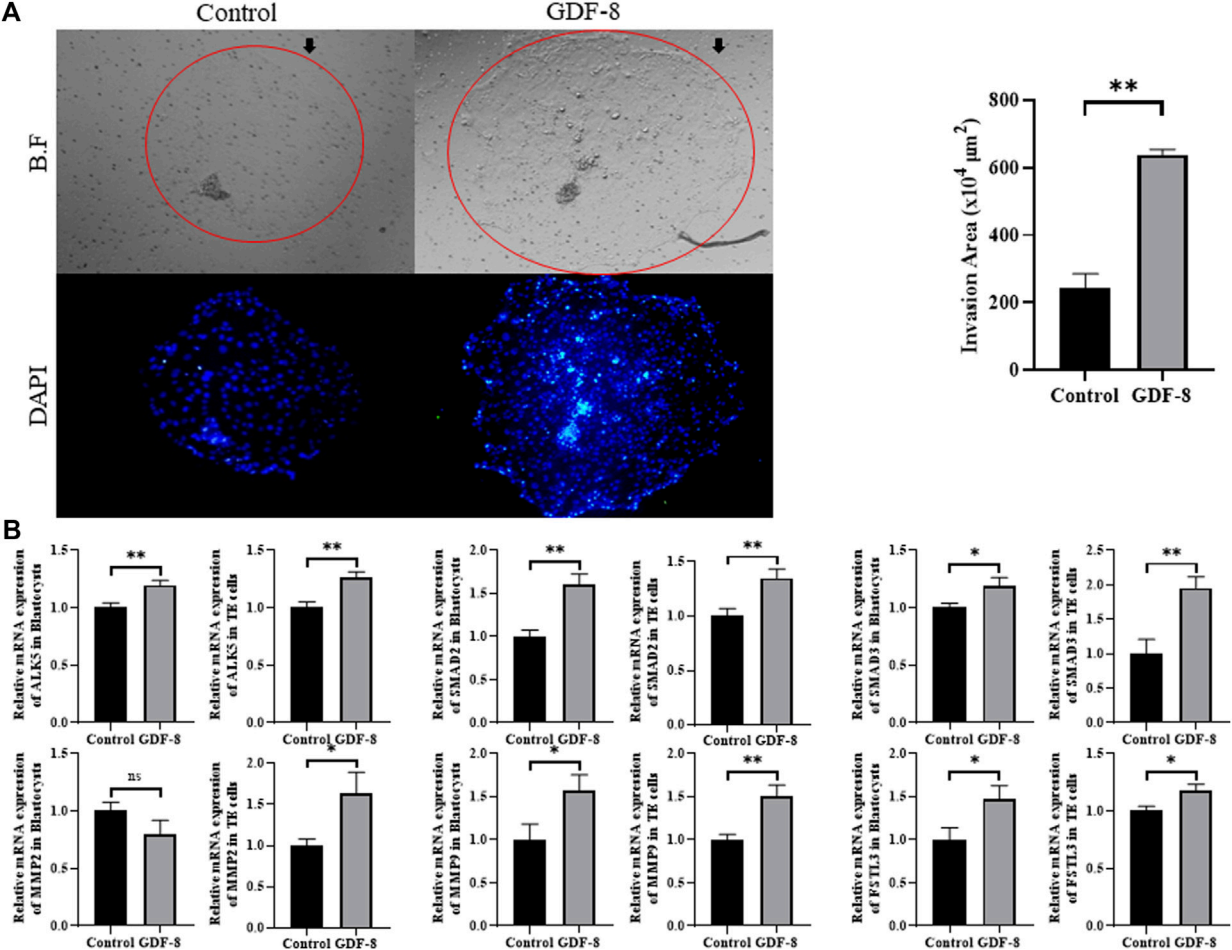
Invasion ability of trophoblast. **(A)** Bright-field and DAPI image showing the invasion area in the control and GDF-8 groups. Original magnification: ×40. Mean invasion area analyzed using ImageJ. n = 3 blastocysts per group were used in triplicates. **(B)** Relative mRNA expression level of ALK5, SMAD2 and SMAD3 related with ALK5-SMAD2/3 signaling pathway, *MMP2*, *MMP9* and *FSTL3* related to implantation in control and GDF-8 groups of blastocysts and TE cells. Three replicates were performed. Values represent the mean ± SE. (ns = not significant, *p < 0.05, **p < 0.01). BF, bright field; DAPI, 4′,6-diamidino-2-phenylindole; GDF-8, 10 ng/mL GDF-8.

The area of trophoblast invasion, assessed using ImageJ software, was significantly increased (*p <* 0.01) in the GDF-8 group compared to that in the control group (Figure 2A). Furthermore, we investigated the expression of genes associated with the ALK5-SMAD2/3 signaling pathway. The low *in vitro* implantation ability of IVC blastocysts has been attributed to a decrease in the ability and/or function of the TE cells. qRT-PCR analysis demonstrated significant upregulation (*p <* 0.05, *p <* 0.01) in the mRNA expression of *ALK5*, *SMAD2*, and *SMAD3* in the GDF-8 group compared to that in the control group (Figure 2B). Moreover, the mRNA expression of *ALK5*, *SMAD2*, and *SMAD3* were significantly higher (*p* < 0.01) in the GDF-8 treated TE cells compared to that in the control group (Figure 2B). Therefore, GDF-8 supplementation stimulated activation of the ALK5-SMAD2/3 signaling pathway. Moreover, the mRNA expression of *MMP9* and *FSTL3*, both linked to the ALK5-SMAD2/3 signaling pathway, was significantly upregulated (*p <* 0.05) in the GDF-8 group compared with that in the control group. These genes’ increased relative mRNA levels suggest that supplementation with GDF-8 stimulates the ALK5-SMAD2/3 signaling pathway, thereby improving *in vitro* implantation ability. However, *MMP2* levels were not significantly different (Figure 2B). The mRNA expression of *MMP2*, *MMP9*, and *FSTL3* related to invasion ability, were significantly increased (*p* < 0.05, *p* < 0.01) in the GDF-8 treated TE cells compared to that in the control group (Figure 2B). These findings indicate that supplementation of GDF-8 to SOF medium stimulates the ALK5-SMAD2/3 signaling pathway and enhances *in vitro* implantation gene expression.

### 3.4 GDF-8 supplementation improved TJ complexes and AJ in blastocysts

**FIGURE 3:**
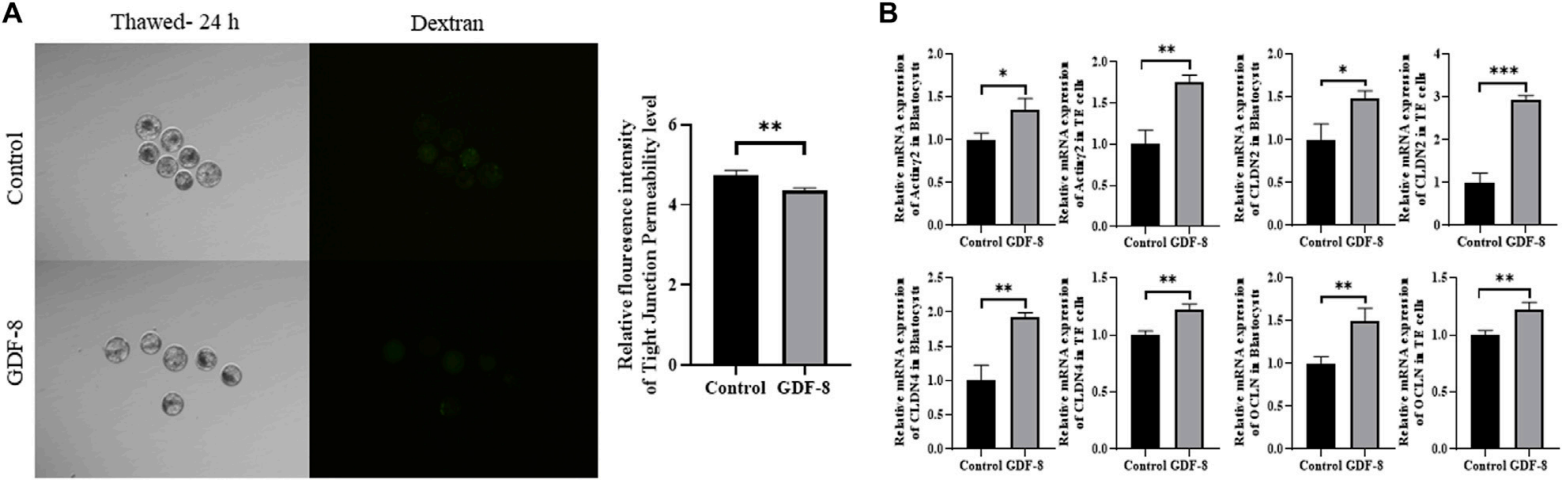
Tight junction sealing assay in post-thawing blastocyst cultures in control and GDF-8 groups. **(A)** Representative images of post-thawed blastocysts cultured for 24 h in the control and GDF-8 groups and then subjected to a dextran assay to assess tight junction sealing. Original magnification: ×100. Fluorescent signal in blastocysts cultured in the control and GDF-8 groups and then treated with dextran for 24 h n = 3 blastocysts per group were used in triplicates. **(B)** Relative mRNA levels of *Actinγ2*, *CLDN2*, *CLDN4*, and *OCLN* in the control and GDF-8 groups of blastocysts and TE cells. Three replicates were performed. Values represent the mean ± SE (*p < 0.05; **p < 0.01). Dexran, 4 kDa FITC-labelled dextran; GDF-8, 10 ng/mL GDF-8.

**FIGURE 4:**
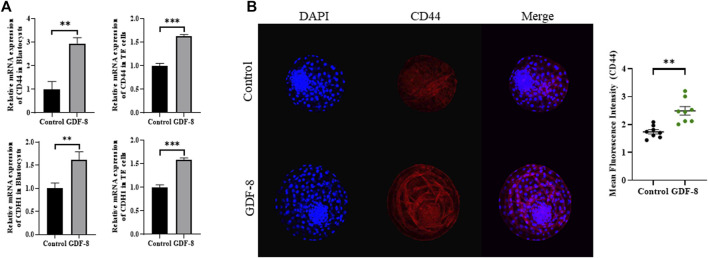
Expression of Adherent junction protein in control and GDF-8 groups. **(A)** Relative mRNA level of *CD44*, and *CDH1* in control and GDF-8 groups of blastocysts and TE cells. Three replicates were performed. Values represent the mean ± SE (*p < 0.05; **p < 0.01). **(B)** Confocal images of immunofluorescence CD44 in control and GDF-8 groups. Original magnification: ×200. The quantification of signal intensities of CD44 expression. n = 3 blastocysts per group were used in triplicates. DAPI, 4′,6-diamidino-2-phenylindole; GDF-8, 10 ng/mL GDF-8.

Maintenance of AJ and TJ integrity within the inner layer of TE epithelium is paramount in developing expanded blastocysts. Following a 24 h culture post-thawing, blastocysts were assessed via 4-kDa FITC-dextran analysis, and a significant reduction in fluorescence was observed within the GDF-8 group relative to the control group. (*p <* 0.01). This decrease indicates a significant reduction in the permeability of TJ in the blastocysts after GDF-8 treatment (*p <* 0.01) (Figure 3A). Moreover, qRT-PCR analysis offered further confirmation, showing that the mRNA expression of genes regulated by TJ (*actinγ2*, *CLDN2*, *CLDN4*, and *OCLN*) was significantly elevated in the GDF-8 group relative to that in the control group (*p <* 0.05) (Figure 3B). Moreover, the mRNA expression of the gene related to TJ (*actinγ2*, *CLDN2*, *CLDN4*, and *OCLN*) was significantly improved (*p* < 0.01, *p* < 0.001) in the GDF-8 treated TE cells compared to that in the control group (Figure 3B). Furthermore, the mRNA expression levels of genes related to AJ (*CD44* and *CDH1*) were significantly higher in the GDF-8 group than those in the control group (*p <* 0.05) (Figure 3B; Figure 4A). In addition, the mRNA expression level of gene related to AJ (*CD44* and *CDH1*) was higher (*p* < 0.001) in the GDF-8 treated TE cells compared to that in the control group (Figure 4B). The CD44 protein level was higher in the GDF-8 group than those in the control group (*p* < 0.01) (Figure 4B). These results imply that including GDF-8 in the SOF medium positively influences gene expression associated with AJ and TJ, contributing to the observed alterations in trophoblast cell morphology and function.

### 3.5 Effect of GDF-8 supplementation on post-thaw blastocysts

**FIGURE 5:**
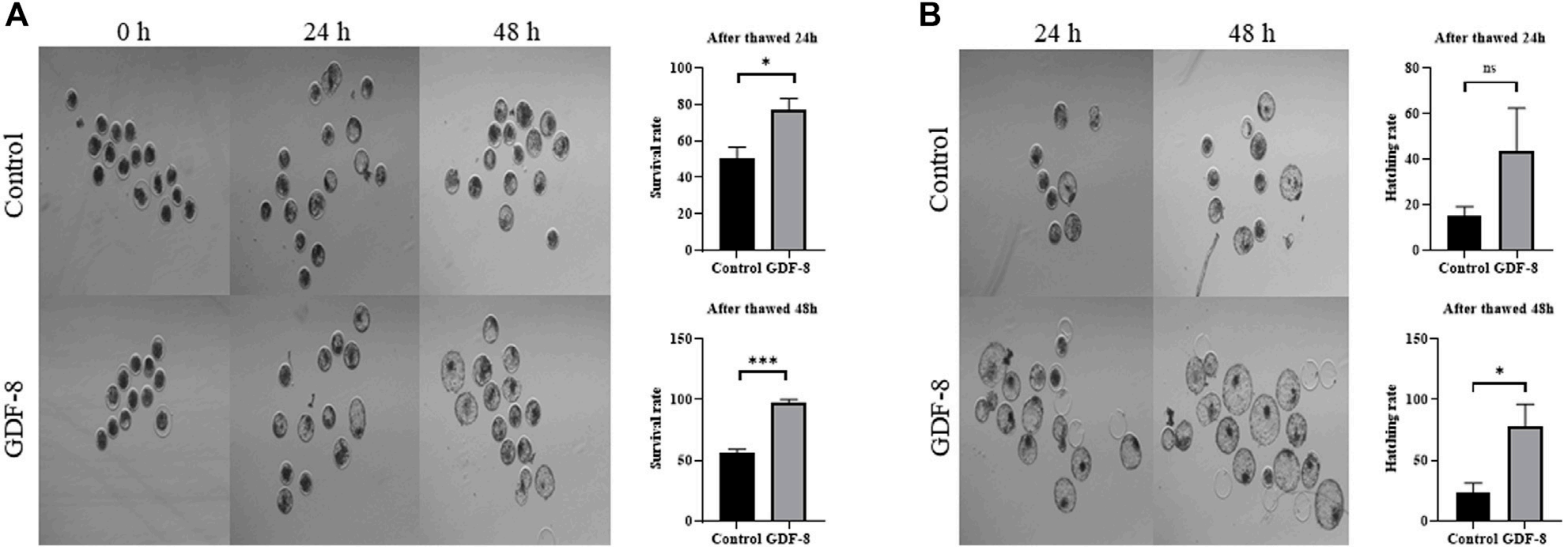
Survival and hatching in post-thawed bovine blastocysts cultured in the control and GDF-8 groups. **(A)** Representative images of surviving blastocysts cultured in the control and GDF-8 groups after 24 and 48 h of thawing. Original magnification: ×64. After thawing, investigate the survival rate. Three replicates were performed. Values represent the mean ± SE (ns = not significant, *p < 0.05; ***p < 0.001). **(B)** Representative images of hatching blastocysts cultured in the control and GDF-8 groups after 24 and 48 h of thawing. After thawing, investigate the hatching rate. Three replicates were performed. Values represent the mean ± SE (ns = not significant, *p < 0.05; ***p < 0.001). Original magnification: ×64. GDF-8, 10 ng/mL GDF-8.

**FIGURE 6:**
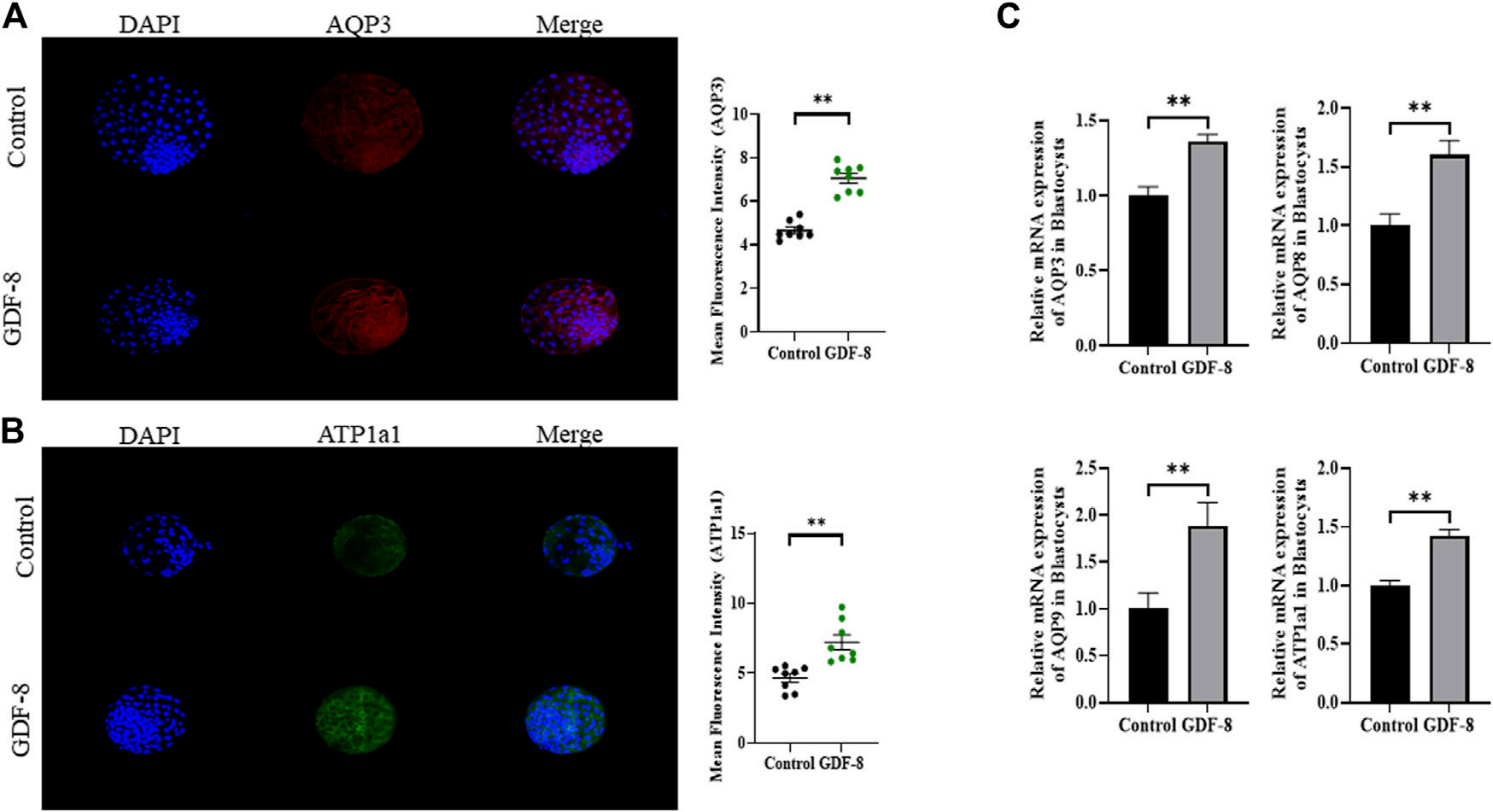
Expression of blastocoel expansion protein in control and GDF-8 groups. **(A)** Confocal images of immunofluorescence AQP3 in control and GDF-8 groups. Original magnification ×200. The quantification of signal intensities of AQP3 and expression. (n = 8). **(B)** Confocal images of immunofluorescence ATP1a1 in control and GDF-8 groups. Original magnification ×200. The quantification of signal intensities of ATP1a1 expression. (n = 8). **(C)** Relative mRNA expression levels of AQP3, AQP8, AQP9, and ATP1a1 related to re-expansion in the control and GDF-8 groups. Three replicates were performed. Values represent the mean ± SE (**p < 0.01). DAPI, 4′,6-diamidino-2-phenylindole; GDF-8, 10 ng/mL GDF-8.

When assessing the blastocoel re-expansion rate at 12 h post-thawing, it was observed that the GDF-8-treated group exhibited blastocysts with over 70% re-expanded blastocoel cavities compared to the control group (Figures 5A,B). Blastocysts treated with GDF-8 exhibited significantly higher blastocoel expansion 24 and 48 h post-thawing, indicating improved survival rates (Figure 5A). Furthermore, the hatching rate was significantly higher (*p <* 0.05) 48 h post-thawing in the GDF-8 group than that in the control group (Figure 5B). While upregulating AQP3, ATP1a1 protein level in GDF-8 group those in the control group (*p* < 0.01) (Figures 6A,B). Additionally, in blastocysts that survived post-thawing, mRNA expression of genes related to *AQP3*, *AQP8*, *AQP9*, and *ATP1a1* was significantly upregulated in blastocysts treated with GDF-8 (Figure 6C). This suggests that GDF-8 supplementation promotes the upregulation of genes associated with *AQPs*, leading to increased fluid accumulation, thus implicating GDF-8 in influencing the post-thaw re-expansion and hatching rates.

## 4 Discussion

IVP is a critically assisted reproductive technology that is commercially employed to induce genetic improvement (Thibier, 2005). Over the years, concerted research efforts have been dedicated to overcoming several inherent constraints of IVP. Nevertheless, these limitations persist and substantially influence embryonic development and implantation rates (Thibier, 2005; Moussa et al., 2014). Consequently, this study was designed to investigate the impact of GDF-8 supplementation on the activation of the ALK5-SMAD2/3 signaling pathway in bovine *in vitro* embryo production and embryonic quality. This study examined the proliferation and functionality of TE cells, particularly their effects on implantation and cryopreservation *in vitro*.

GDF-8, classified as a member of the TGF-β superfamily, has been acknowledged as an activator of the ALK5-SMAD2/3 signaling pathway (Fang et al., 2021). Additionally, it has been demonstrated that activating this signaling pathway stimulates the expression of *MMP2*, thereby mediating the upregulation of invasive capabilities in human extra villous trophoblast cells (Fang et al., 2021). Moreover, it facilitates trophoblast mobility, regulates invasion, and promotes the expression of FSTL3, thereby influencing invasive capabilities (Xie et al., 2020; Fang et al., 2021; Chen et al., 2023). According to previous studies, GDF-8 binds to the receptor ALK5, activating the SMAD2/3 complex. Subsequently, the SMAD complex is transferred to the nucleus to activate implantation-related genes (Zheng et al., 2022). Our research involved the administration of GDF-8, resulting in the activation of the ALK5-SMAD2/3 signaling pathway, and an assessment of the mRNA expression of genes related to cellular invasion, specifically *ALK5*, *SMAD2*, *SMDA3*, *MMP2*, *MMP9*, and *FSTL3*. Notably, we demonstrated that GDF-8 supplementation stimulated the ALK5-SMAD2/3 signaling pathway. Moreover, we detected a significant increase in the mRNA expression levels of *MMP9* and *FSTL3* in the GDF-8-treated group. Furthermore, invasion assays confirmed that the *in vitro* implantation ability of GDF-8-treated embryos was significantly enhanced.

Previous studies have reported that GDF-8 treatment of hamster blastocysts promotes TE cell proliferation (Wong et al., 2009). Additionally, previous studies have established that the ratio of ICM to total cell number is reduced *in vitro* blastocysts cultured *in vitro* compared to those cultured *in vivo* (Koo et al., 2002). In our study, we conducted a differential staining analysis of control and GDF-8-treated blastocysts. In GDF-8 treated blastocysts, we observed enhanced proliferation of TE cells, and the ICM:total cell number ratio was approximately 20%, similar to that observed in blastocysts cultured *in vivo*. One previous study stated that Wnt signaling activator can increase the TE:ICM ratio (Denicol et al., 2014). Few other studies also claimed variation in TE:ICM ratio, using various conditions (Sakatani et al., 2008; Wooldridge and Ealy, 2019), but mostly the number is between 2.9 and 3.5. *In vivo* produced bovine embryos is the standard example and several researches claimed that difference is present between *in vivo* and *in vitro* produced bovine embryos in TE:ICM ratio (Iwasaki et al., 1990). *CDX2* initiates the differentiation of early TE cells and plays a crucial role in TE function and maintenance (Wu et al., 2010). Furthermore, previous studies have indicated that *CDX2* is essential for the integrity of the AJ and TJ in the TE epithelium surrounding the blastocoel of expanded mouse blastocysts (Strumpf et al., 2005; Qu et al., 2017). We observed a significant increase in the mRNA expression level of *CDX2* in GDF-8-treated blastocysts. Therefore, the upregulation of *CDX2* expression was facilitated by GDF-8 supplementation, which enhanced the proliferation and functionality of TE cells.

Previous studies have discovered that TJ and AJ play a crucial role in maintaining the integrity of TJ during cryo-shrinkage (Sidrat et al., 2022). TJ functions as a gate for the transport of ions and small molecules, whereas AJ provides structural support (Eckert and Fleming, 2008; Marikawa and Alarcon, 2012). When these components are compromised during cryo-shrinkage, TJ integrity is not maintained, resulting in ineffective sealing of TJ post-thawing (Sidrat et al., 2022). This difficulty in sealing prevents fluid accumulation within the blastocoel, making re-expansion difficult. Therefore, the development of the TE epithelium plays a pivotal role in blastocoel formation, and effective sealing of TJ regulates the balance between osmotic pressure gradients and fluid accumulation within the blastocoel (Chan et al., 2019). In the event of damage during cryo-shrinkage, TJ integrity is disrupted, leading to inadequate sealing (Miller et al., 2003). Therefore, fluid accumulation within the blastocoel after thawing becomes problematic and hinders re-expansion.

Therefore, we assess their permeability by conducting a 4-kDa FITC-dextran assay on control and GDF-8-treated blastocysts. We observed significant maintenance of TJ integrity in GDF-8-treated blastocysts at 24 h post-thawing. Additionally, AJ in TE cells is associated with TE integrity and is substantially affected by cryo-shrinkage. Genes related to AJ, such as *CDH1* and *CD44*, were upregulated in GDF-8-treated blastocysts. *CD44* is also expressed in the embryo (Wheatley et al., 1993; Haegel et al., 1994; Campbell et al., 1995) and associated with embryonic attachment during the early stages of implantation (Berneau et al., 2019). To confirm that the expression of *CD44* is related to AJ, immunofluorescence staining was performed, and the *CD44* protein level in GDF-8-treated blastocyst was upregulated. Therefore, GDF-8 supplementation has the potential to sustain accumulated fluid pressure during blastocoel re-expansion after thawing (Kirschner et al., 2011; Reardon et al., 2012). Furthermore, mRNA expression levels in GDF-8-treated blastocysts of genes associated with TJ and blastocoel re-expansion, including *CLDN2*, *CLDN4*, *OCLN*, and *actinγ2*, were upregulated (Eckert and Fleming, 2008). Thus, supplementation of GDF-8 induces increased AJ- and TJ-related gene expression within blastocysts, reducing cellular sealing defects.

One of the factors promoting blastocoel formation is the osmotic pressure gradient facilitated by *AQPs* and *ATP1a1*, which regulate the osmotic pressure and water transport pathways (Robinson and Benos, 1991; Frank et al., 2019; Kosyl and Ajduk, 2023). Therefore, we examined the expression of *AQP3*, *AQP8*, *AQP9*, and *ATP1a1* to assess the blastocyst blastocoel formation ability. A substantial increase in gene expression was observed in the GDF-8-treated blastocysts. *AQP3* is an H_2_O transport channel located on the basolateral membrane of the TE (Johnston et al., 2000; Bell and Watson, 2013). This channel facilitates water transfer into the blastocyst cavity, establishing an osmotic gradient accumulating fluid (Barcroft et al., 2003). Additionally, *ATP1a1* elevates the intracellular osmotic pressure, contributing to fluid accumulation in the blastocoel (Barcroft et al., 2003; Bell and Watson, 2013). We conducted immunofluorescence experiments to determine the expression of the genes related to blastocyst formation, *AQP3*, and *ATP1a1*. *AQP3* and *ATP1a1* protein levels in GDF-8-treated blastocyst were upregulated. In addition, post-thaw GDF-8-treated blastocysts exhibited significantly higher survival and hatching rates. The post-thaw survival and hatching rates of blastocysts also increased with increasing expression levels of *AQPs* and *ATP1a1*. The supplementation of GDF-8 to the IVC culture medium positively impacted the development and hatching rates of bovine IVP embryos, which significantly enhanced cryo-tolerance and *in vitro* implantation by stimulating TE cell function and proliferation.

## Data Availability

The original contributions presented in the study are included in the article/Supplementary Material, further inquiries can be directed to the corresponding authors.

## References

[B1] Adu-Gyamfi E. A. Ding Y.-B. Wang Y.-X. (2020). Regulation of placentation by the transforming growth factor beta superfamily. Biol. Reproduction 102, 18–26. 10.1093/biolre/ioz186 31566220

[B2] Barcroft L. C. Offenberg H. Thomsen P. Watson A. J. (2003). Aquaporin proteins in murine trophectoderm mediate transepithelial water movements during cavitation. Dev. Biol. 256, 342–354. 10.1016/s0012-1606(02)00127-6 12679107

[B3] Barcroft L. Moseley A. Lingrel J. Watson A. (2004). Deletion of the Na/K-ATPase alpha1-subunit gene (Atp1a1) does not prevent cavitation of the preimplantation mouse embryo. Mech. Dev. 121, 417–426. 10.1016/j.mod.2004.04.005 15147760

[B4] Bell C. E. Watson A. J. (2013). p38 MAPK regulates cavitation and tight junction function in the mouse blastocyst. PloS one 8, e59528. 10.1371/journal.pone.0059528 23593143 PMC3617173

[B5] Berneau S. Ruane P. Brison D. R. Kimber S. Westwood M. Aplin J. (2019). Investigating the role of CD44 and hyaluronate in embryo-epithelial interaction using an *in vitro* model. MHR Basic Sci. reproductive Med. 25, 265–273. 10.1093/molehr/gaz011 30865276

[B6] Camargo L. D. A. Viana J. Sã W. Ferreira A. Ramos A. Vale Filho V. (2018). Factors influencing *in vitro* embryo production. Anim. Reprod. Ar. 3, 19–28.

[B7] Camargo L. S. A. Boite M. C. Wohlres-Viana S. Mota G. B. Serapiao R. V. Sa W. F. (2011). Osmotic challenge and expression of aquaporin 3 and Na/K ATPase genes in bovine embryos produced *in vitro* . Cryobiology 63, 256–262. 10.1016/j.cryobiol.2011.09.135 21985766

[B8] Camargo L. S. A. Ferreira A. M. Ramos A. A. Vale Filho V. R. (2006). Factors influencing *in vitro* embryo production. Anim. Reprod. 3, 19.

[B9] Campbell S. Swann H. Aplin J. Seif M. Kimber S. Elstein M. (1995). Fertilization and early embryology: CD44 is expressed throughout pre-implantation human embryo development. Hum. Reprod. 10, 425–430. 10.1093/oxfordjournals.humrep.a135955 7539449

[B10] Chan C. J. Costanzo M. Ruiz-Herrero T. MøNKE G. Petrie R. J. Bergert M. (2019). Hydraulic control of mammalian embryo size and cell fate. Nature 571, 112–116. 10.1038/s41586-019-1309-x 31189957

[B11] Chen J. Song T. Yang S. Meng Q. Han X. Wu Z. (2023). Snail mediates GDF-8-stimulated human extravillous trophoblast cell invasion by upregulating MMP2 expression. Cell Commun. Signal. 21, 93–13. 10.1186/s12964-023-01107-2 37143106 PMC10158255

[B12] Choi I. Carey T. S. Wilson C. A. Knott J. G. (2012). Transcription factor AP-2γ is a core regulator of tight junction biogenesis and cavity formation during mouse early embryogenesis. Development 139, 4623–4632. 10.1242/dev.086645 23136388 PMC3518458

[B13] Cohen M. Meisser A. Bischof P. (2006). Metalloproteinases and human placental invasiveness. Placenta 27, 783–793. 10.1016/j.placenta.2005.08.006 16249026

[B14] Corcoran D. Fair T. Park S. Rizos D. Patel O. Smith G. (2006). Suppressed expression of genes involved in transcription and translation in *in vitro* compared with *in vivo* cultured bovine embryos. Reproduction 131, 651–660. 10.1530/rep.1.01015 16595716

[B15] Denicol A. C. Block J. Kelley D. E. Pohler K. G. Dobbs K. B. Mortensen C. J. (2014). The WNT signaling antagonist Dickkopf-1 directs lineage commitment and promotes survival of the preimplantation embryo. FASEB J. 28, 3975–3986. 10.1096/fj.14-253112 24858280 PMC5395727

[B16] Eckert J. J. Fleming T. P. (2008). Tight junction biogenesis during early development. Biochimica Biophysica Acta (BBA)-Biomembranes 1778, 717–728. 10.1016/j.bbamem.2007.09.031 18339299

[B17] Edashige K. Ohta S. Tanaka M. Kuwano T. Valdez D. M. JR Hara T. (2007). The role of aquaporin 3 in the movement of water and cryoprotectants in mouse morulae. Biol. reproduction 77, 365–375. 10.1095/biolreprod.106.059261 17429015

[B18] Fang L. Wang Z. Wu Z. Yan Y. Gao Y. Li Y. (2021). GDF-8 stimulates trophoblast cell invasion by inducing ALK5-SMAD2/3-mediated MMP2 expression. Reproduction 162, 331–338. 10.1530/REP-21-0197 34432647

[B19] Frank L. Rose R. Anastasi M. Tan T. Barry M. Thompson J. (2019). Artificial blastocyst collapse prior to vitrification significantly improves Na+/K+-ATPase-dependent post-warming blastocoel re-expansion kinetics without inducing endoplasmic reticulum stress gene expression in the mouse. Reproduction, Fertil. Dev. 31, 294–305. 10.1071/RD17500 30099982

[B20] Gauster M. Moser G. Wernitznig S. Kupper N. Huppertz B. (2022). Early human trophoblast development: from morphology to function. Cell. Mol. Life Sci. 79, 345. 10.1007/s00018-022-04377-0 35661923 PMC9167809

[B21] Haegel H. Dierich A. Ceredig R. (1994). CD44 in differentiated embryonic stem cells: surface expression and transcripts encoding multiple variants. J. Immunol. Res. 3, 239–246. 10.1155/1994/25484 PMC2275938 7542511

[B22] Ikenouchi J. Furuse M. Furuse K. Sasaki H. Tsukita S. Tsukita S. (2005). Tricellulin constitutes a novel barrier at tricellular contacts of epithelial cells. J. Cell Biol. 171, 939–945. 10.1083/jcb.200510043 16365161 PMC2171318

[B23] Iwasaki S. Yoshiba N. Ushijima H. Watanabe S. Nakahara T. (1990). Morphology and proportion of inner cell mass of bovine blastocysts fertilized *in vitro* and *in vivo* . Reproduction 90, 279–284. 10.1530/jrf.0.0900279 2231548

[B24] Johnston H. Koukoulas I. Jeyaseelan K. Armugam A. Earnest L. Baird R. (2000). Ontogeny of aquaporins 1 and 3 in ovine placenta and fetal membranes. Placenta 21, 88–99. 10.1053/plac.1999.0445 10692256

[B25] Kaidi S. Bernard S. Lambert P. Massip A. Dessy F. Donnay I. (2001). Effect of conventional controlled-rate freezing and vitrification on morphology and metabolism of bovine blastocysts produced *in vitro* . Biol. Reproduction 65, 1127–1134. 10.1095/biolreprod65.4.1127 11566734

[B26] Khurana N. K. Niemann H. (2000). Energy metabolism in preimplantation bovine embryos derived *in vitro* or *in vivo* . Biol. reproduction 62, 847–856. 10.1095/biolreprod62.4.847 10727252

[B27] Kidder G. M. (2002). Trophectoderm development and function: the roles of Na+/K+-ATPase subunit isoforms. Can. J. physiology Pharmacol. 80, 110–115. 10.1139/y02-017 11934253

[B28] King W. Xu K. Sirard M. A. Greve T. Leclerc P. Lambert R. (1988). Cytogenetic study of parthenogenetically activated bovine oocytes matured *in vivo* and *in vitro* . Gamete Res. 20, 265–274. 10.1002/mrd.1120200303 3235040

[B29] Kirschner N. Haftek M. Niessen C. M. Behne M. J. Furuse M. Moll I. (2011). CD44 regulates tight-junction assembly and barrier function. J. Investigative Dermatology 131, 932–943. 10.1038/jid.2010.390 21191420

[B30] Koo D. B. Kang Y. K. Choi Y. H. Park J. S. Kim H. N. Oh K. B. (2002). Aberrant allocations of inner cell mass and trophectoderm cells in bovine nuclear transfer blastocysts. Biol. Reprod. 67, 487–492. 10.1095/biolreprod67.2.487 12135886

[B31] Kosyl E. Ajduk A. (2023). O-163 the influence of aquaporin 3 on dynamics of cavitation, implantation and re-expansion after vitrification/warming. Hum. Reprod. 38, dead093–196. 10.1093/humrep/dead093.196

[B32] Lechniak D. Cieslak D. Sosnowski J. (1997). Cytogenetic analysis of bovine parthenotes after spontaneous activation *in vitro* . Theriogenology 49, 779–785. 10.1016/S0093-691X(98)00027-2 10732086

[B33] Marikawa Y. Alarcon V. B. (2012). Creation of trophectoderm, the first epithelium, in mouse preimplantation development. Mouse Dev. Oocyte Stem Cells 55, 165–184. 10.1007/978-3-642-30406-4_9 PMC3642205 22918806

[B34] Marsico T. V. Camargo J. D. Valente R. S. Sudano M. J. (2019). Embryo competence and cryosurvival: molecular and cellular features. Anim. Reprod. 16, 423–439. 10.21451/1984-3143-AR2019-0072 32435286 PMC7234140

[B35] Massuto D. A. Kneese E. C. Johnson G. A. Burghardt R. C. Hooper R. N. Ing N. H. (2010). Transforming growth factor beta (TGFB) signaling is activated during porcine implantation: proposed role for latency-associated peptide interactions with integrins at the conceptus-maternal interface. Reproduction 139, 465–478. 10.1530/REP-09-0447 19920116

[B36] Mcpherron A. C. Lawler A. M. Lee S.-J. (1997). Regulation of skeletal muscle mass in mice by a new TGF-beta superfamily member. Nature 387, 83–90. 10.1038/387083a0 9139826

[B37] Miller D. J. Eckert J. J. Lazzari G. Duranthon-Richoux V. Sreenan J. Morris D. (2003). Tight junction messenger RNA expression levels in bovine embryos are dependent upon the ability to compact and *in vitro* culture methods. Biol. reproduction 68, 1394–1402. 10.1095/biolreprod.102.009951 12606485

[B38] Moriwaki K. Tsukita S. Furuse M. (2007). Tight junctions containing claudin 4 and 6 are essential for blastocyst formation in preimplantation mouse embryos. Dev. Biol. 312, 509–522. 10.1016/j.ydbio.2007.09.049 17980358

[B39] Moussa M. Shu J. Zhang X. Zeng F. (2014). Cryopreservation of mammalian oocytes and embryos: current problems and future perspectives. Sci. China Life Sci. 57, 903–914. 10.1007/s11427-014-4689-z 25104318

[B40] Nagase H. Woessner J. F. (1999). Matrix metalloproteinases. J. Biol. Chem. 274, 21491–21494. 10.1074/jbc.274.31.21491 10419448

[B41] Peiris H. N. Mitchell M. D. (2012). The expression and potential functions of placental myostatin. Placenta 33, 902–907. 10.1016/j.placenta.2012.06.021 22818745

[B42] Peiris H. N. Salomon C. Payton D. Ashman K. Vaswani K. Chan A. (2014). Myostatin is localized in extravillous trophoblast and up-regulates migration. J. Clin. Endocrinol. Metabolism 99, E2288–E2297. 10.1210/jc.2014-2615 25093622

[B43] Peter Holm H. C. Callesen H. (1998). *In vivo* versus *in vitro* produced bovine ova similarities and differences relevant for practical application. Reprod. Nutr. Dev. 38, 579–594. 10.1051/rnd:19980601 9932292

[B44] Qu P. Qing S. Liu R. Qin H. Wang W. Qiao F. (2017). Effects of embryo-derived exosomes on the development of bovine cloned embryos. PloS one 12, e0174535. 10.1371/journal.pone.0174535 28350875 PMC5370134

[B45] Reardon S. N. King M. L. Maclean J. A. Mann J. L. Demayo F. J. Lydon J. P. (2012). CDH1 is essential for endometrial differentiation, gland development, and adult function in the mouse uterus. Biol. reproduction 86 (141), 1–10. 10.1095/biolreprod.112.098871 PMC3364924 22378759

[B46] Ribeiro J. C. Carrageta D. F. Bernardino R. L. Alves M. G. Oliveira P. F. (2022). Aquaporins and animal gamete cryopreservation: advances and future challenges. Animals 12, 359. 10.3390/ani12030359 35158682 PMC8833750

[B47] Robinson D. H. Benos D. J. (1991). Chapter 4 ion and solute transport in preimplantation mammalian embryos. Curr. Top. Membr. 39, 121–150. 10.1016/S0070-2161(08)60802-3

[B48] Sakatani M. Yamanaka K. Kobayashi S. Takahashi M. (2008). Heat shock-derived reactive oxygen species induce embryonic mortality in *in vitro* early stage bovine embryos. J. Reproduction Dev. 54, 496–501. 10.1262/jrd.20017 18762719

[B49] Schneyer A. Sidis Y. Xia Y. Saito S. Del Re E. Lin H. Y. (2004). Differential actions of follistatin and follistatin-like 3. Mol. Cell. Endocrinol. 225, 25–28. 10.1016/j.mce.2004.02.009 15451564

[B50] Sidrat T. Khan A. A. Joo M. D. Xu L. El-Sheikh M. Ko J. H. (2022). Extracellular vesicles improve embryo cryotolerance by maintaining the tight junction integrity during blastocoel re-expansion. Reproduction 163, 219–232. 10.1530/REP-21-0320 35129460 PMC8942337

[B51] Strumpf D. Mao C.-A. Yamanaka Y. Ralston A. Chawengsaksophak K. Beck F. (2005). Cdx2 is required for correct cell fate specification and differentiation of trophectoderm in the mouse blastocyst. Development 132, 2093–2102. 10.1242/dev.01801 15788452

[B52] Thibier M. (2005). The zootechnical applications of biotechnology in animal reproduction: current methods and perspectives. Reprod. Nutr. Dev. 45, 235–242. 10.1051/rnd:2005016 15982450

[B53] Thompson J. G. (1997). Comparison between *in vivo*-derived and *in vitro*-produced pre-elongation embryos from domestic ruminants. Reproduction, Fertil. Dev. 9, 341–354. 10.1071/r96079 9261882

[B54] Tsukita S. Furuse M. Itoh M. (2001). Multifunctional strands in tight junctions. Nat. Rev. Mol. Cell Biol. 2, 285–293. 10.1038/35067088 11283726

[B55] Underhill K. Downey B. Mcfarlane C. King W. (1991). Cytogenetic analysis of Day-4 embryos from PMSG/hCG-treated prepuberal gilts. Theriogenology 35, 779–784. 10.1016/0093-691x(91)90419-e 16726947

[B56] Wheatley S. C. Isacke C. M. Crossley P. H. (1993). Restricted expression of the hyaluronan receptor, CD44, during postimplantation mouse embryogenesis suggests key roles in tissue formation and patterning. Development 119, 295–306. 10.1242/dev.119.2.295 7507029

[B57] Wilcox E. R. Burton Q. L. Naz S. Riazuddin S. Smith T. N. Ploplis B. (2001). Mutations in the gene encoding tight junction claudin-14 cause autosomal recessive deafness DFNB29. Cell 104, 165–172. 10.1016/s0092-8674(01)00200-8 11163249

[B58] Wong C. L. Huang Y. Y. Ho W. K. Poon H. K. Cheung P. L. O W. S. (2009). Growth-differentiation factor-8 (GDF-8) in the uterus: its identification and functional significance in the golden hamster. Reprod. Biol. Endocrinol. 7, 134. 10.1186/1477-7827-7-134 19930721 PMC2790456

[B59] Wooldridge L. K. Ealy A. D. (2019). Interleukin-6 increases inner cell mass numbers in bovine embryos. BMC Dev. Biol. 19, 2–11. 10.1186/s12861-019-0182-z 30709330 PMC6359871

[B60] Wu G. Gentile L. Fuchikami T. Sutter J. Psathaki K. Esteves T. C. (2010). Initiation of trophectoderm lineage specification in mouse embryos is independent of Cdx2. Development 137, 4159–4169. 10.1242/dev.056630 21098565 PMC2990207

[B61] Xie J. Zhu H. Chang H. M. Klausen C. Dong M. Leung P. C. K. (2020). GDF8 promotes the cell invasiveness in human trophoblasts by upregulating the expression of follistatin-like 3 through the ALK5-SMAD2/3 signaling pathway. Front. Cell Dev. Biol. 8, 573781. 10.3389/fcell.2020.573781 33195207 PMC7655915

[B62] Yoon J. D. Hwang S. U. Kim M. Lee G. Jeon Y. Hyun S. H. (2019). GDF8 enhances SOX2 expression and blastocyst total cell number in porcine IVF embryo development. Theriogenology 129, 70–76. 10.1016/j.theriogenology.2019.02.007 30825707

[B63] Zhang S. Mesalam A. Joo M. D. Lee K. L. Hwang J. Y. Xu L. (2020). Matrix metalloproteinases improves trophoblast invasion and pregnancy potential in mice. Theriogenology 151, 144–150. 10.1016/j.theriogenology.2020.02.002 32344273

[B64] Zheng X. Zheng Y. Qin D. Yao Y. Zhang X. Zhao Y. (2022). Regulatory role and potential importance of GDF-8 in ovarian reproductive activity. Front. Endocrinol. (Lausanne) 13, 878069. 10.3389/fendo.2022.878069 35692411 PMC9178251

