# Supplementary figures and images for: GDF-8 improves in vitro implantation and cryo-tolerance by stimulating the ALK5-SMAD2/3 signaling in bovine IVF embryo development
Source: Front Cell Dev Biol. 2024 Mar 21;12:1345669. doi: 10.3389/fcell.2024.1345669 (PMC10991729; doi:10.3389/fcell.2024.1345669)

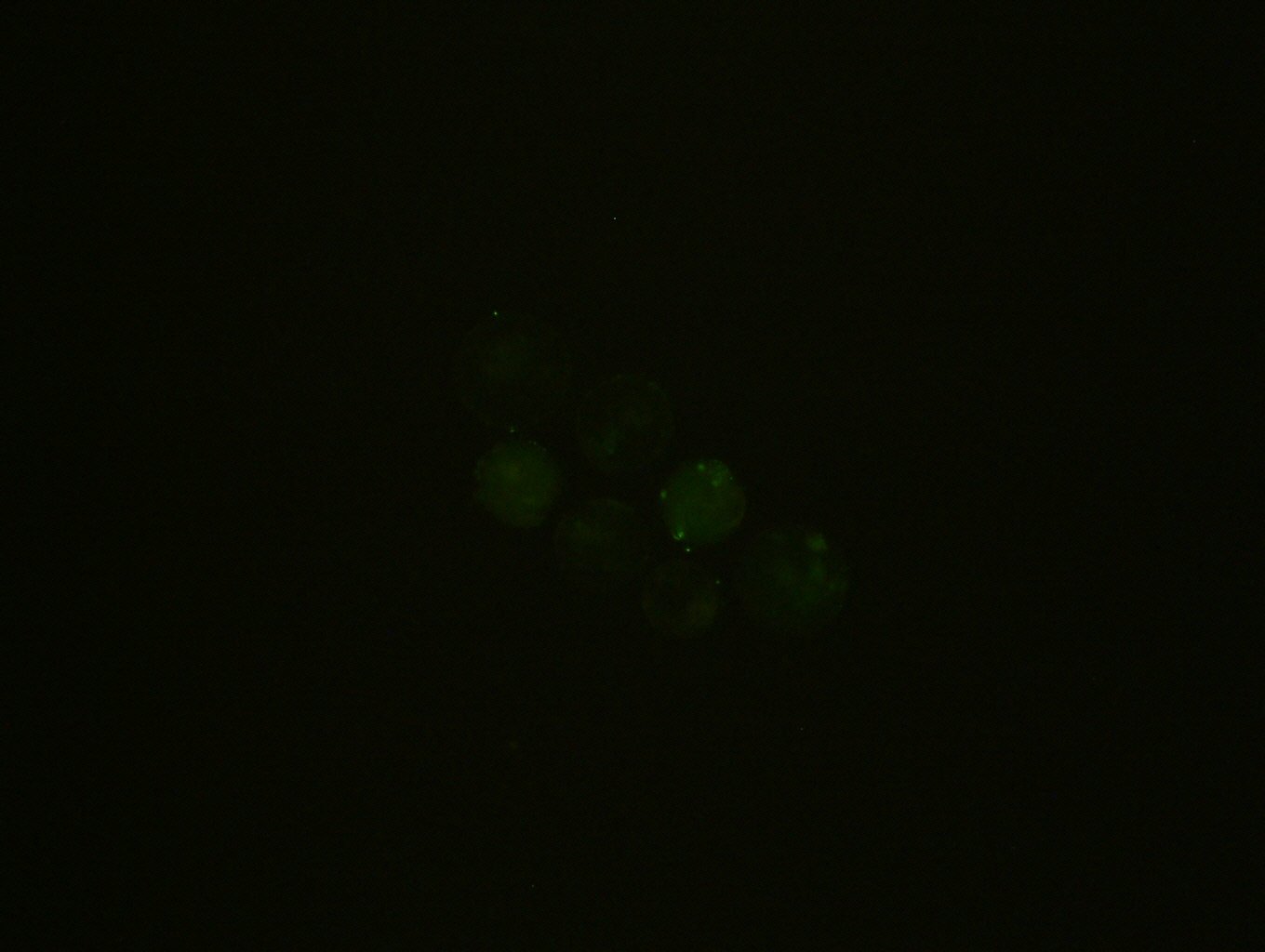

Supplement: Supplementary file 2 [file DataSheet3.ZIP › Raw data for figure 3/Control dextran x 100.jpg]

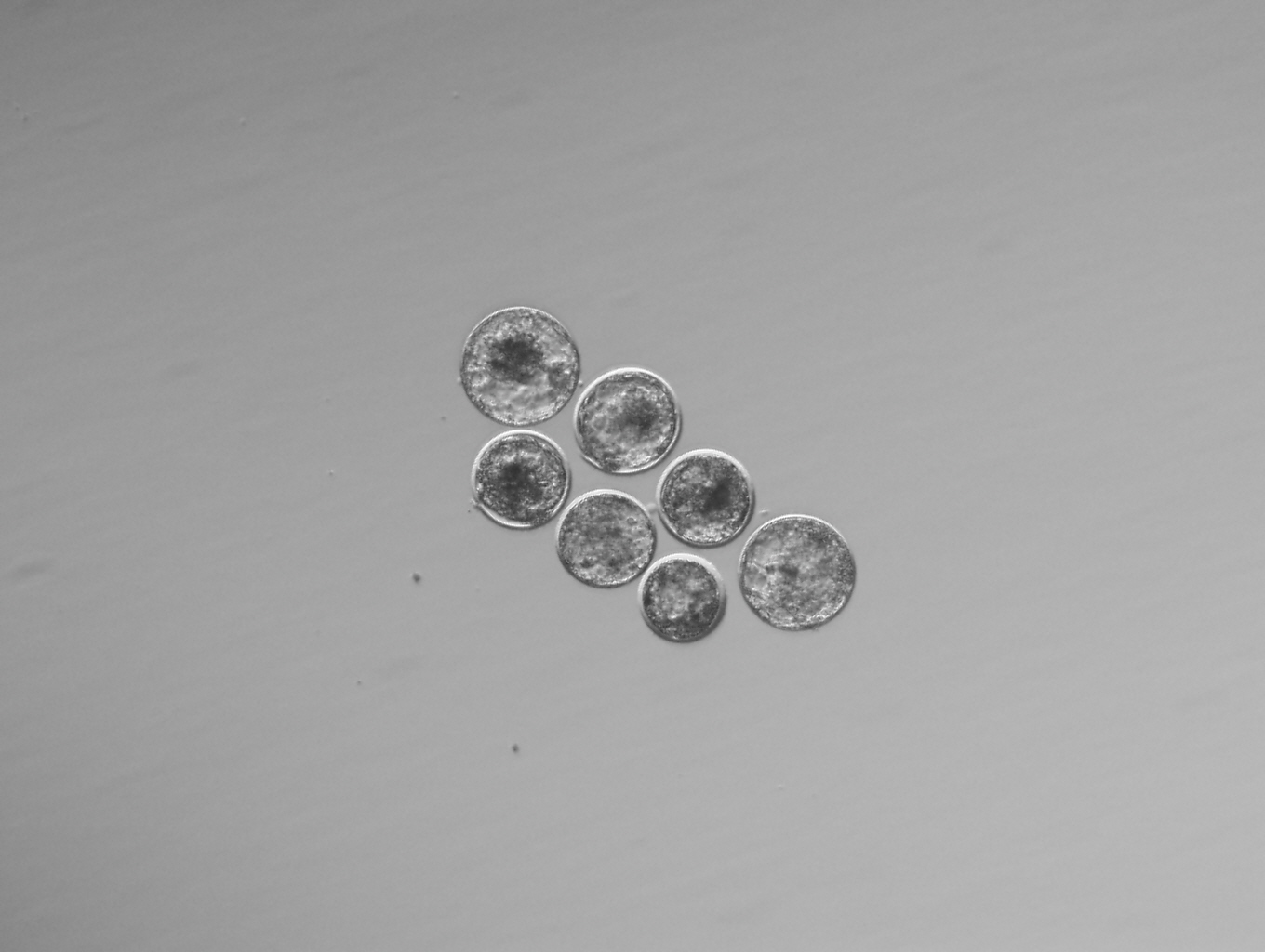

Supplement: Supplementary file 2 [file DataSheet3.ZIP › Raw data for figure 3/Control Thawed-24h x 100.jpg]

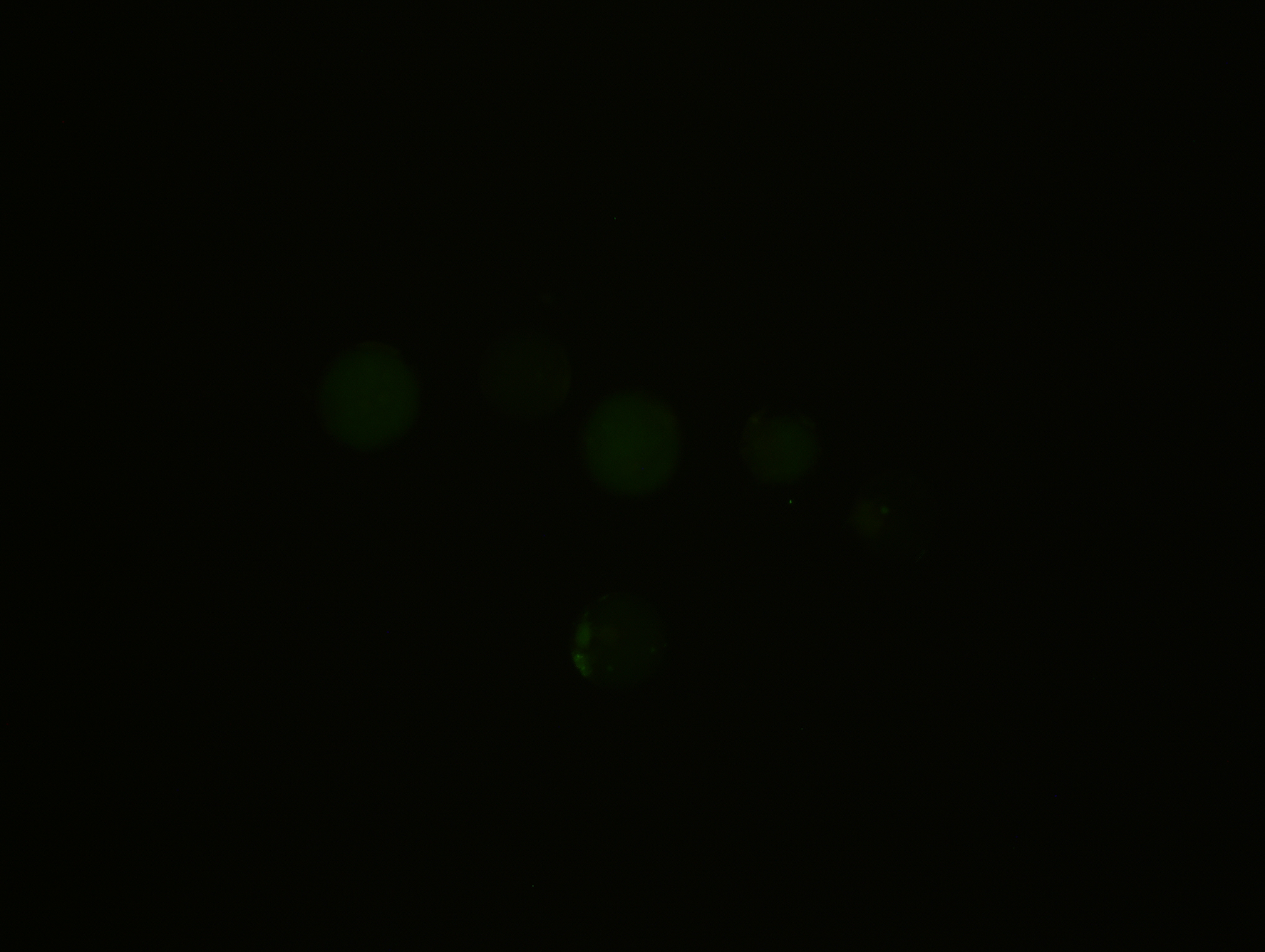

Supplement: Supplementary file 2 [file DataSheet3.ZIP › Raw data for figure 3/GDF-8 dextran x 100.jpg]

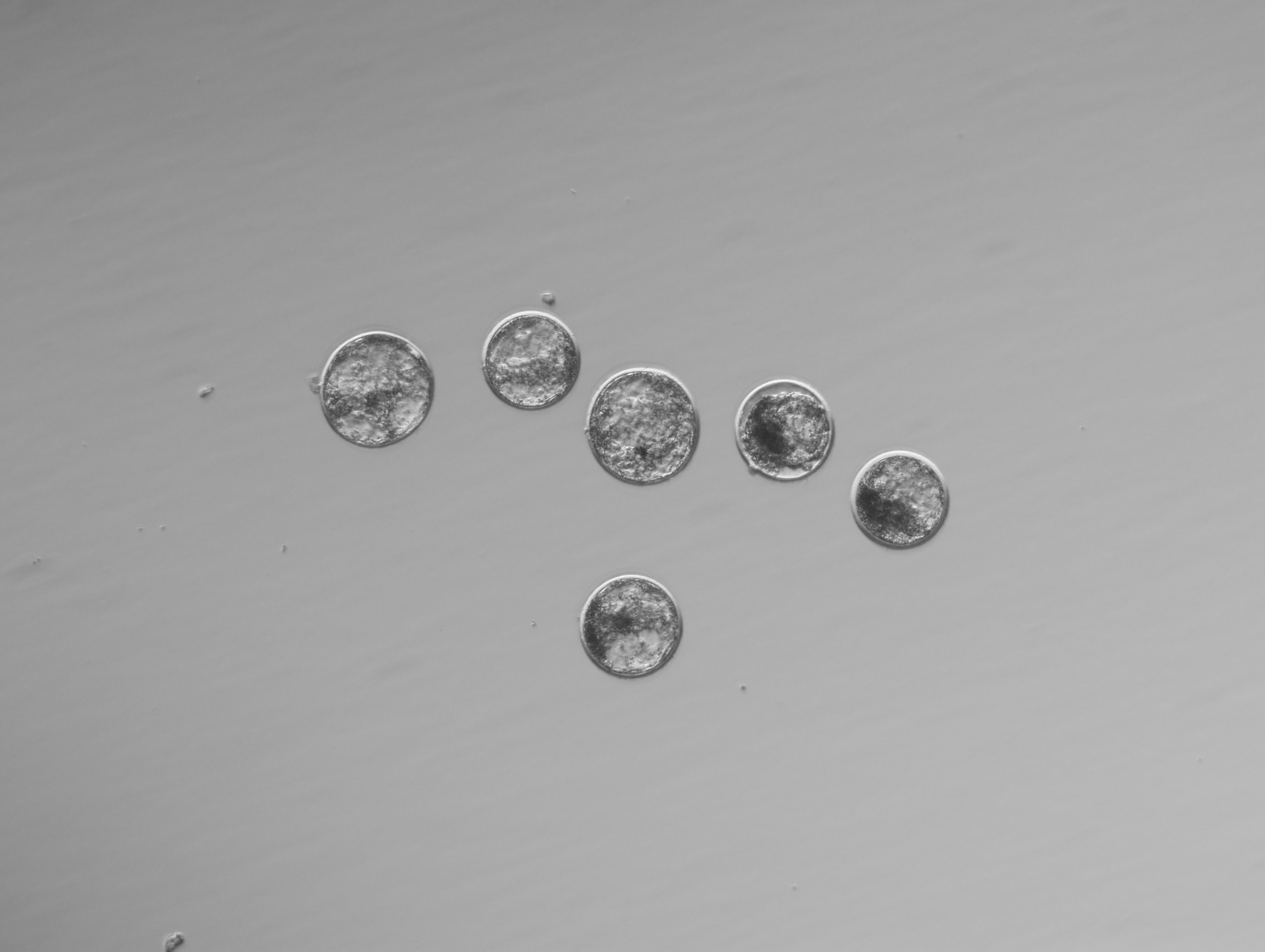

Supplement: Supplementary file 2 [file DataSheet3.ZIP › Raw data for figure 3/GDF-8 Thawed-24h x 100.jpg]

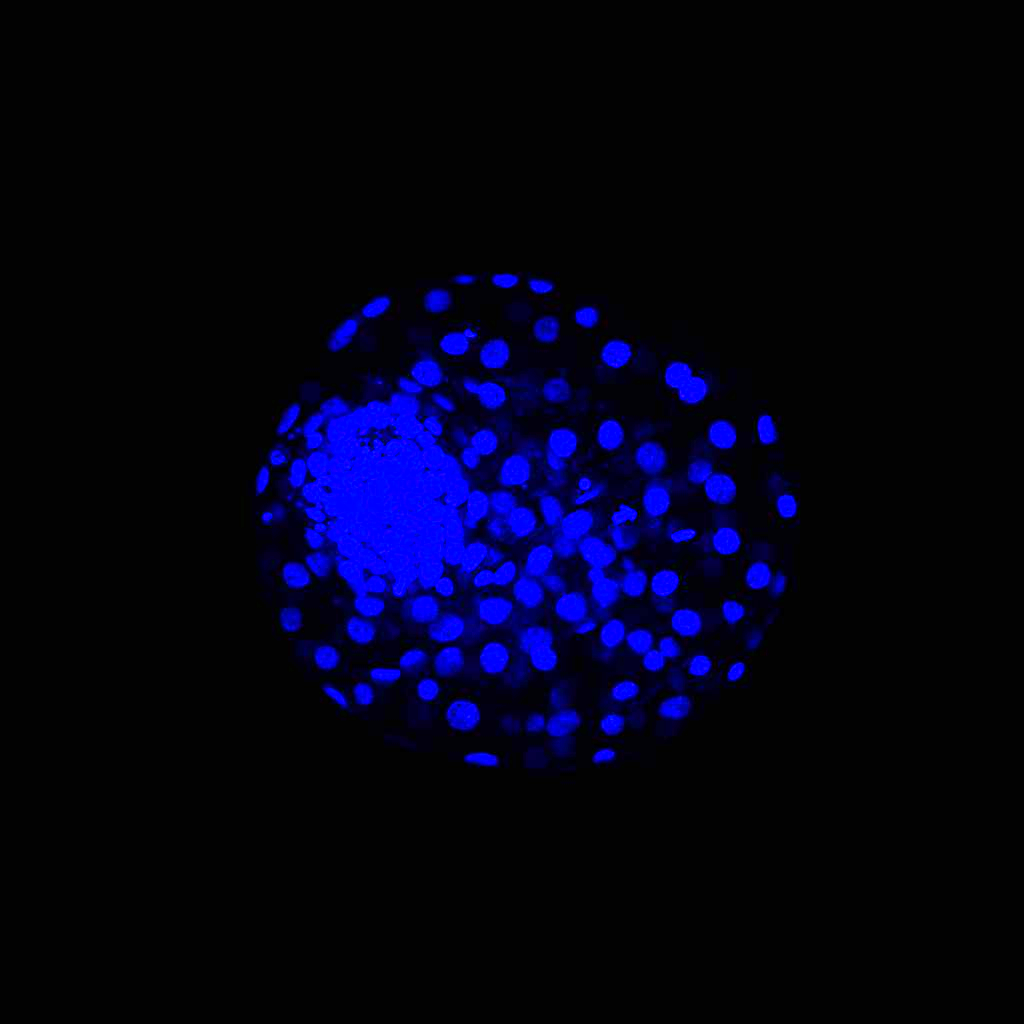

Supplement: Supplementary file 3 [file DataSheet4.ZIP › Raw data for figure 4/Control CD44 DAPI x 200.tif]

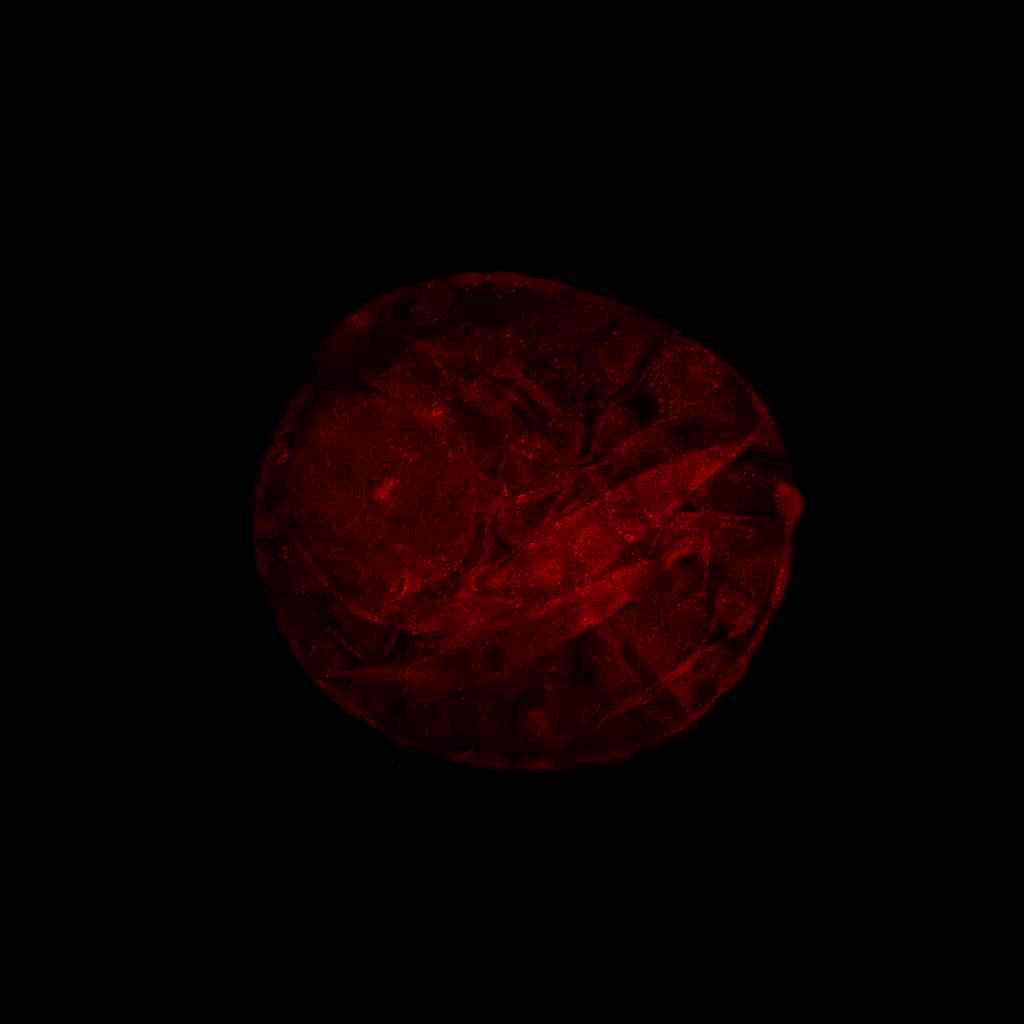

Supplement: Supplementary file 3 [file DataSheet4.ZIP › Raw data for figure 4/Control CD44 x 200.tif]

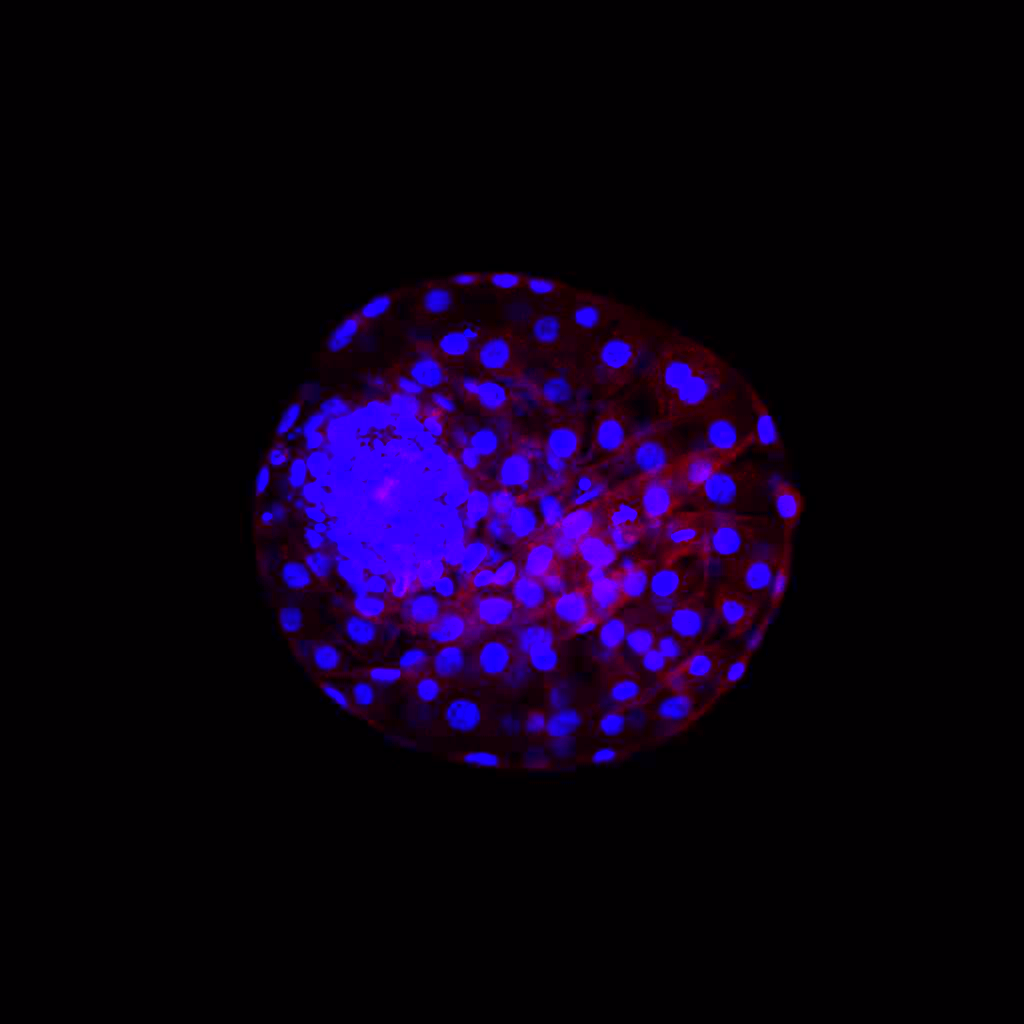

Supplement: Supplementary file 3 [file DataSheet4.ZIP › Raw data for figure 4/Control merge.tif]

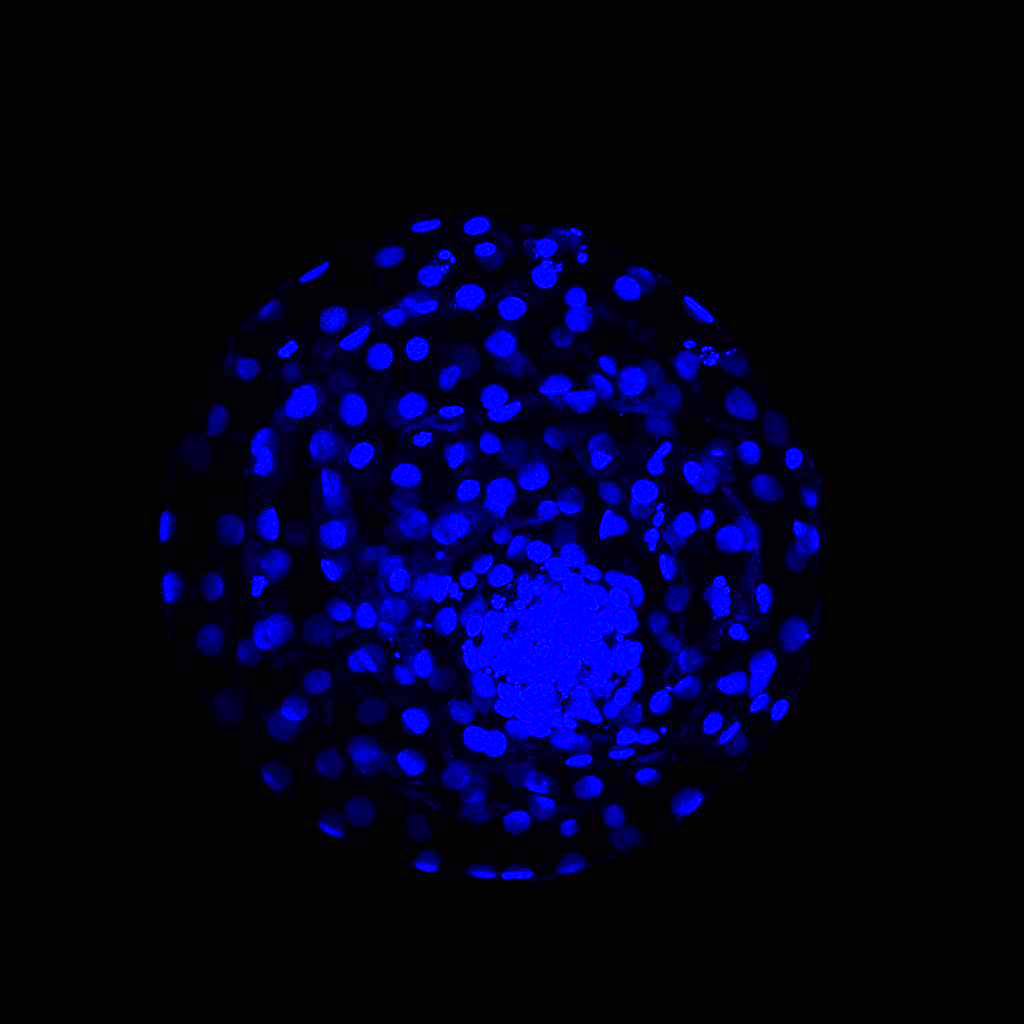

Supplement: Supplementary file 3 [file DataSheet4.ZIP › Raw data for figure 4/GDF-8 CD44 DAPI x 200.tif]

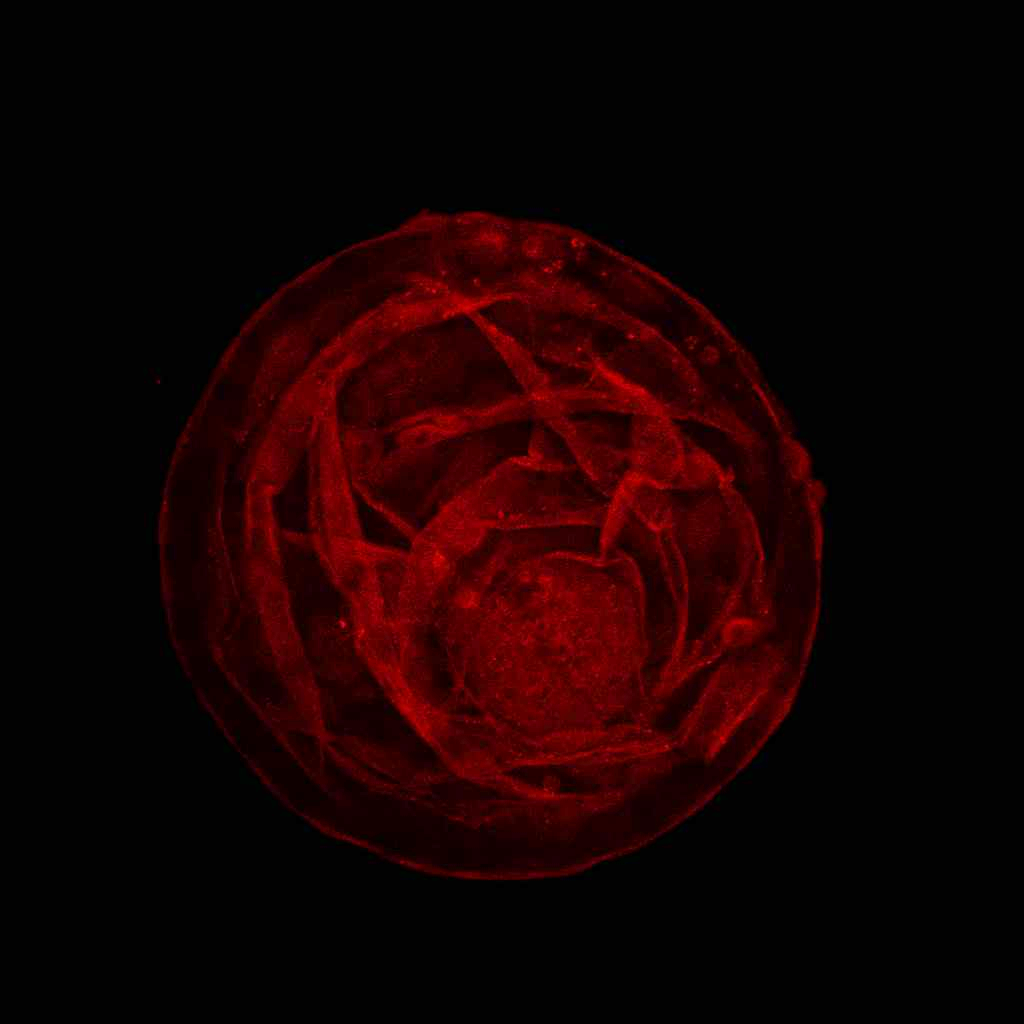

Supplement: Supplementary file 3 [file DataSheet4.ZIP › Raw data for figure 4/GDF-8 CD44 x 200.tif]

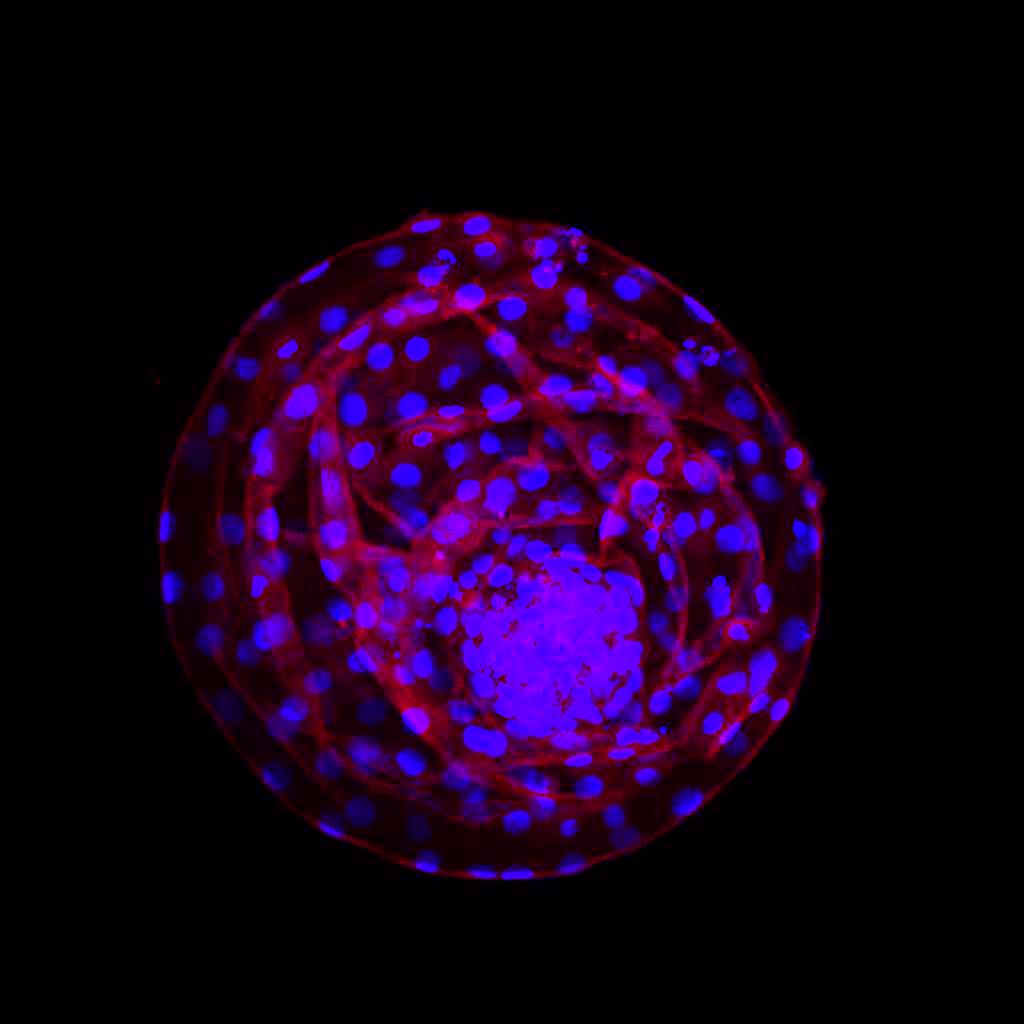

Supplement: Supplementary file 3 [file DataSheet4.ZIP › Raw data for figure 4/GDF-8 merge.tif]

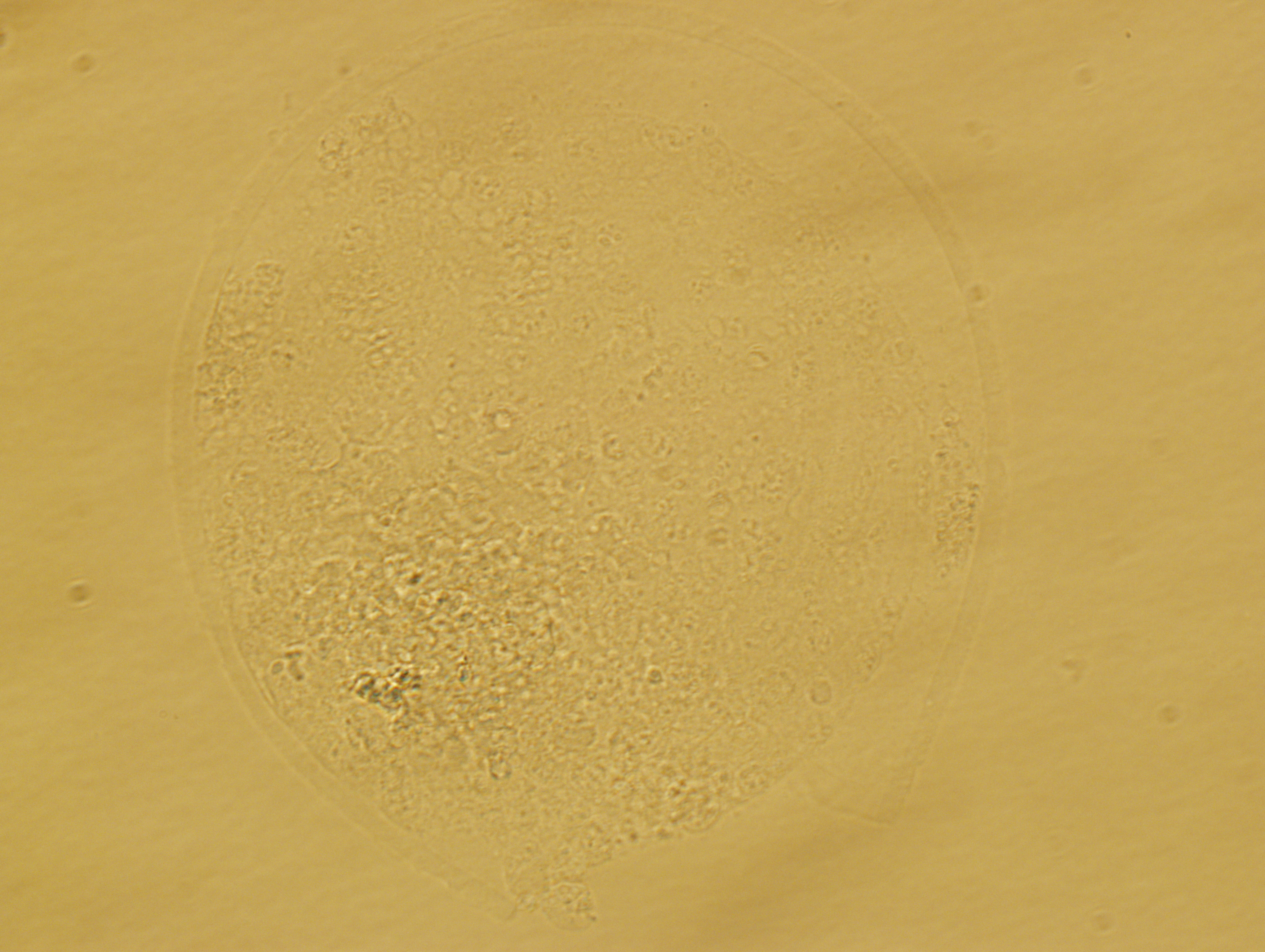

Supplement: Supplementary file 4 [file DataSheet1.ZIP › Raw data for figure 1/Control B.F x 200.jpg]

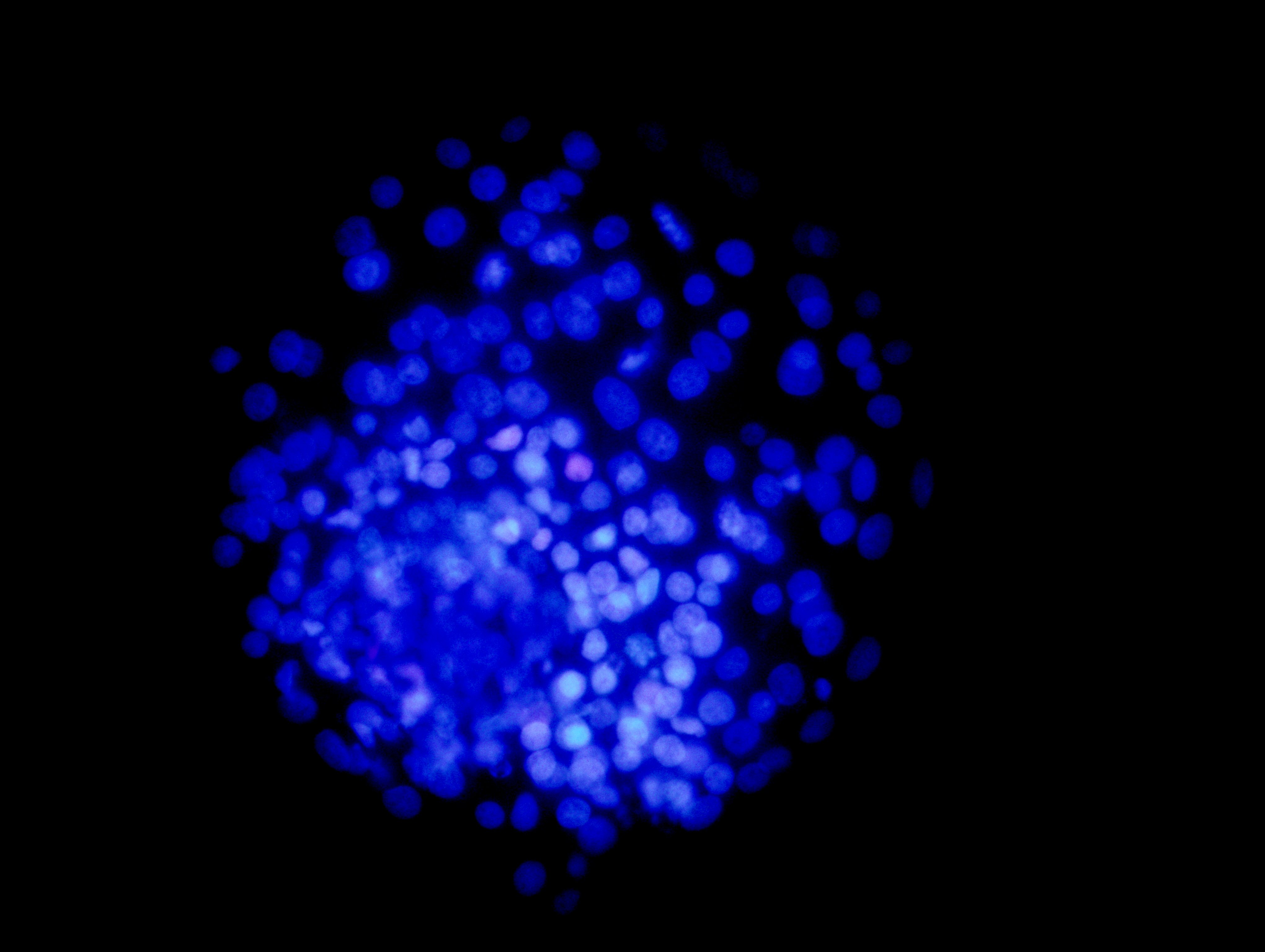

Supplement: Supplementary file 4 [file DataSheet1.ZIP › Raw data for figure 1/Control Hoechst33342 x 200.jpg]

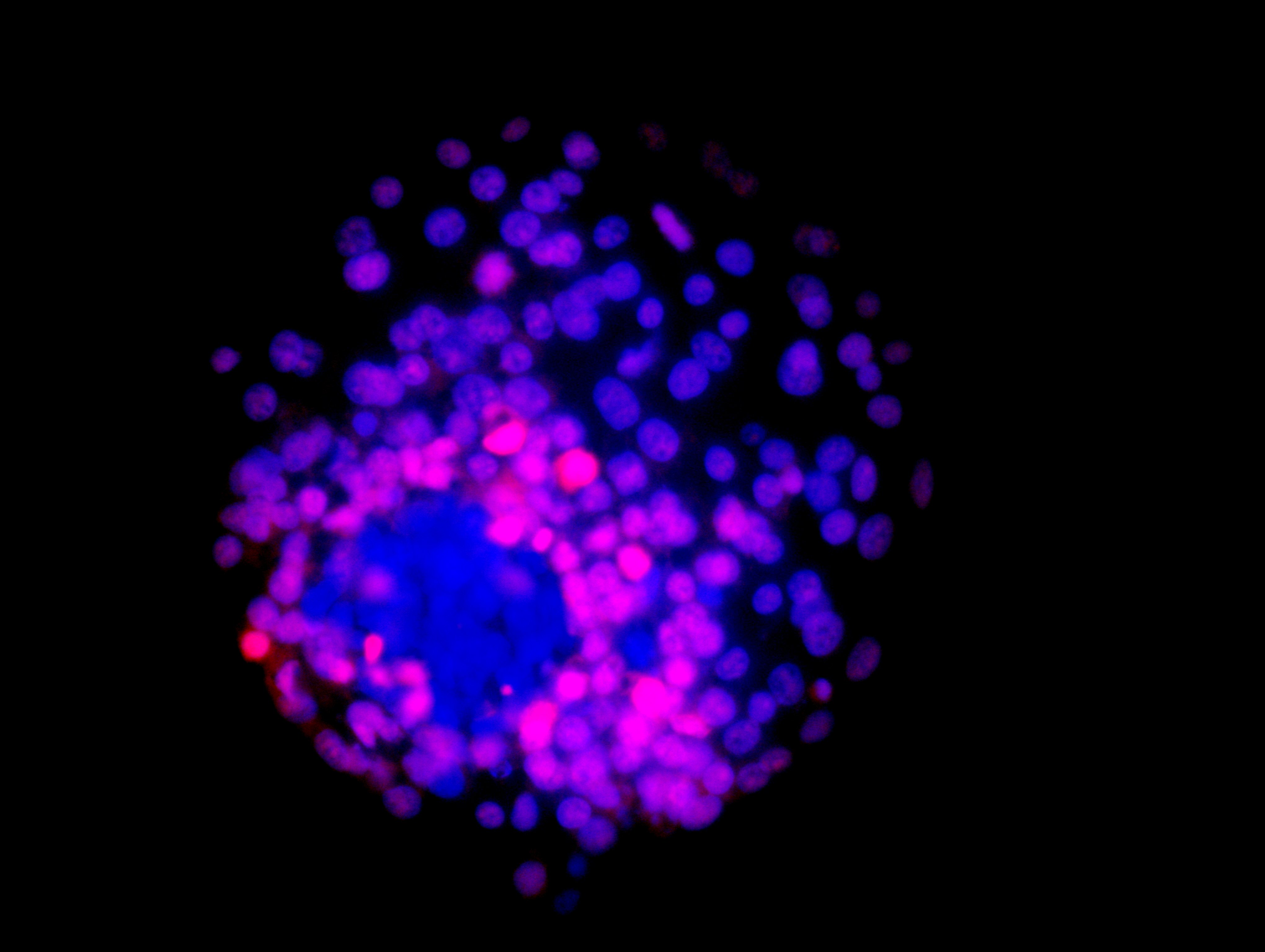

Supplement: Supplementary file 4 [file DataSheet1.ZIP › Raw data for figure 1/Control Merge x 200.jpg]

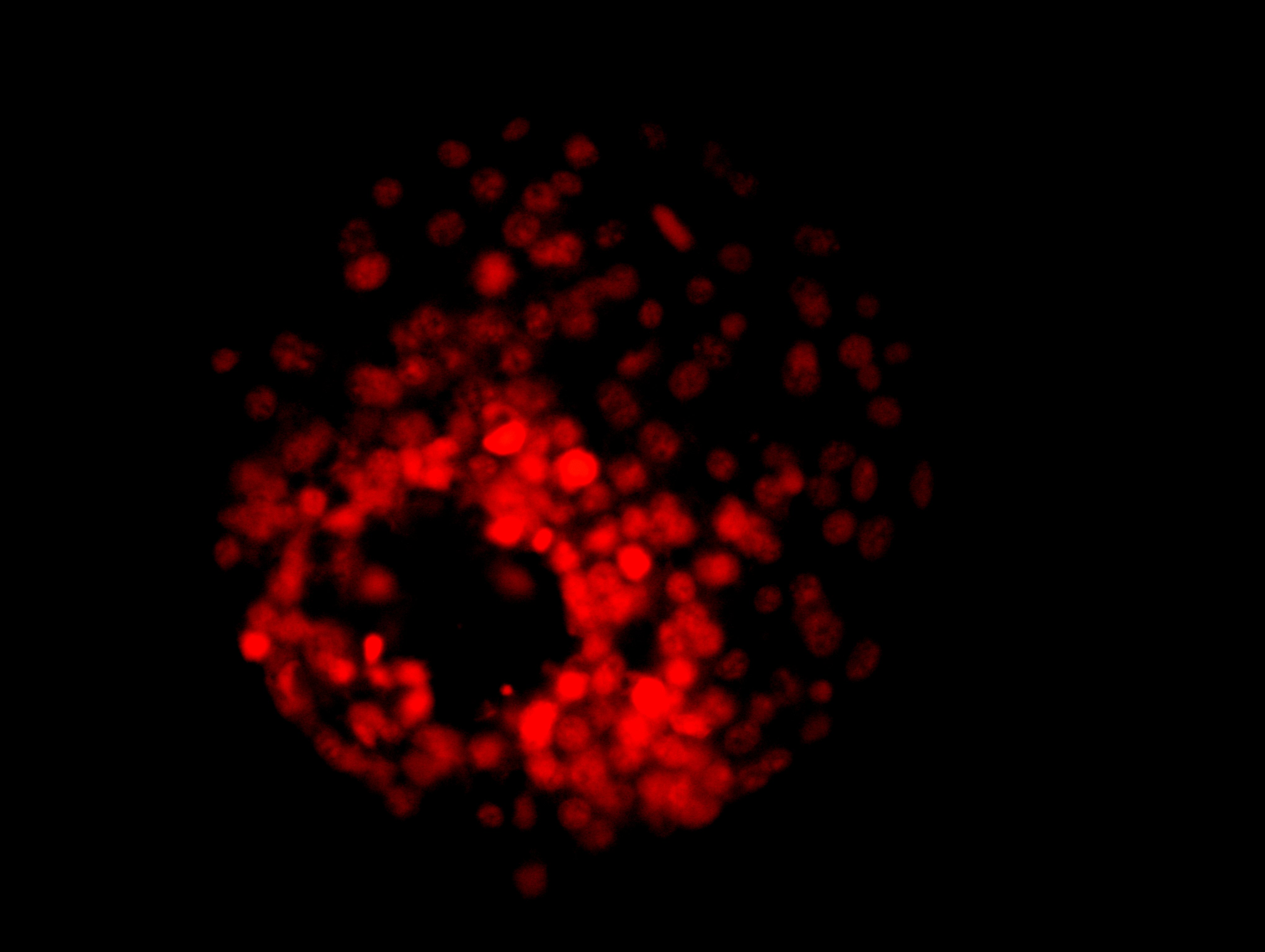

Supplement: Supplementary file 4 [file DataSheet1.ZIP › Raw data for figure 1/Control propidium iodide x 200.jpg]

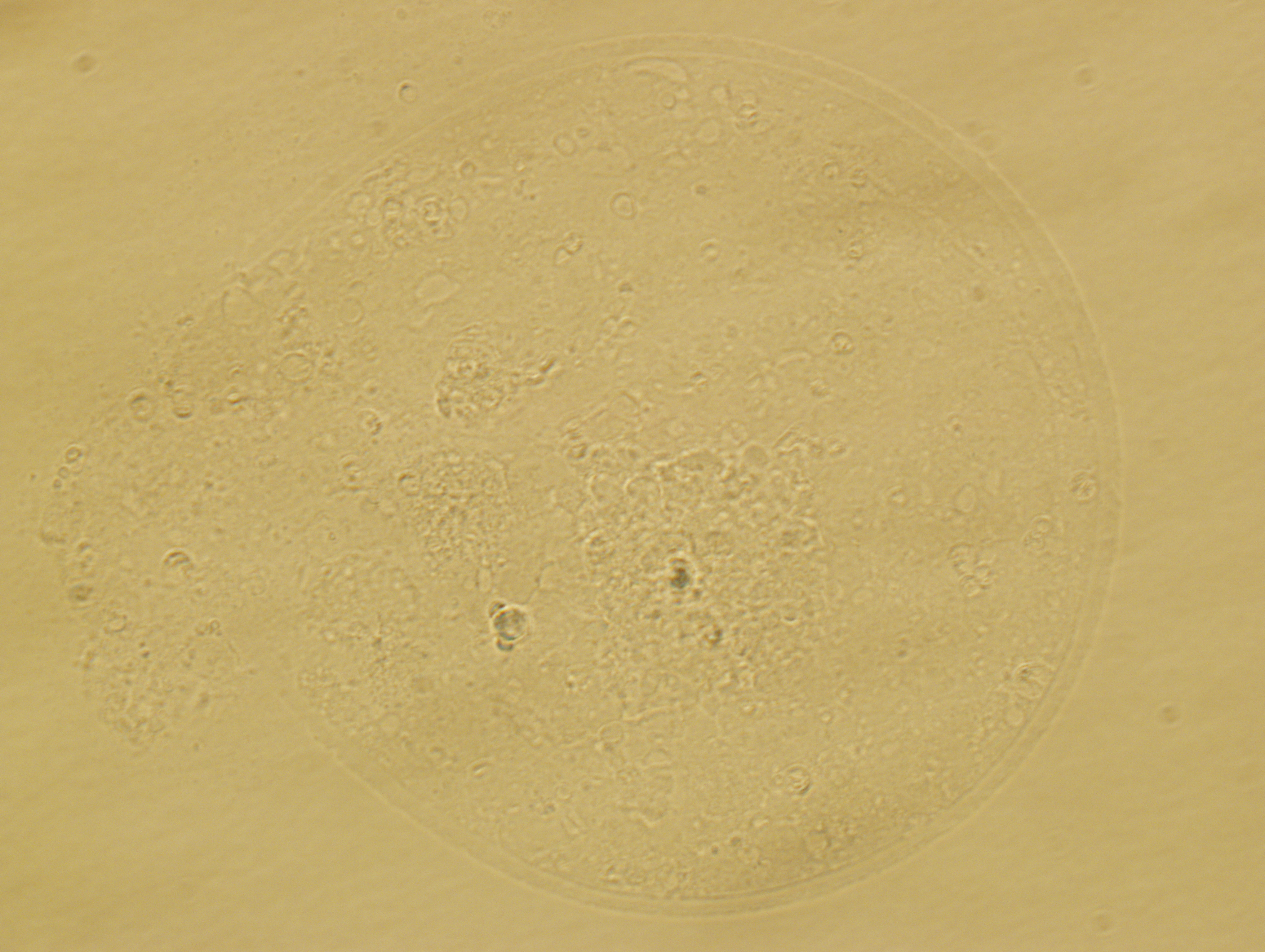

Supplement: Supplementary file 4 [file DataSheet1.ZIP › Raw data for figure 1/GDF-8 B.F x 200.jpg]

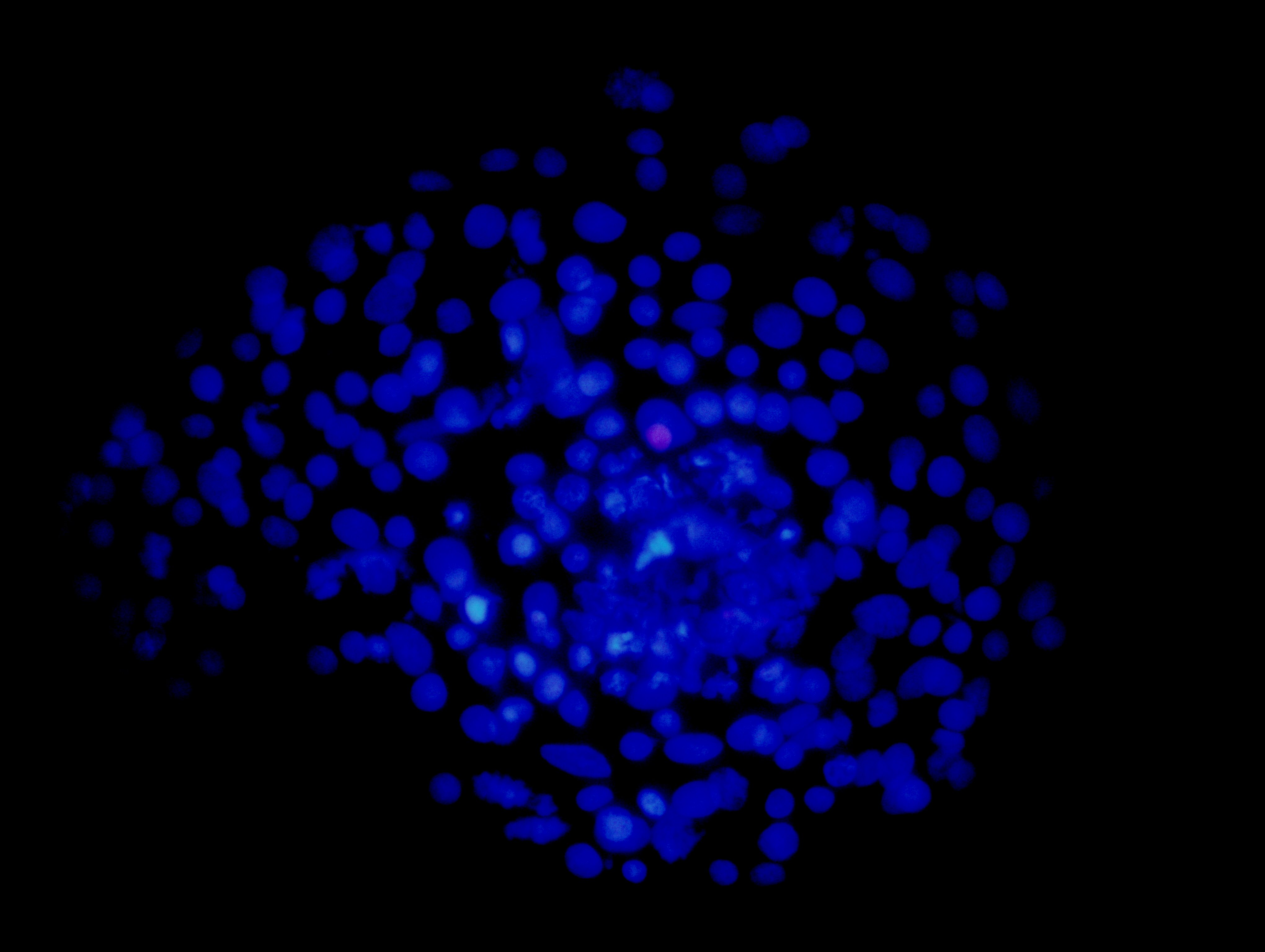

Supplement: Supplementary file 4 [file DataSheet1.ZIP › Raw data for figure 1/GDF-8 Hoechst33342 x 200.jpg]

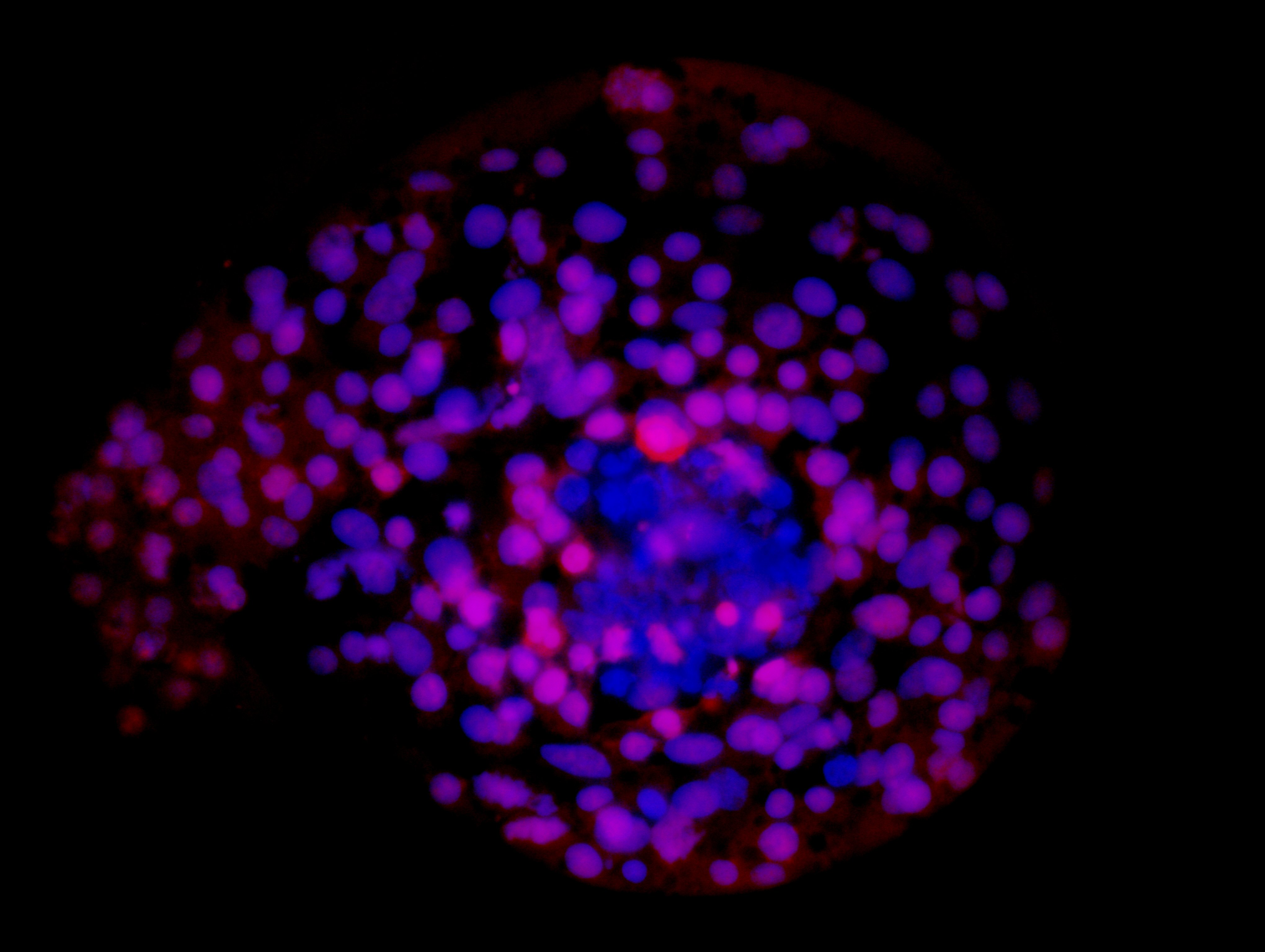

Supplement: Supplementary file 4 [file DataSheet1.ZIP › Raw data for figure 1/GDF-8 Merge x 200.jpg]

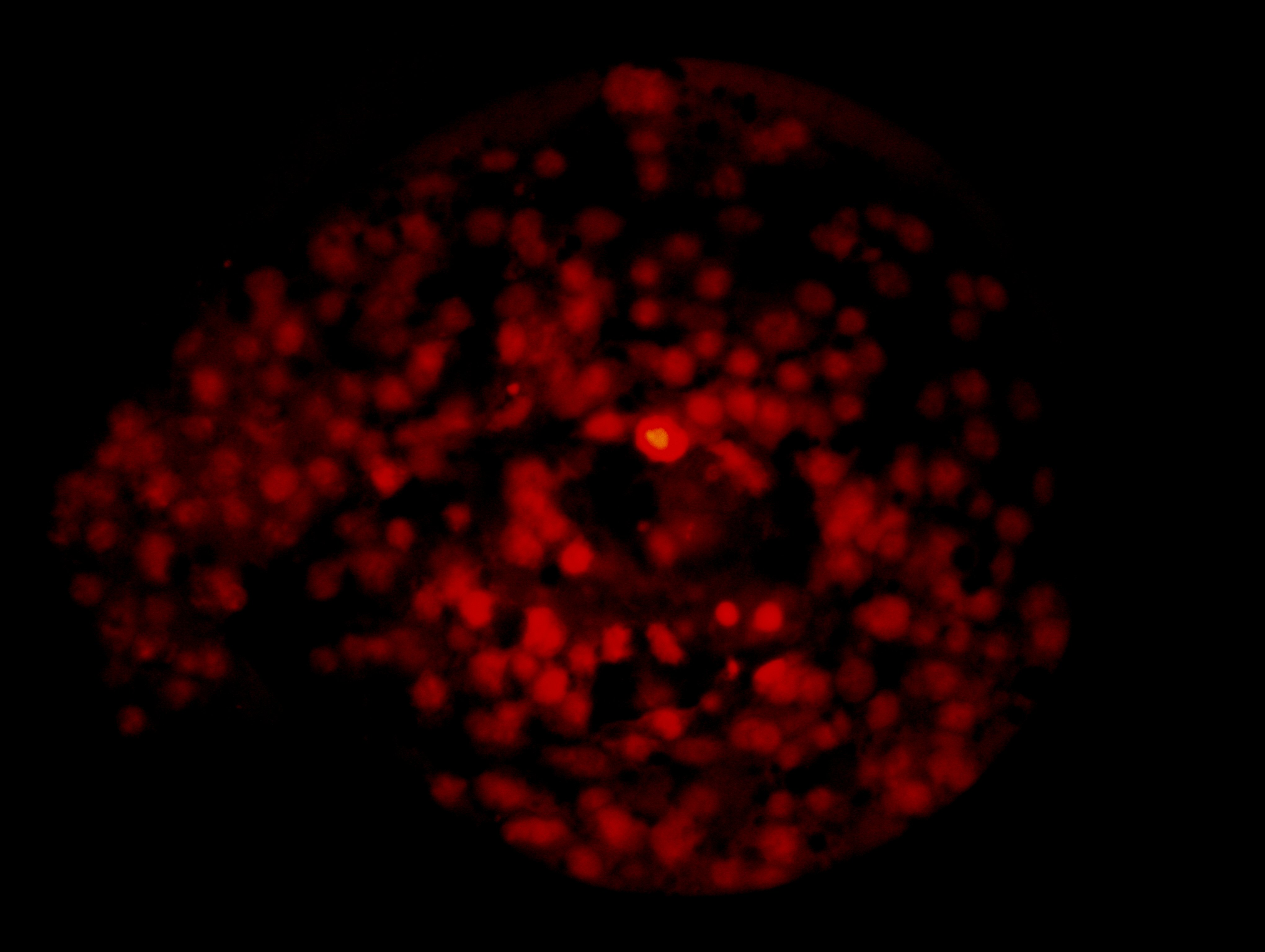

Supplement: Supplementary file 4 [file DataSheet1.ZIP › Raw data for figure 1/GDF-8 propidium iodide x 200.jpg]

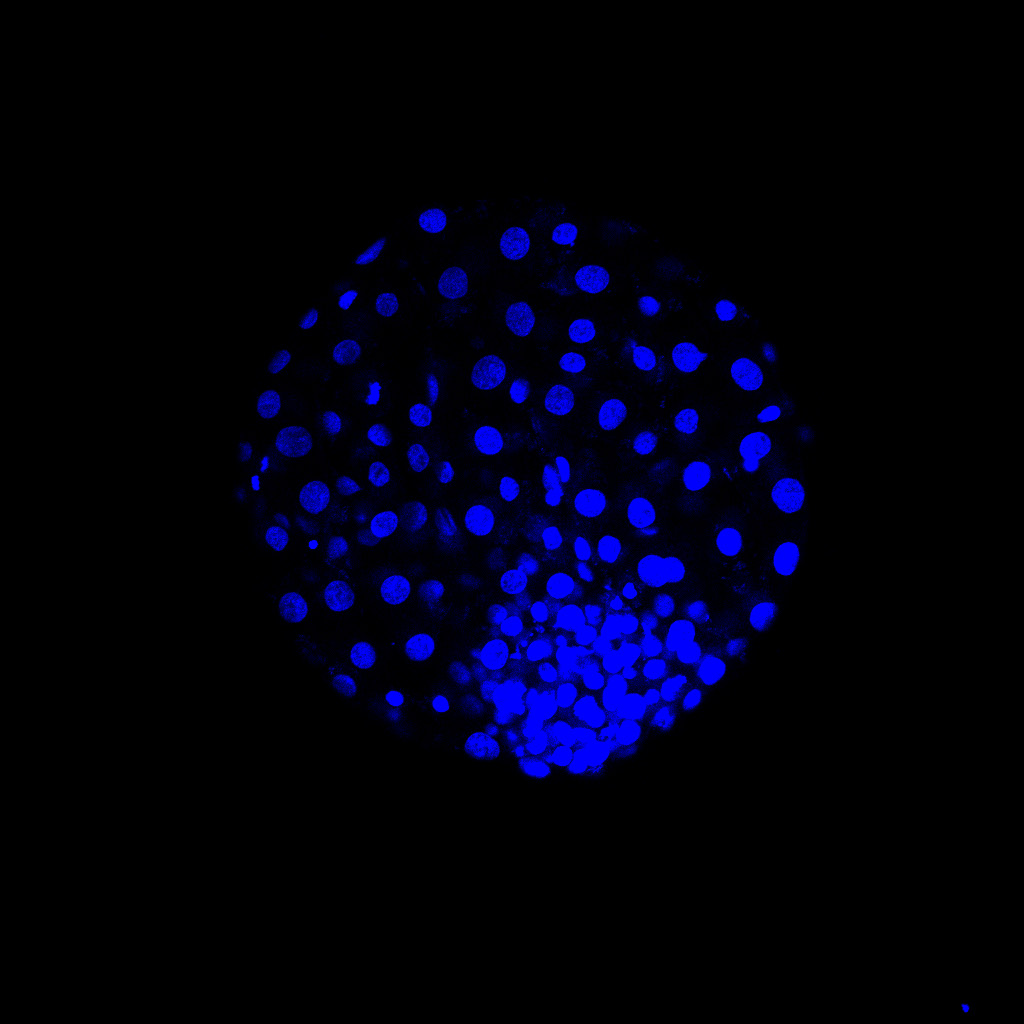

Supplement: Supplementary file 5 [file DataSheet6.ZIP › Raw data for figure 6/Control AQP3 DAPI x 200.tif]

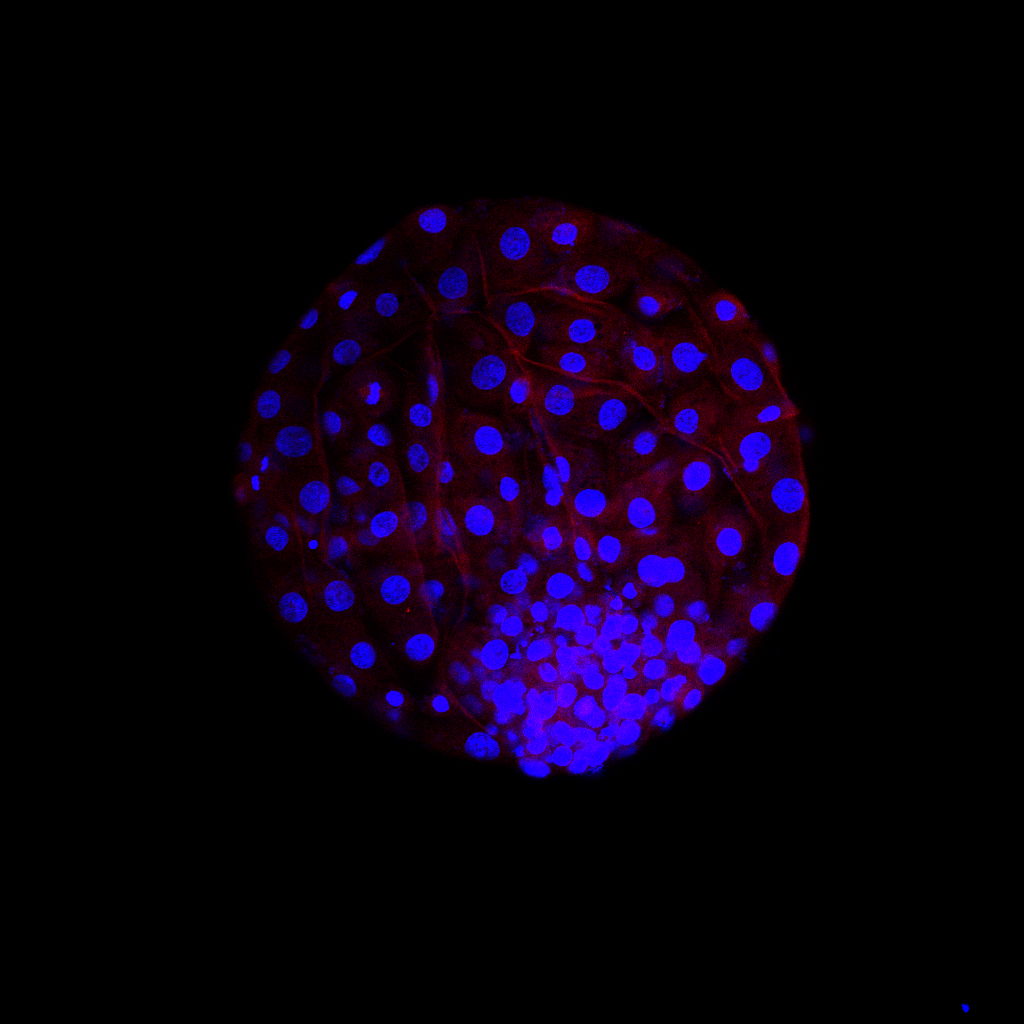

Supplement: Supplementary file 5 [file DataSheet6.ZIP › Raw data for figure 6/Control AQP3 Merge x 200.tif]

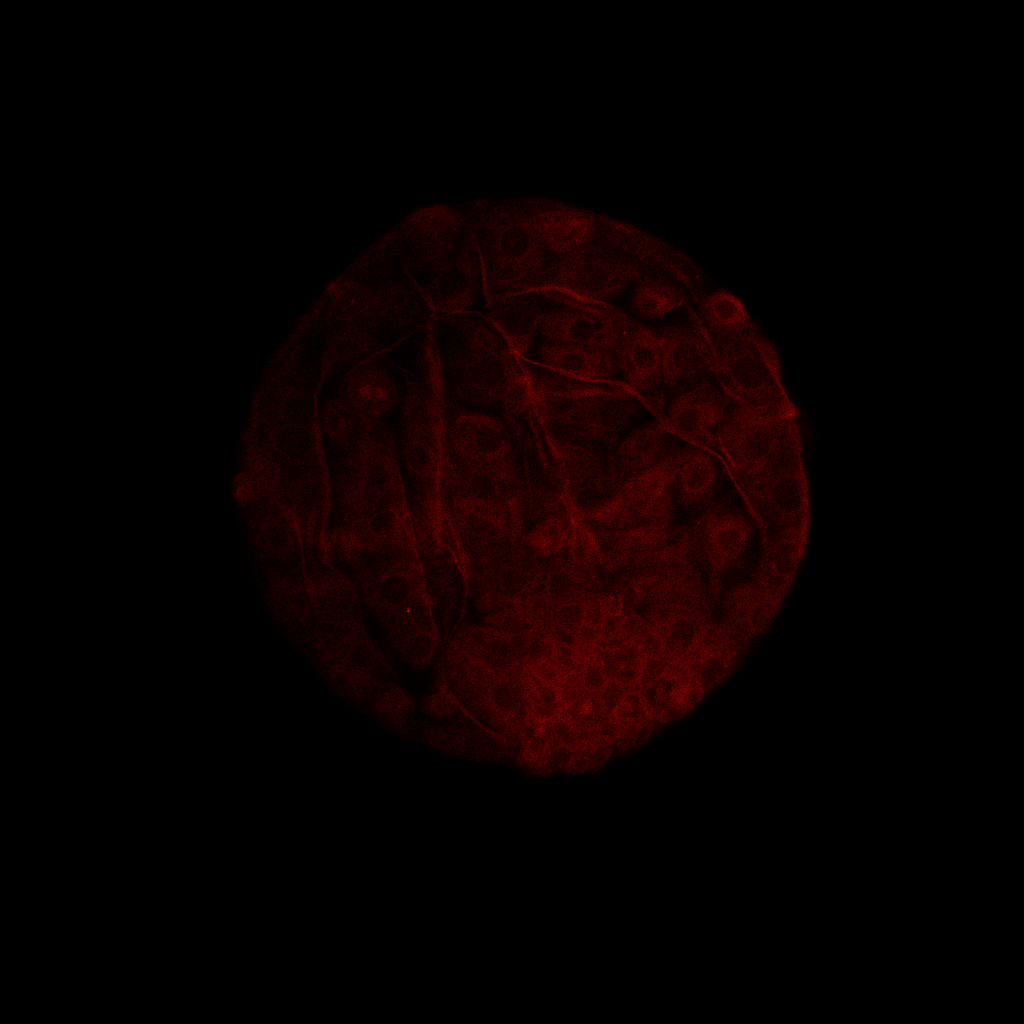

Supplement: Supplementary file 5 [file DataSheet6.ZIP › Raw data for figure 6/Control AQP3 x 200.tif]

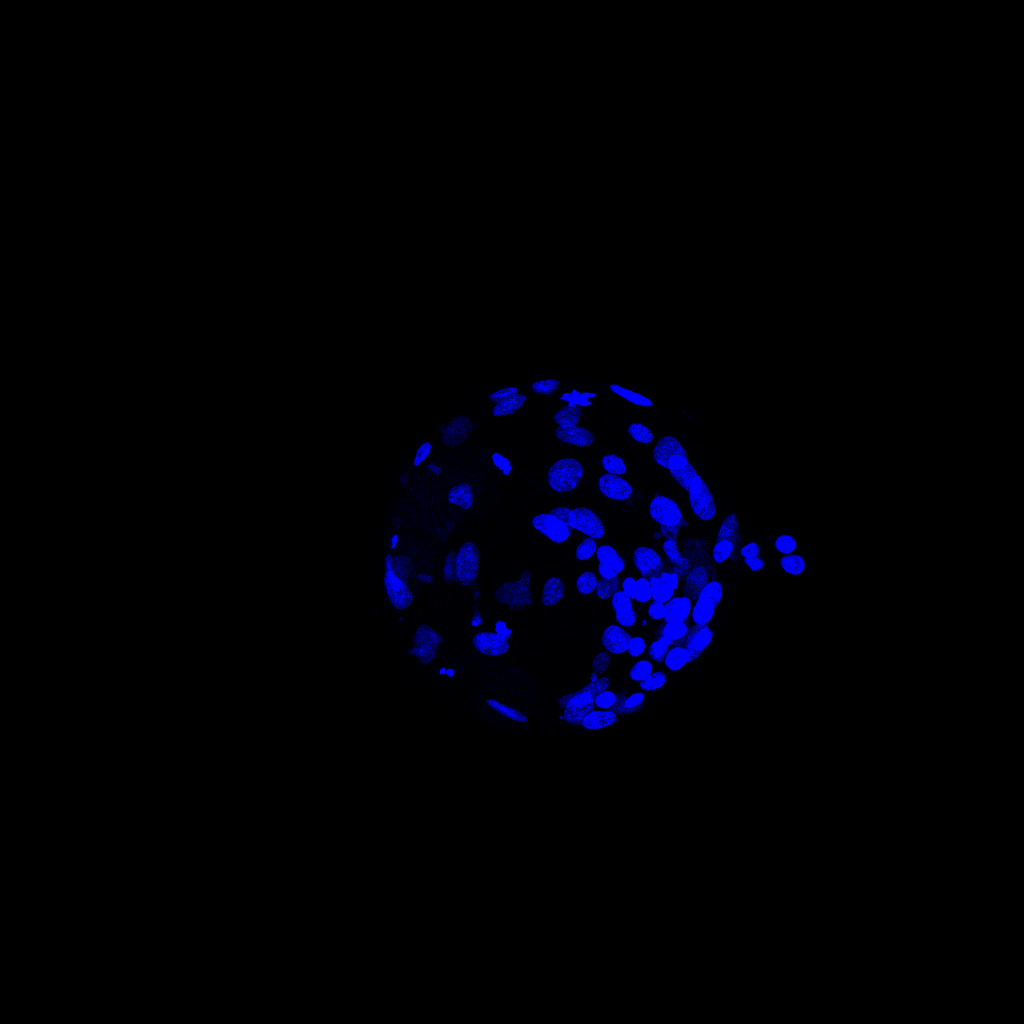

Supplement: Supplementary file 5 [file DataSheet6.ZIP › Raw data for figure 6/Control ATP1a1 DAPI x 200.tif]

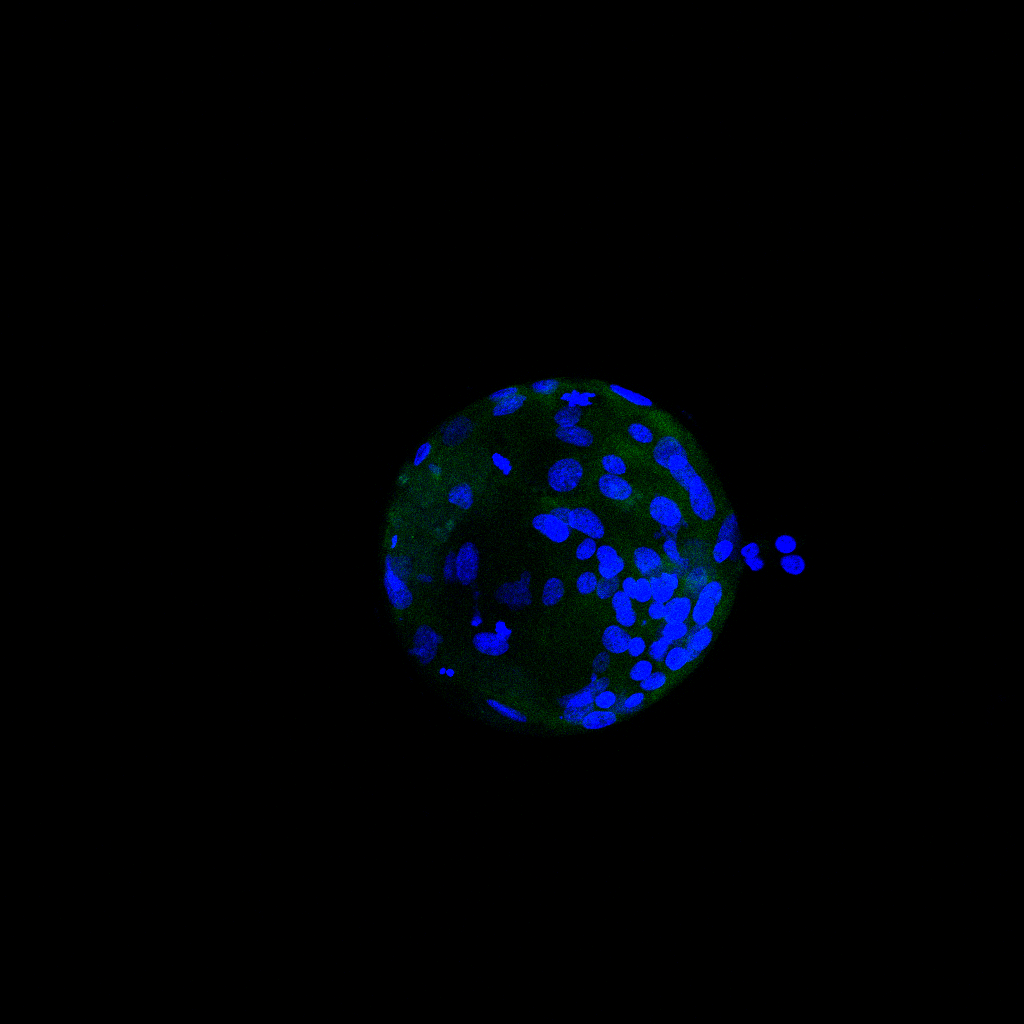

Supplement: Supplementary file 5 [file DataSheet6.ZIP › Raw data for figure 6/Control ATP1a1 Merge x 200.tif]

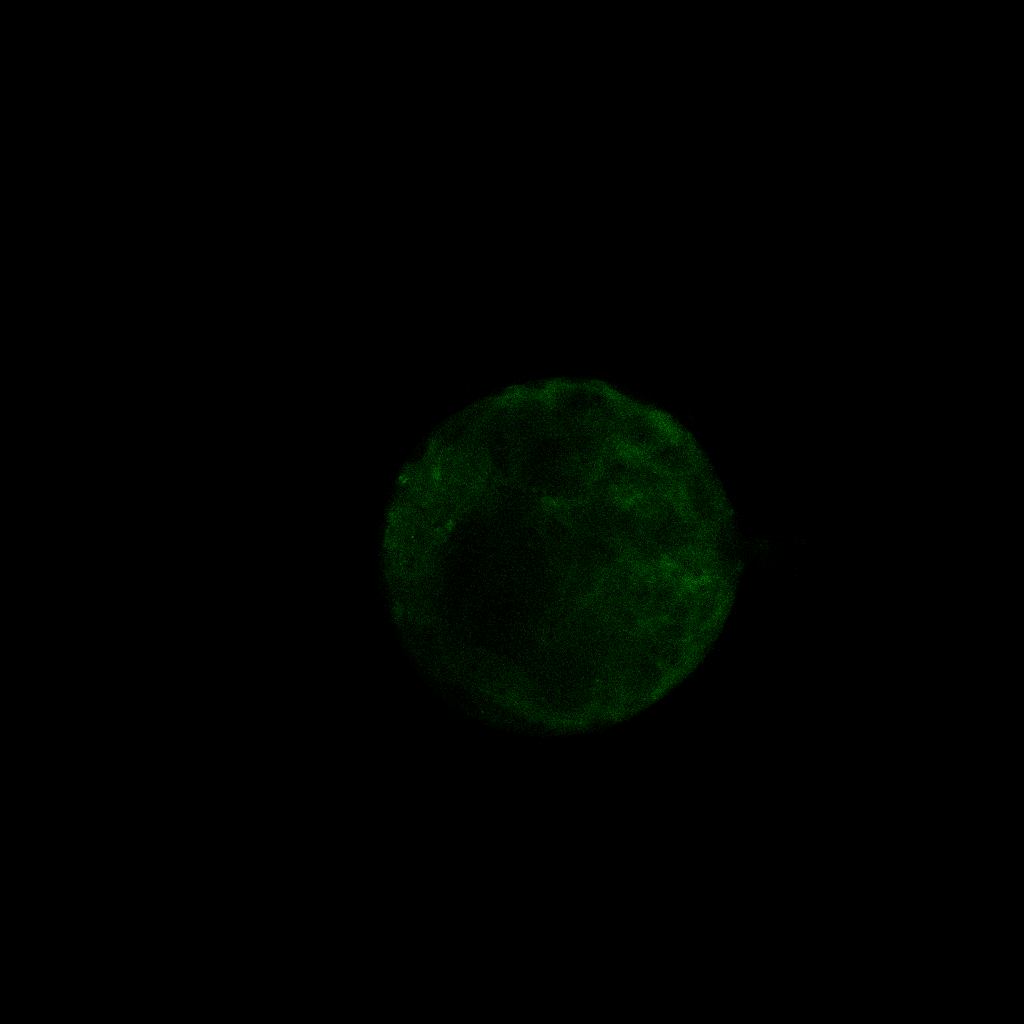

Supplement: Supplementary file 5 [file DataSheet6.ZIP › Raw data for figure 6/Control ATP1a1 x 200.tif]

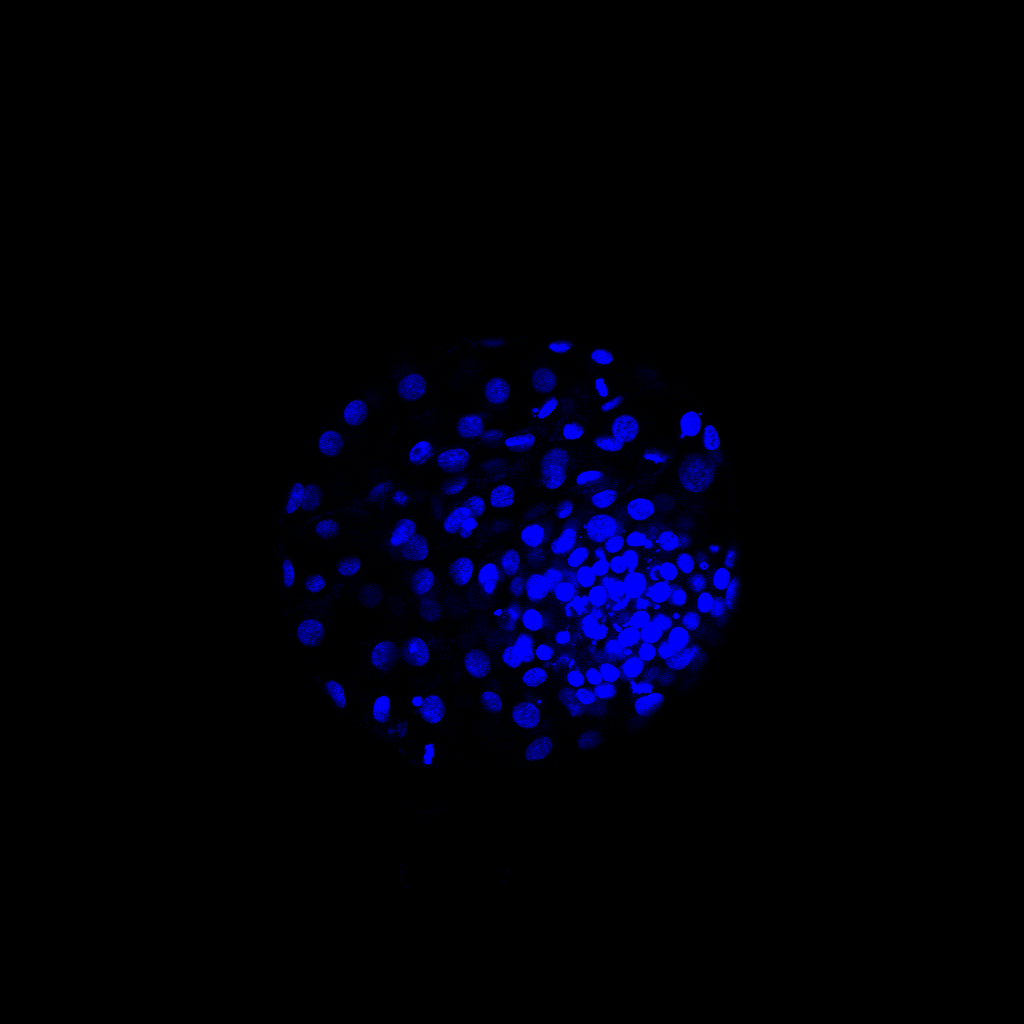

Supplement: Supplementary file 5 [file DataSheet6.ZIP › Raw data for figure 6/GDF-8 AQP3 DAPI x 200.tif]

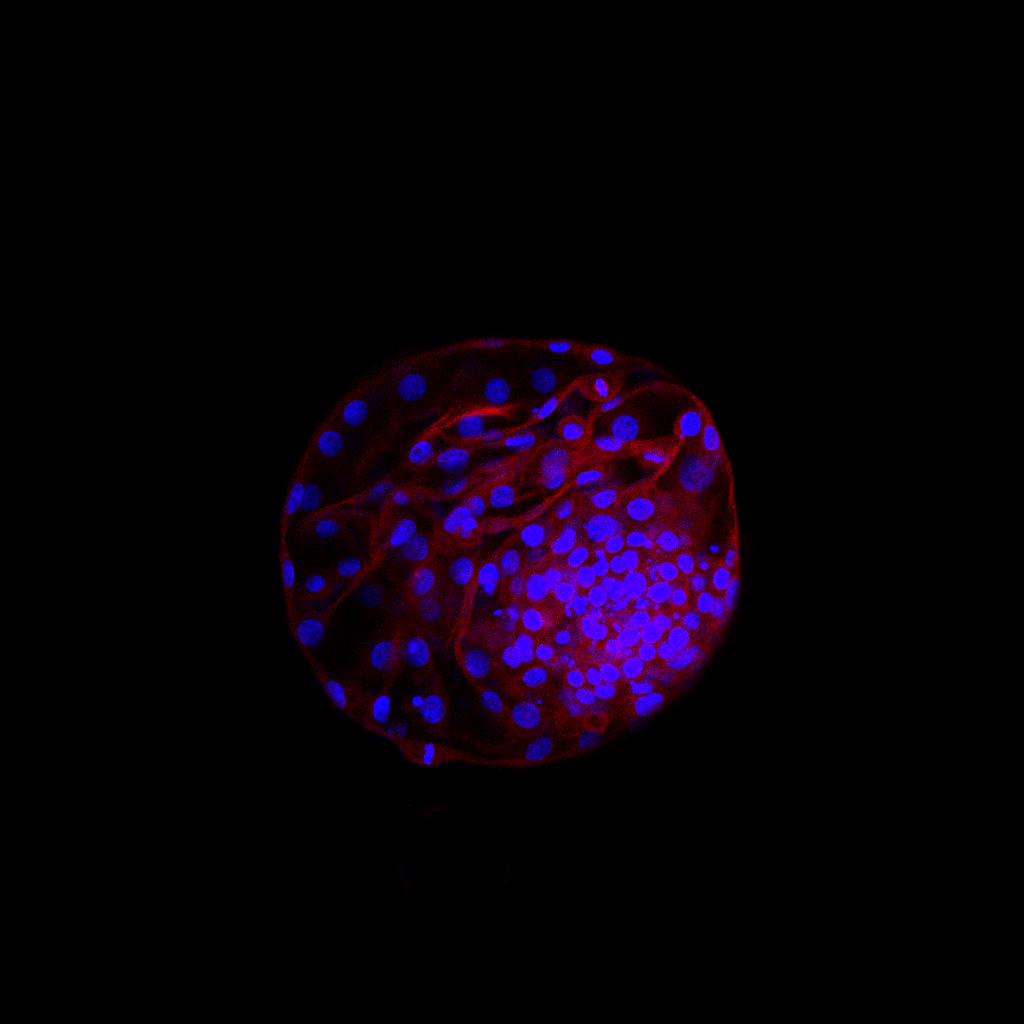

Supplement: Supplementary file 5 [file DataSheet6.ZIP › Raw data for figure 6/GDF-8 AQP3 Merge x 200.tif]

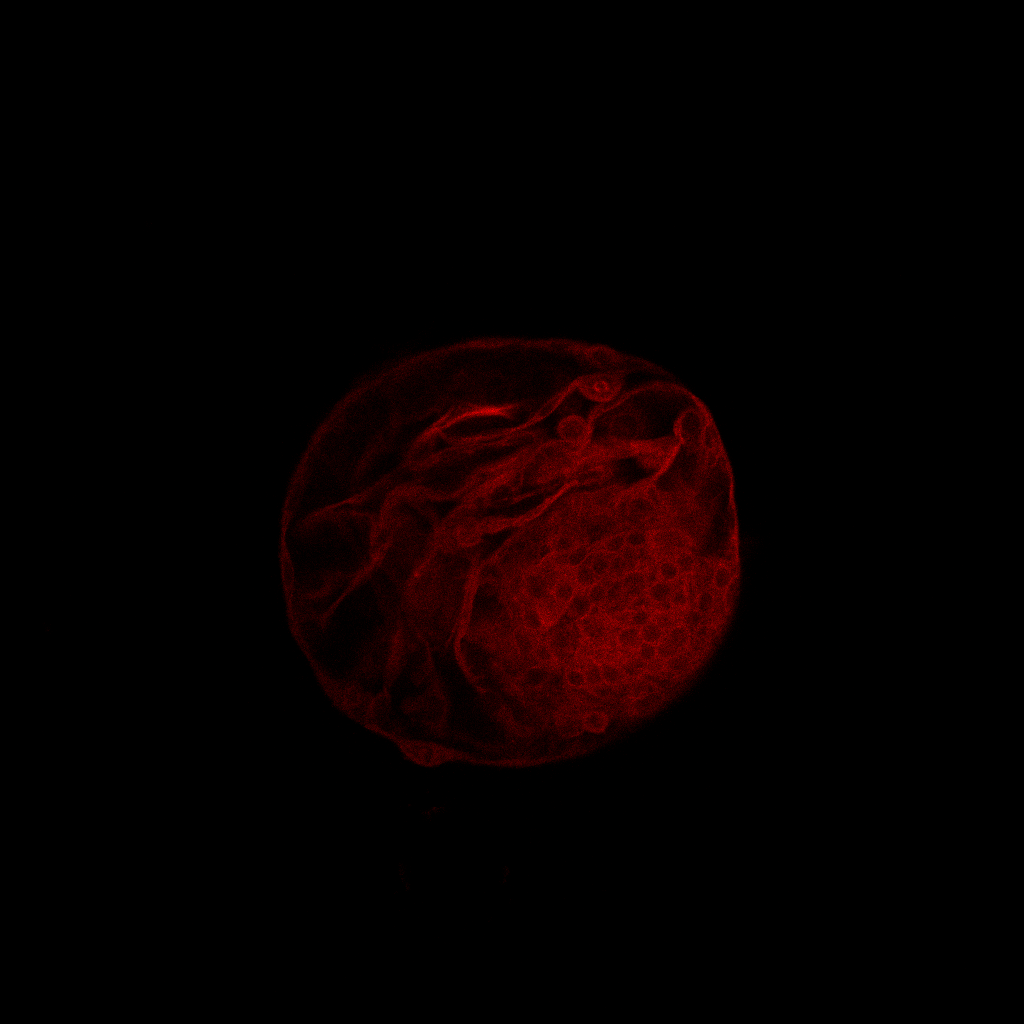

Supplement: Supplementary file 5 [file DataSheet6.ZIP › Raw data for figure 6/GDF-8 AQP3 x 200.tif]

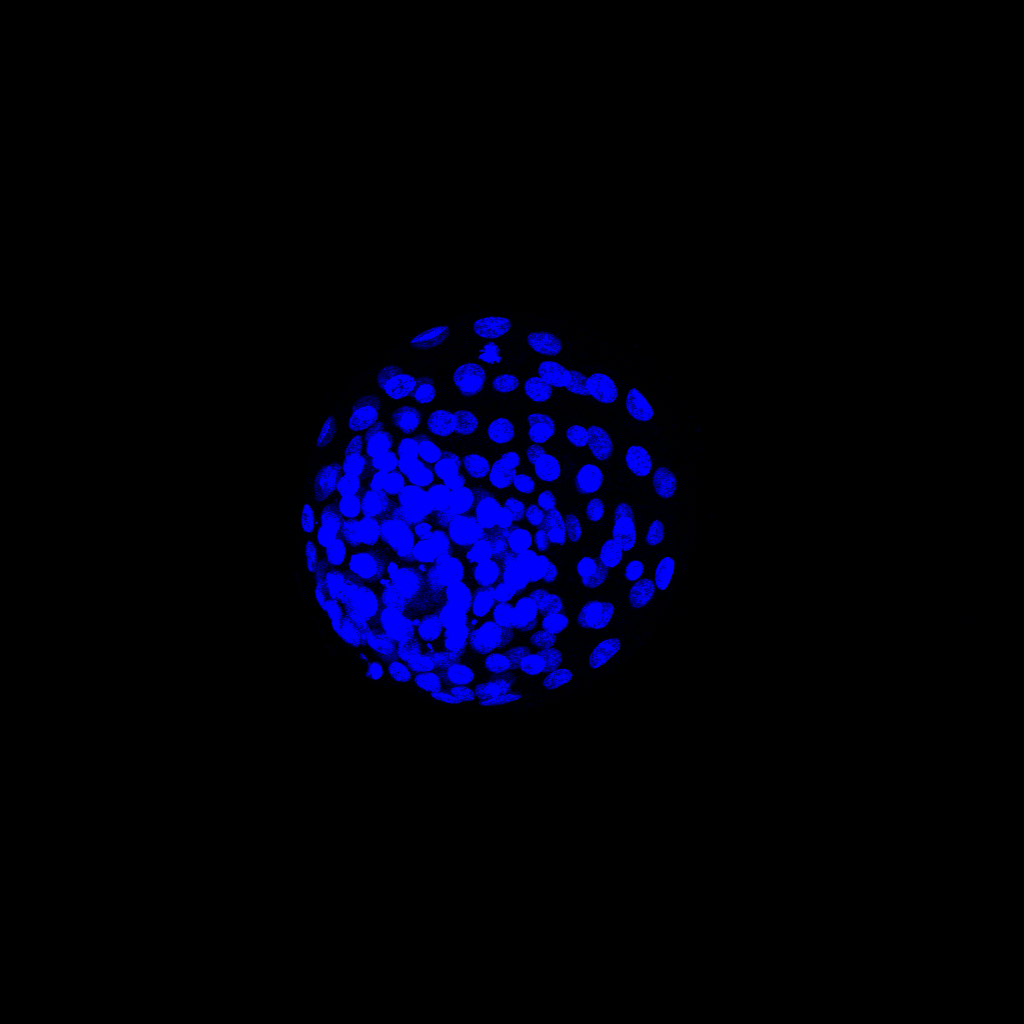

Supplement: Supplementary file 5 [file DataSheet6.ZIP › Raw data for figure 6/GDF-8 ATP1a1 DAPI x 200.tif]

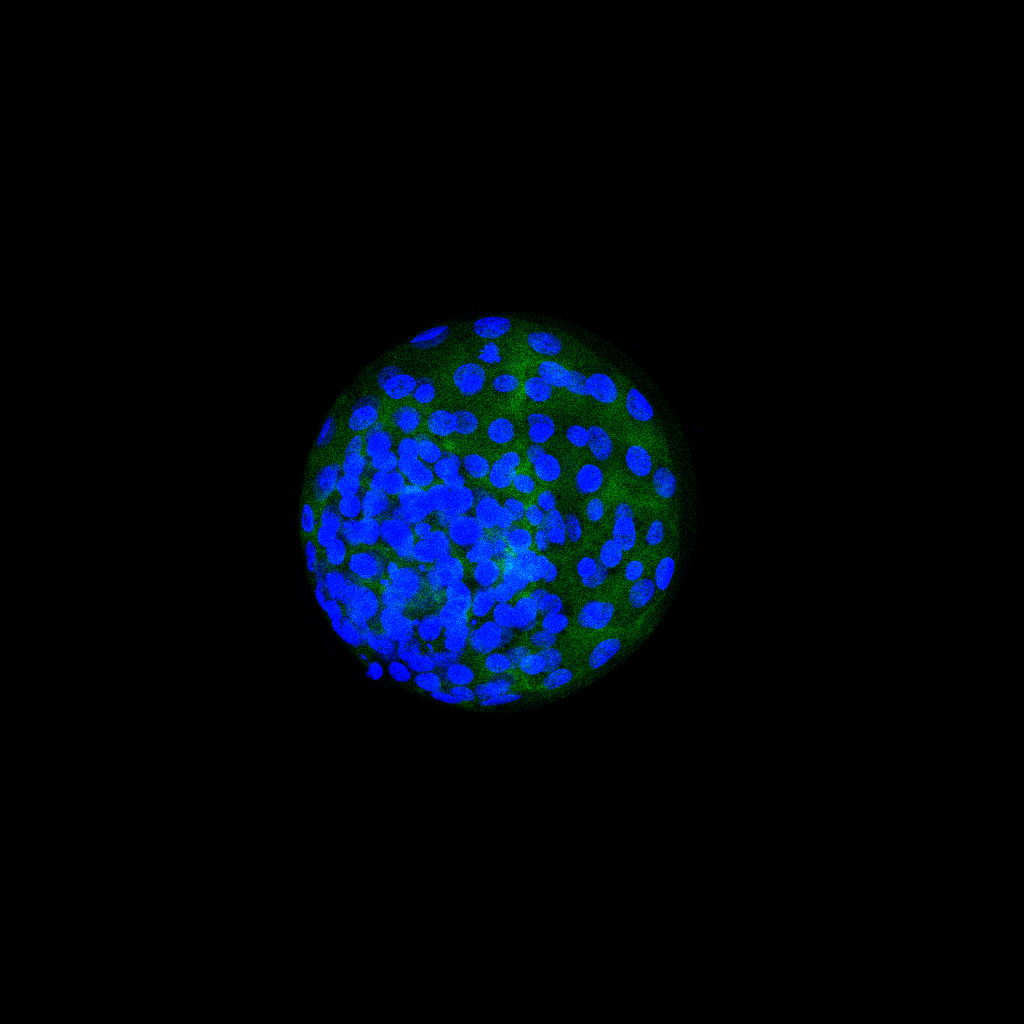

Supplement: Supplementary file 5 [file DataSheet6.ZIP › Raw data for figure 6/GDF-8 ATP1a1 Merge x 200.tif]

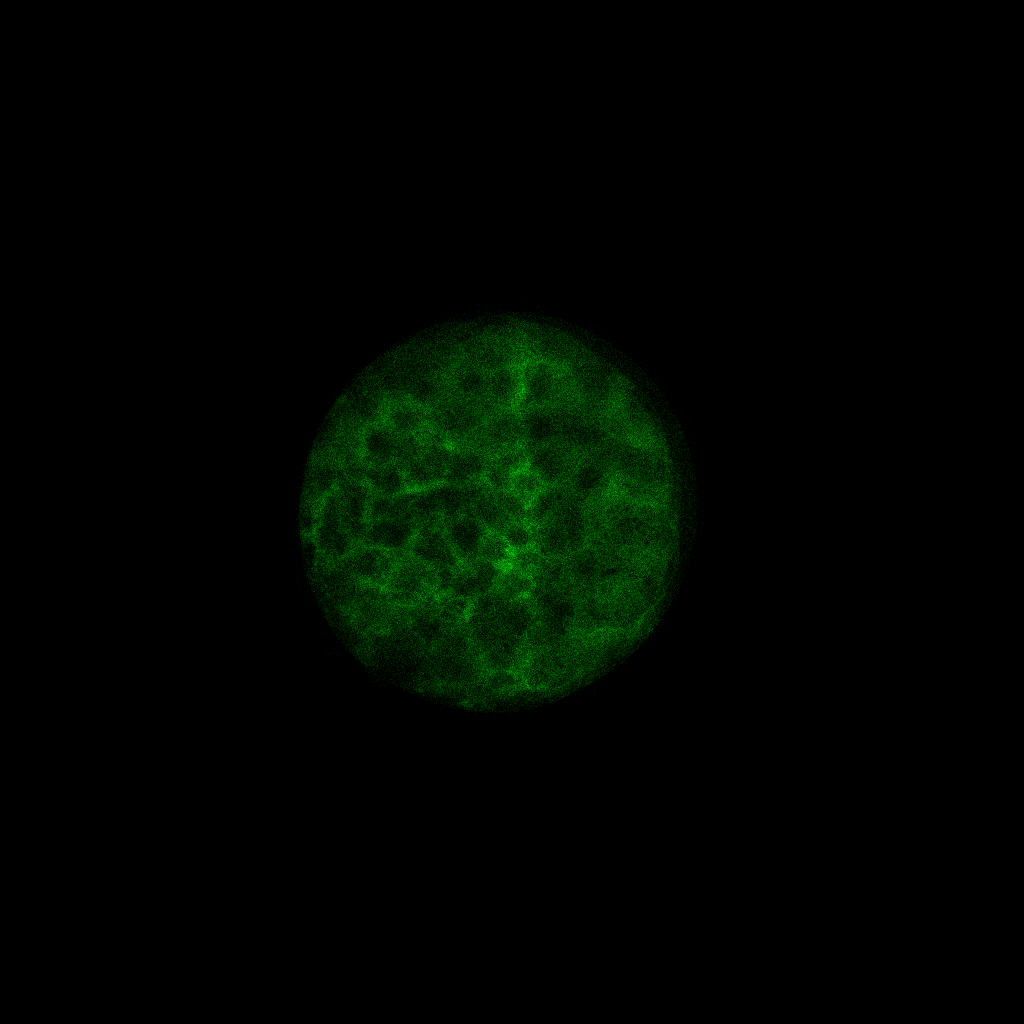

Supplement: Supplementary file 5 [file DataSheet6.ZIP › Raw data for figure 6/GDF-8 ATP1a1 x 200.tif]

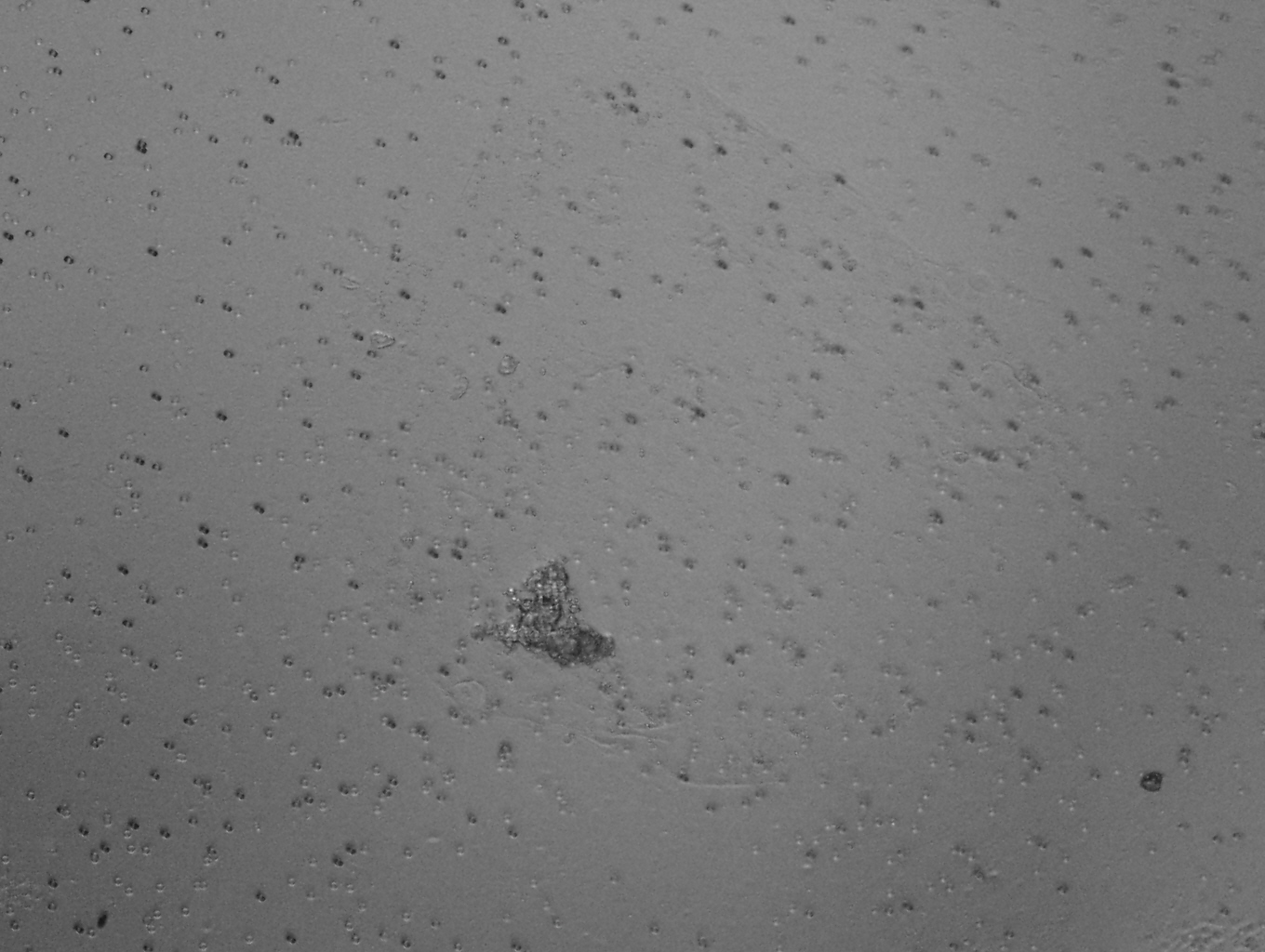

Supplement: Supplementary file 6 [file DataSheet2.ZIP › Raw data for figure 2/Control B.F x 40.jpg]

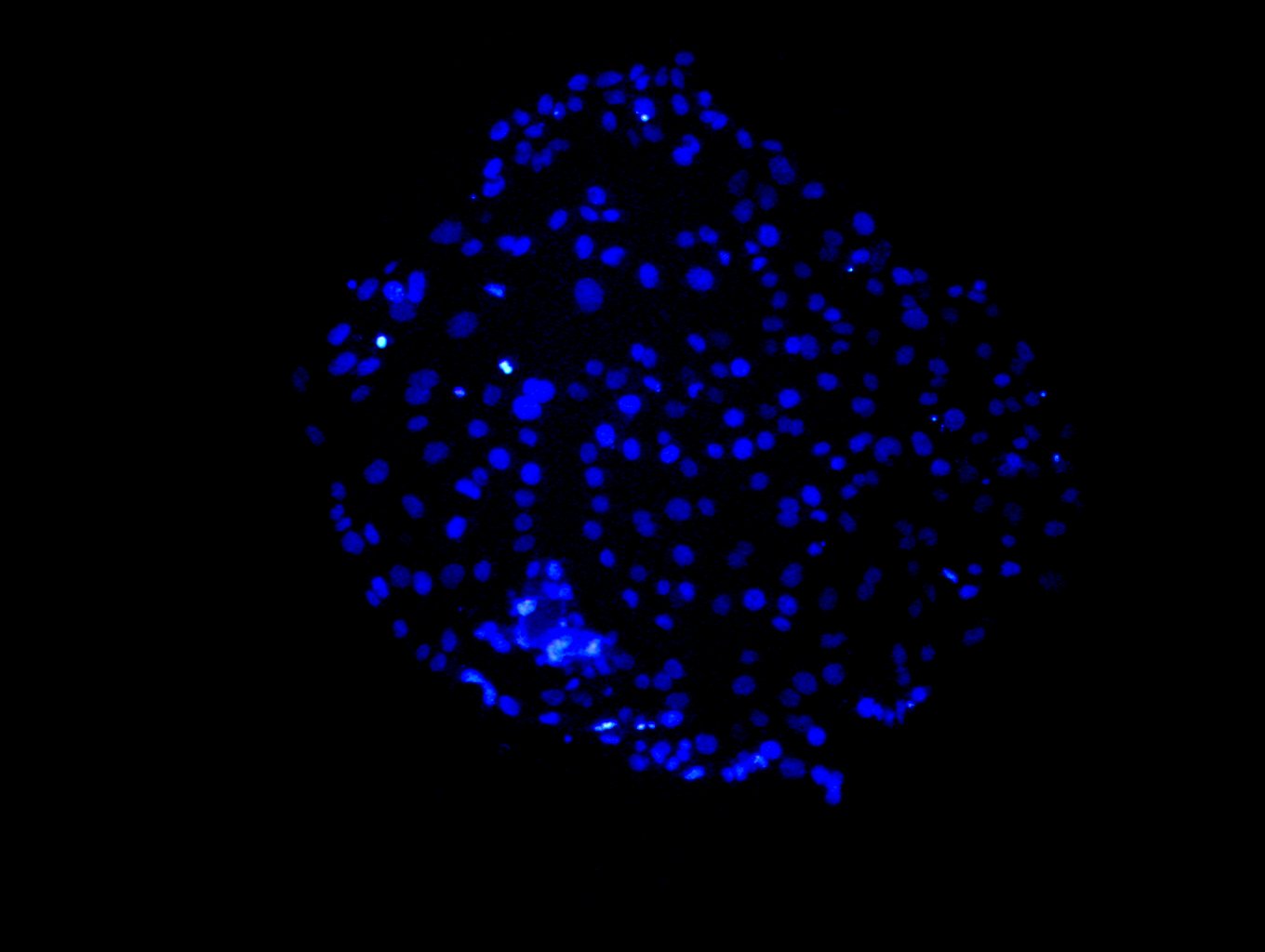

Supplement: Supplementary file 6 [file DataSheet2.ZIP › Raw data for figure 2/Control invasion DAPI x 40.jpg]

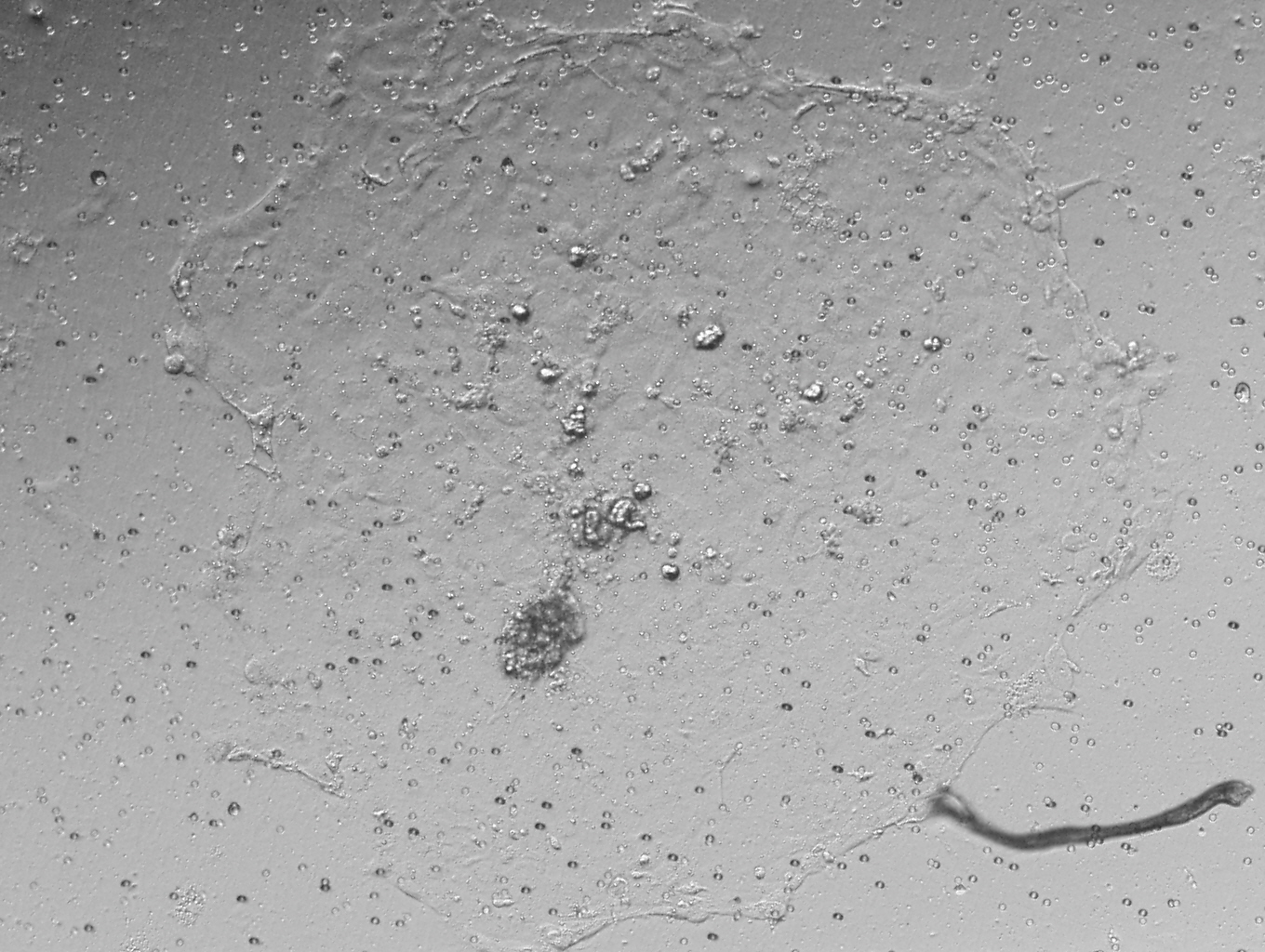

Supplement: Supplementary file 6 [file DataSheet2.ZIP › Raw data for figure 2/GDF-8 B.F x 40.jpg]

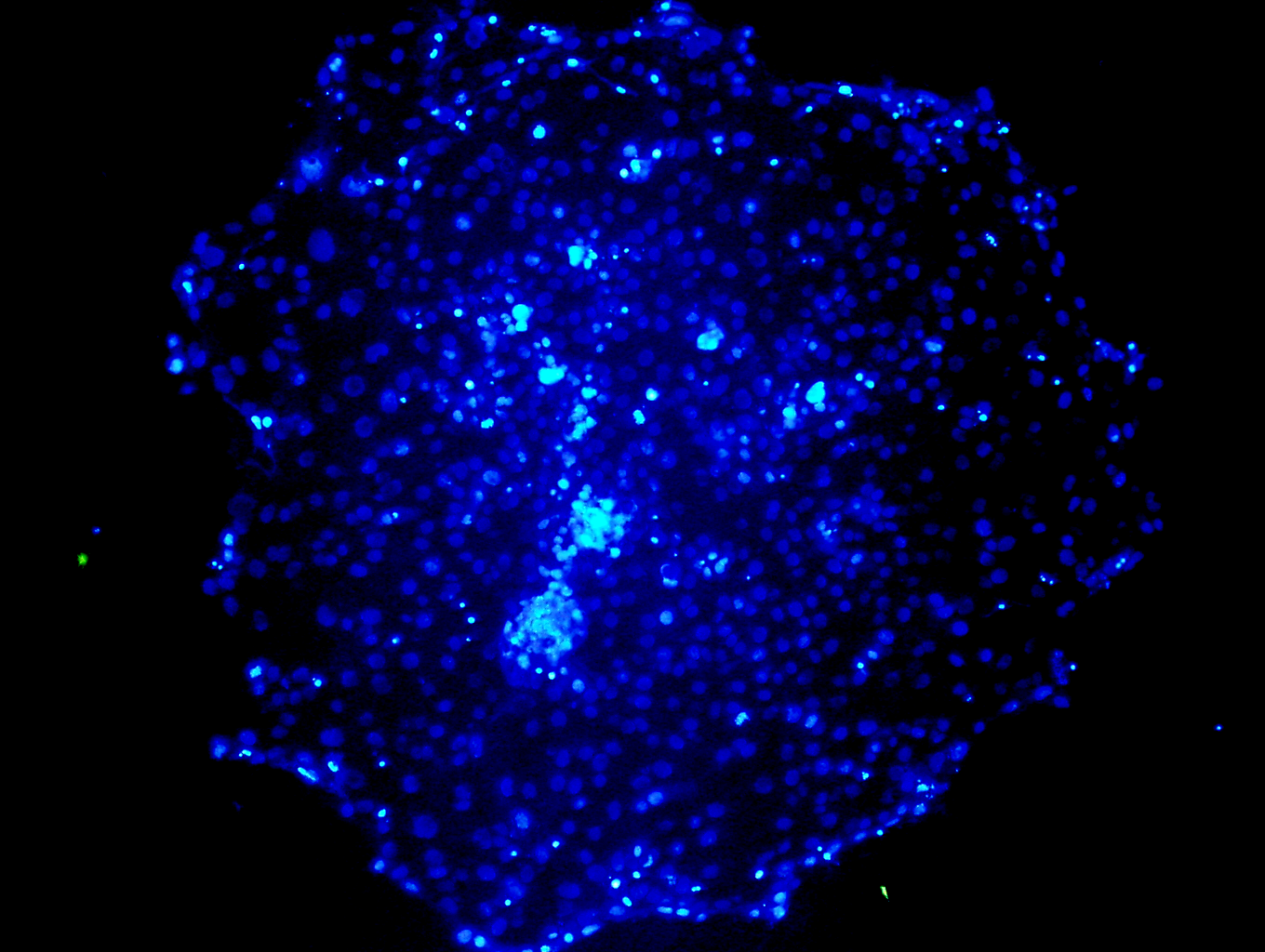

Supplement: Supplementary file 6 [file DataSheet2.ZIP › Raw data for figure 2/GDF-8 invasion DAPI x 40.tif]

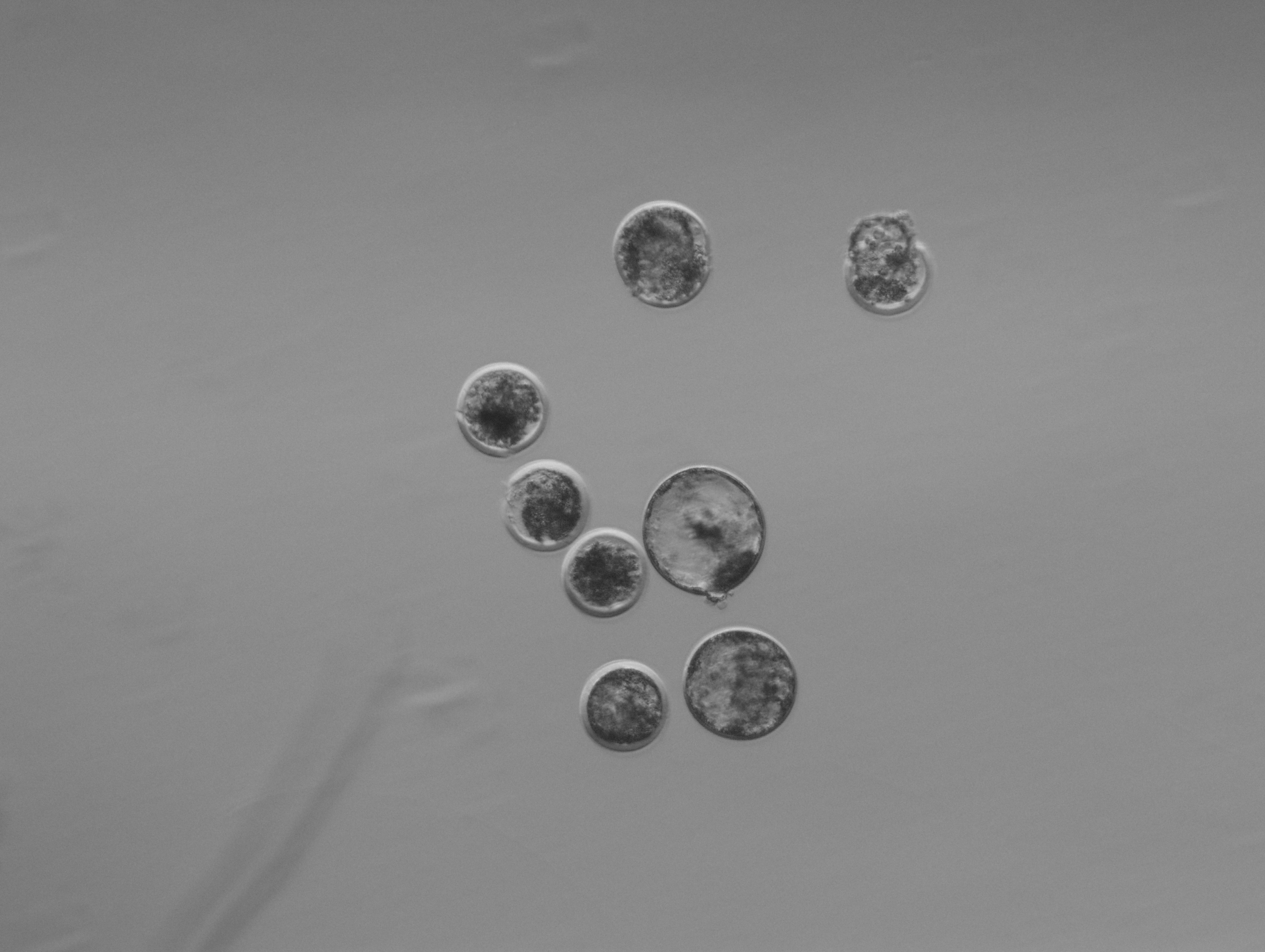

Supplement: Supplementary file 8 [file DataSheet5.ZIP › Raw data for figure 5/Control Hatching 24h x 64.jpg]

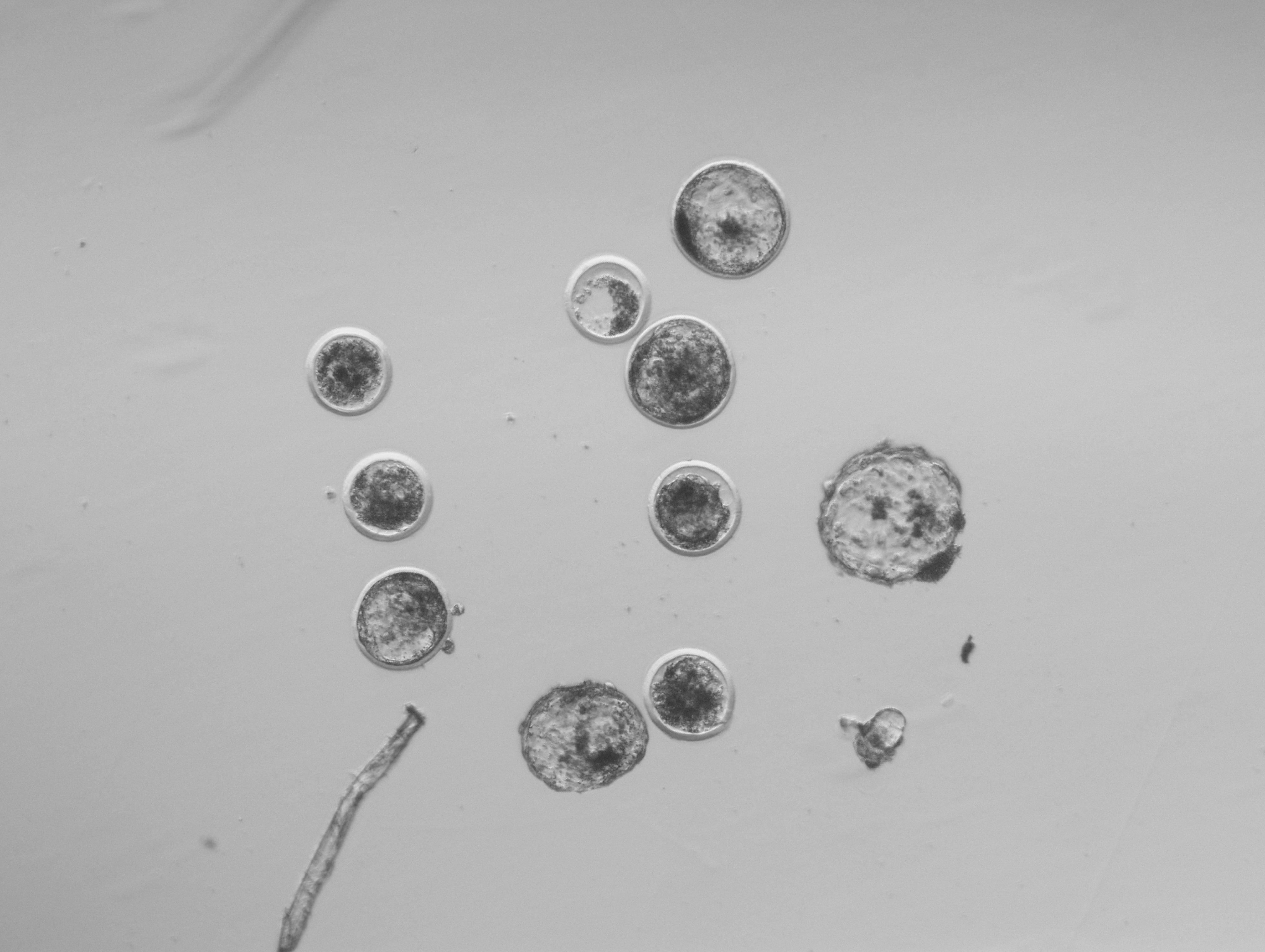

Supplement: Supplementary file 8 [file DataSheet5.ZIP › Raw data for figure 5/Control Hatching 48h x 64.jpg]

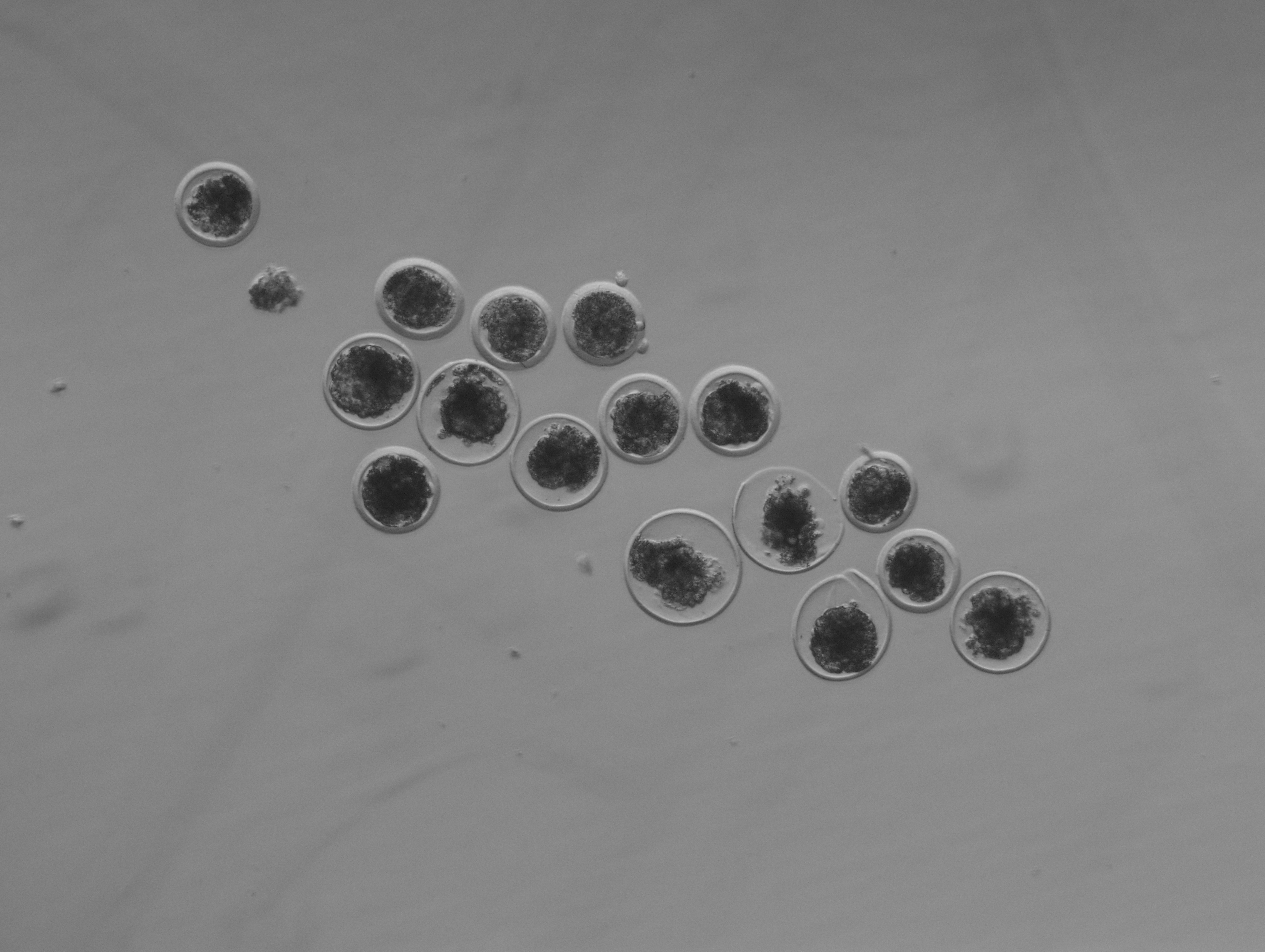

Supplement: Supplementary file 8 [file DataSheet5.ZIP › Raw data for figure 5/Control survival 0h x 64.jpg]

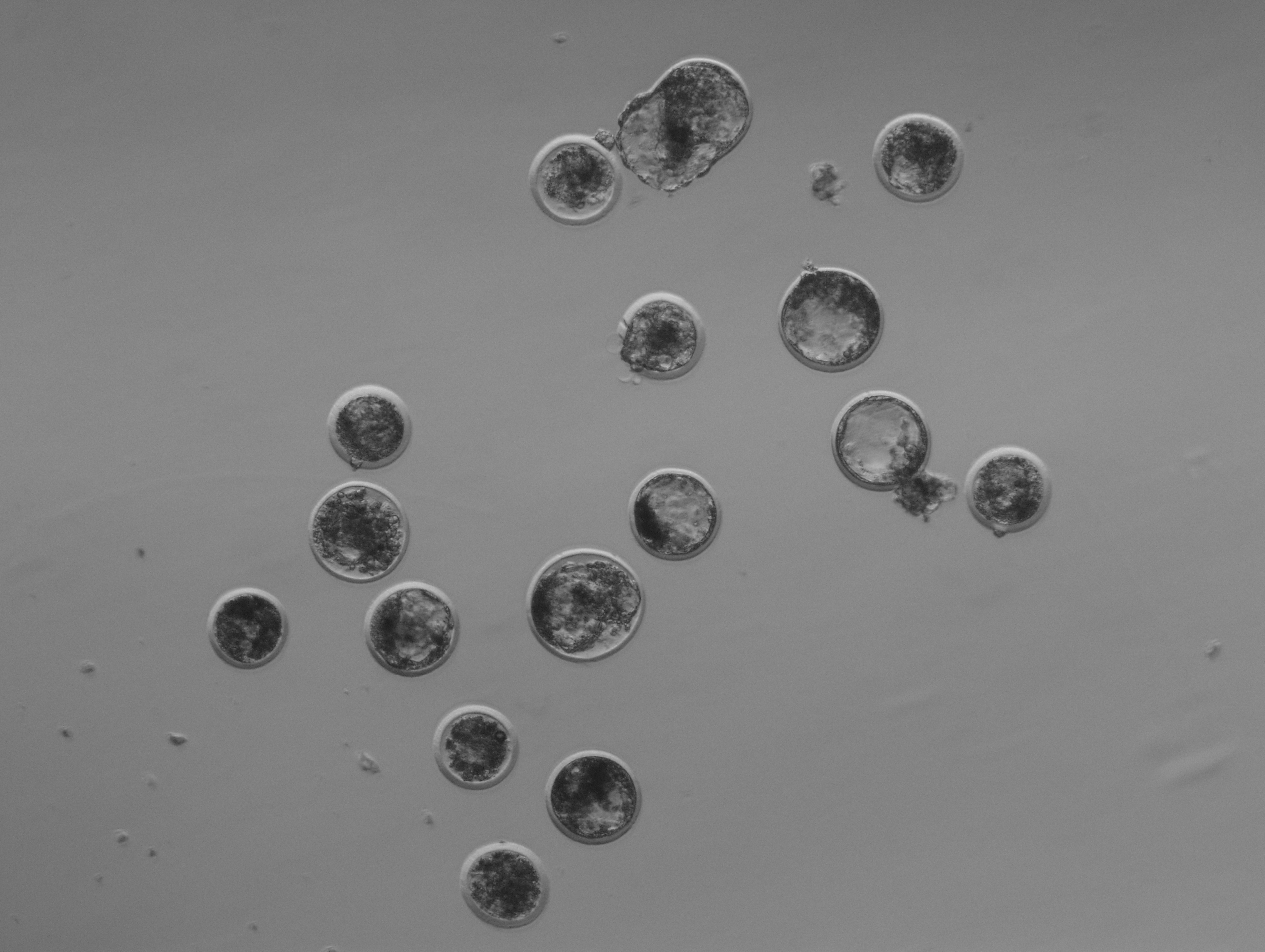

Supplement: Supplementary file 8 [file DataSheet5.ZIP › Raw data for figure 5/Control survival 24h x 64.jpg]

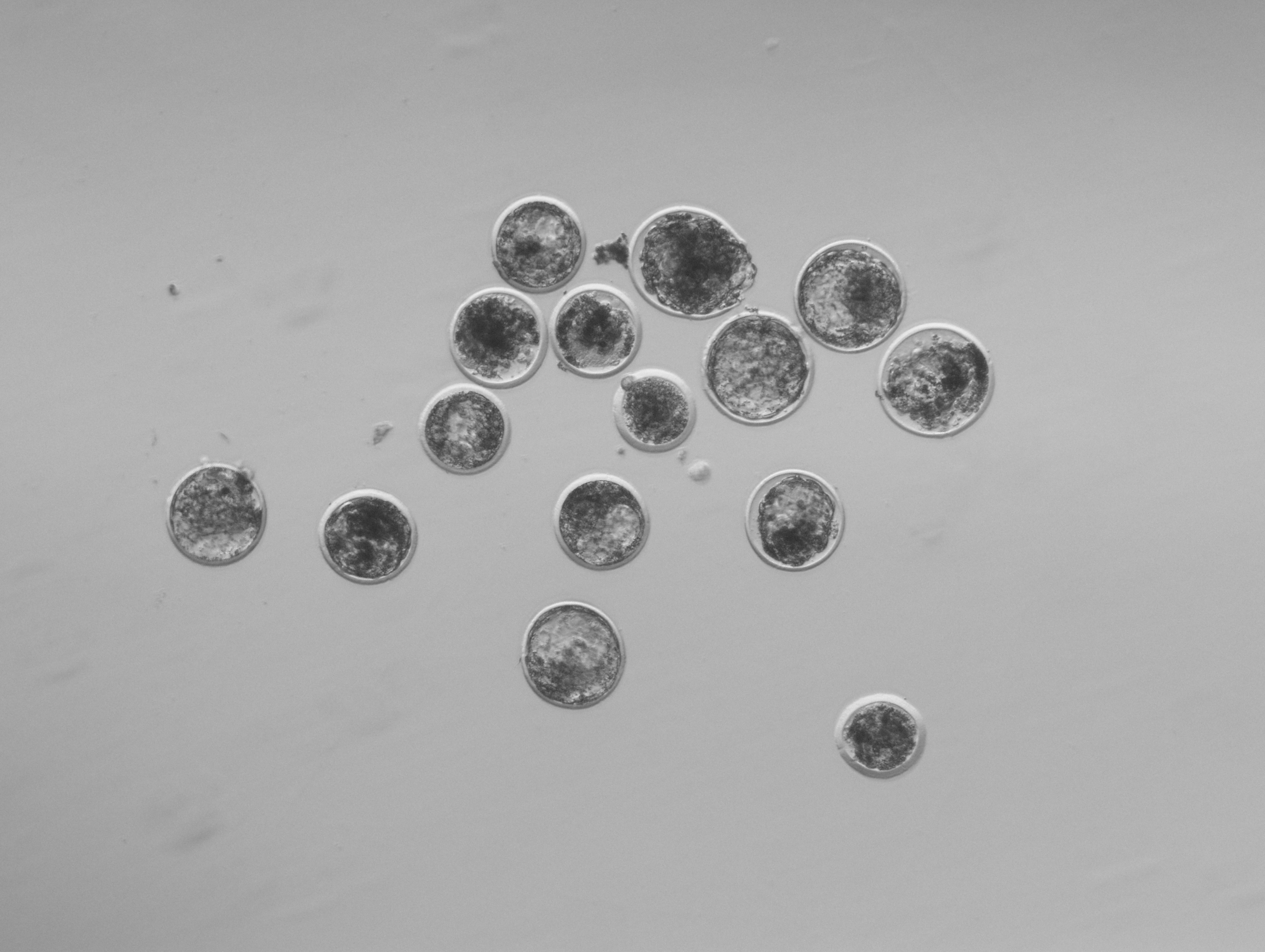

Supplement: Supplementary file 8 [file DataSheet5.ZIP › Raw data for figure 5/Control survival 48h x 64.jpg]

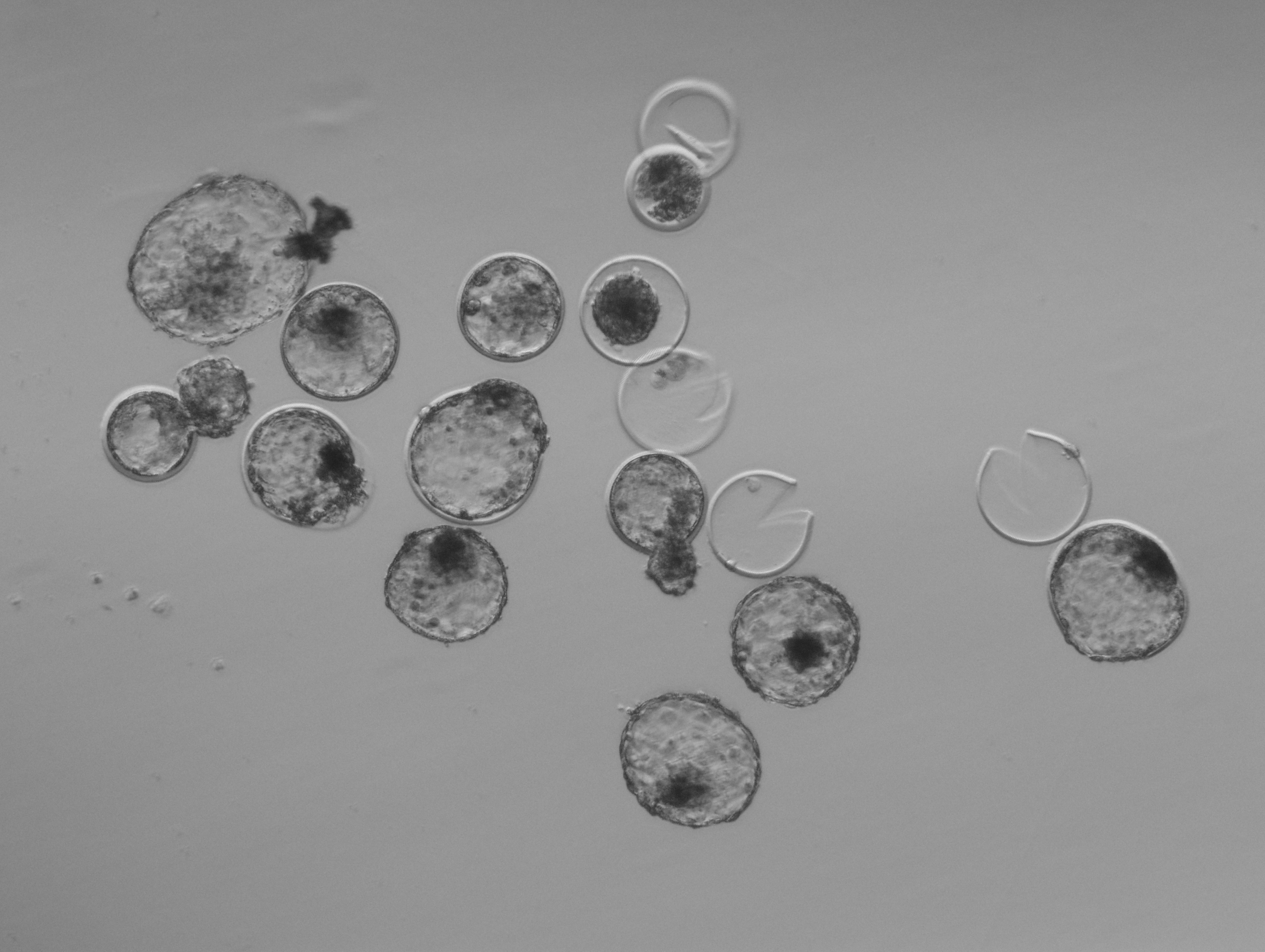

Supplement: Supplementary file 8 [file DataSheet5.ZIP › Raw data for figure 5/GDF-8 Hatching 24h x 64.jpg]

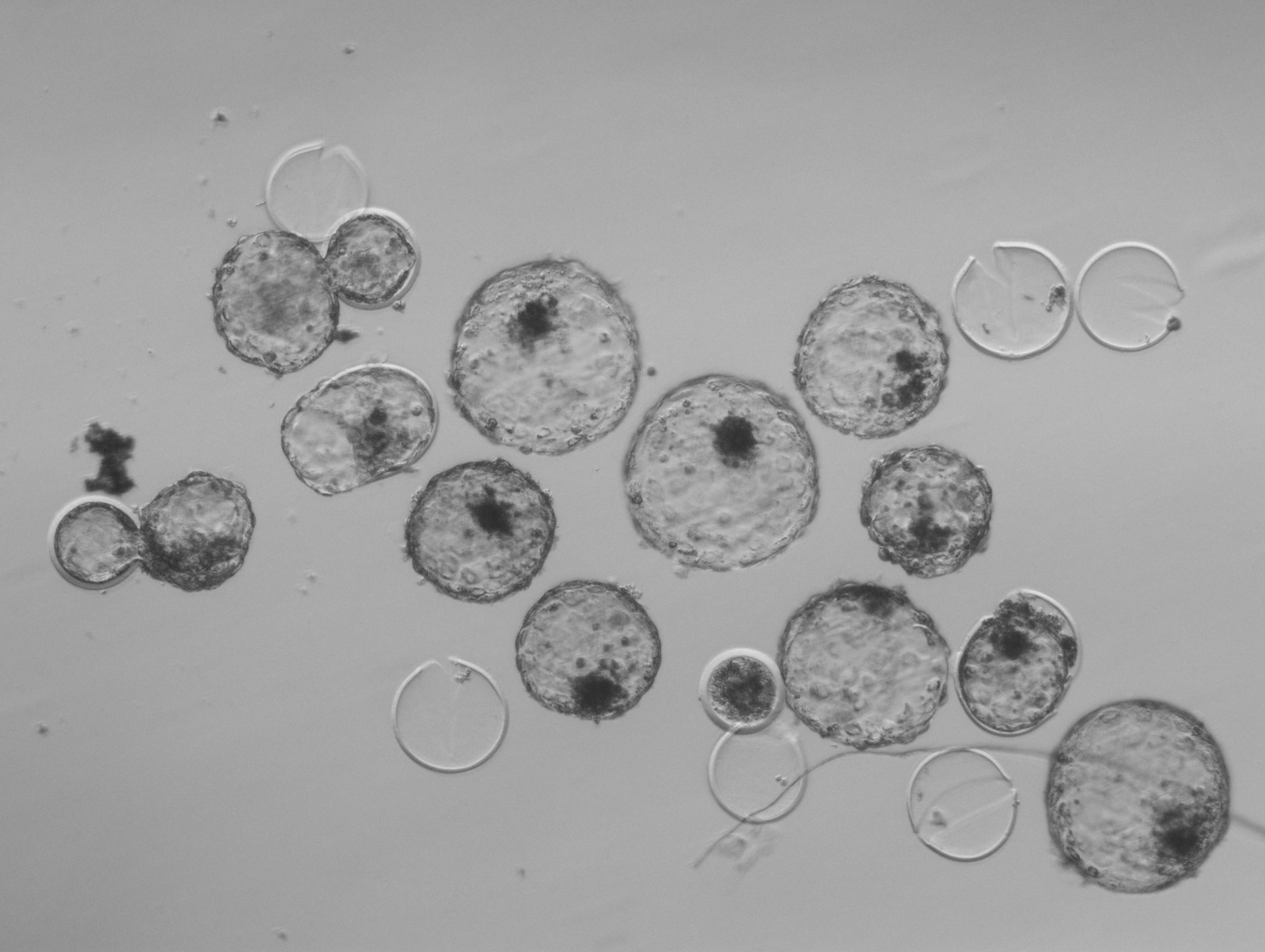

Supplement: Supplementary file 8 [file DataSheet5.ZIP › Raw data for figure 5/GDF-8 Hatching 48h x 64.jpg]

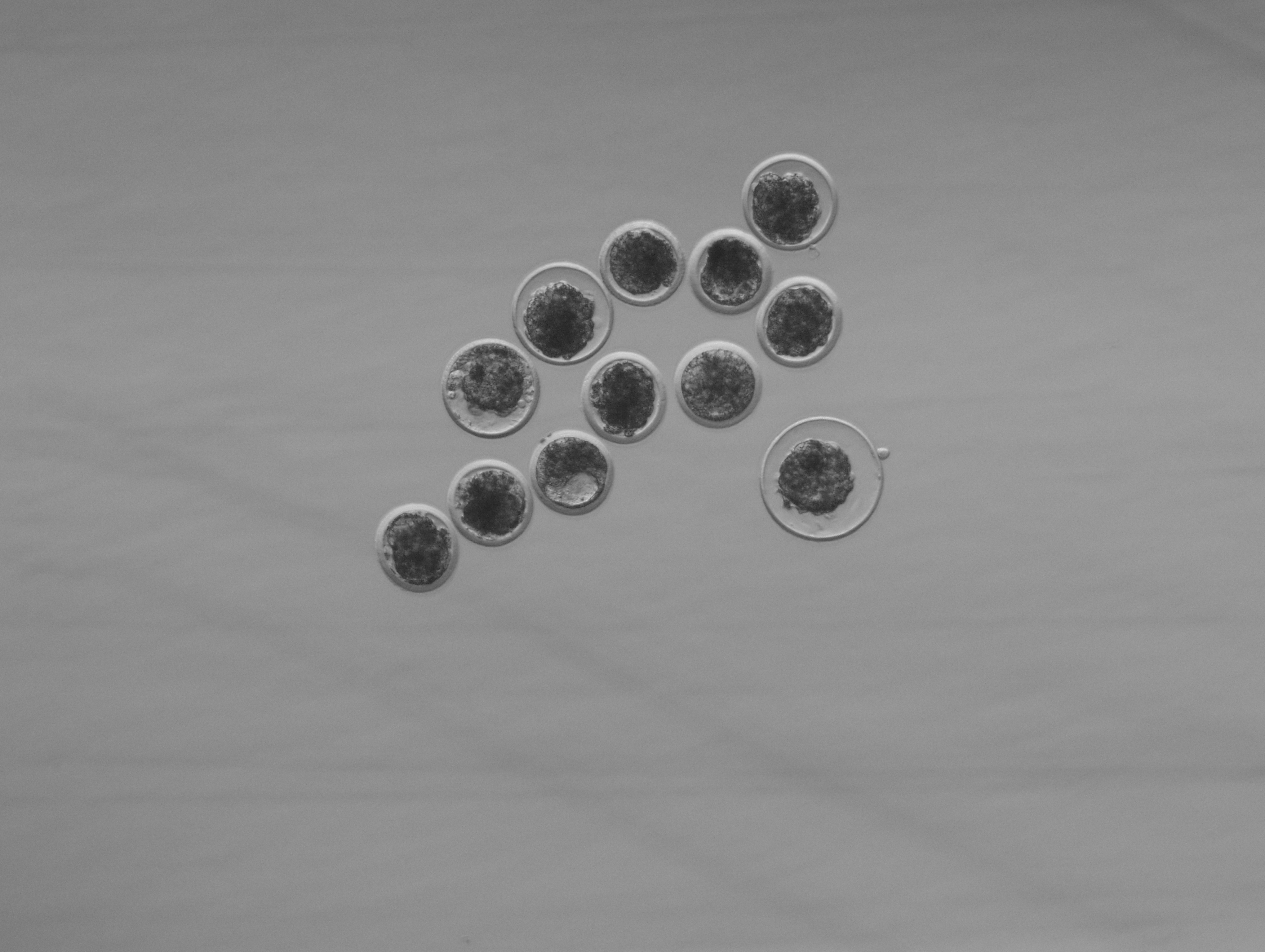

Supplement: Supplementary file 8 [file DataSheet5.ZIP › Raw data for figure 5/GDF-8 survival 0h x 64.jpg]

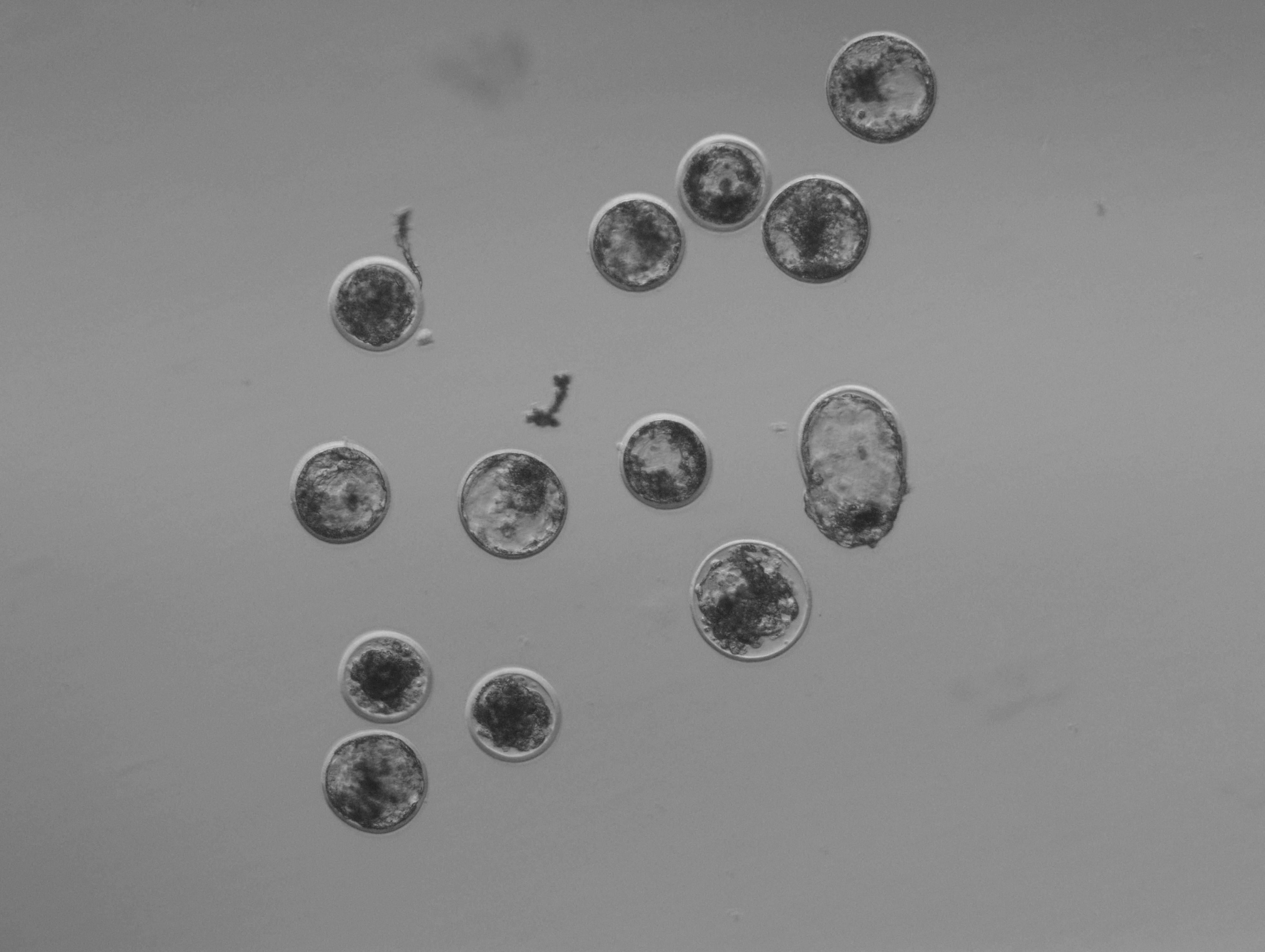

Supplement: Supplementary file 8 [file DataSheet5.ZIP › Raw data for figure 5/GDF-8 survival 24h x 64.jpg]

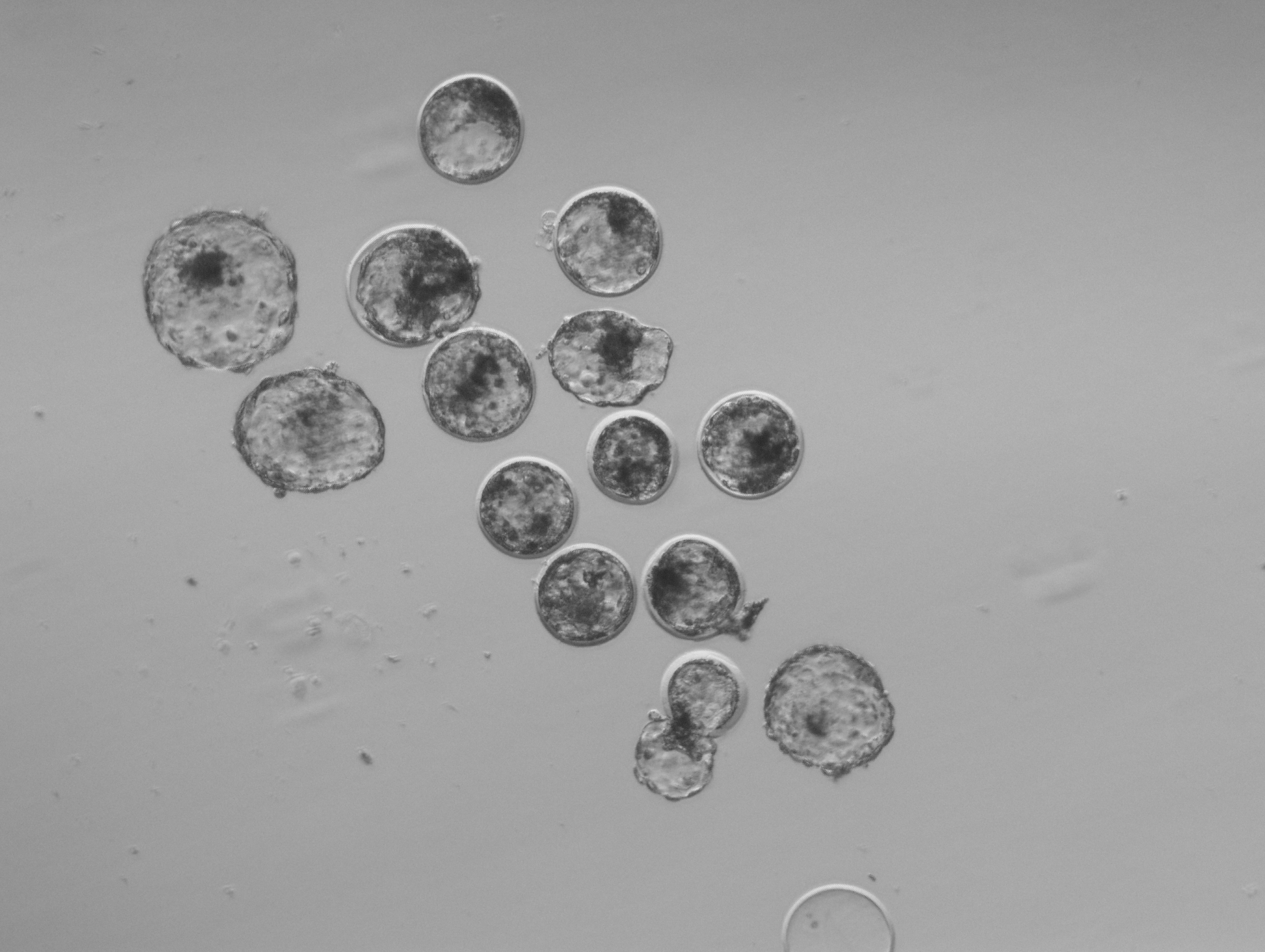

Supplement: Supplementary file 8 [file DataSheet5.ZIP › Raw data for figure 5/GDF-8 survival 48h x 64.jpg]

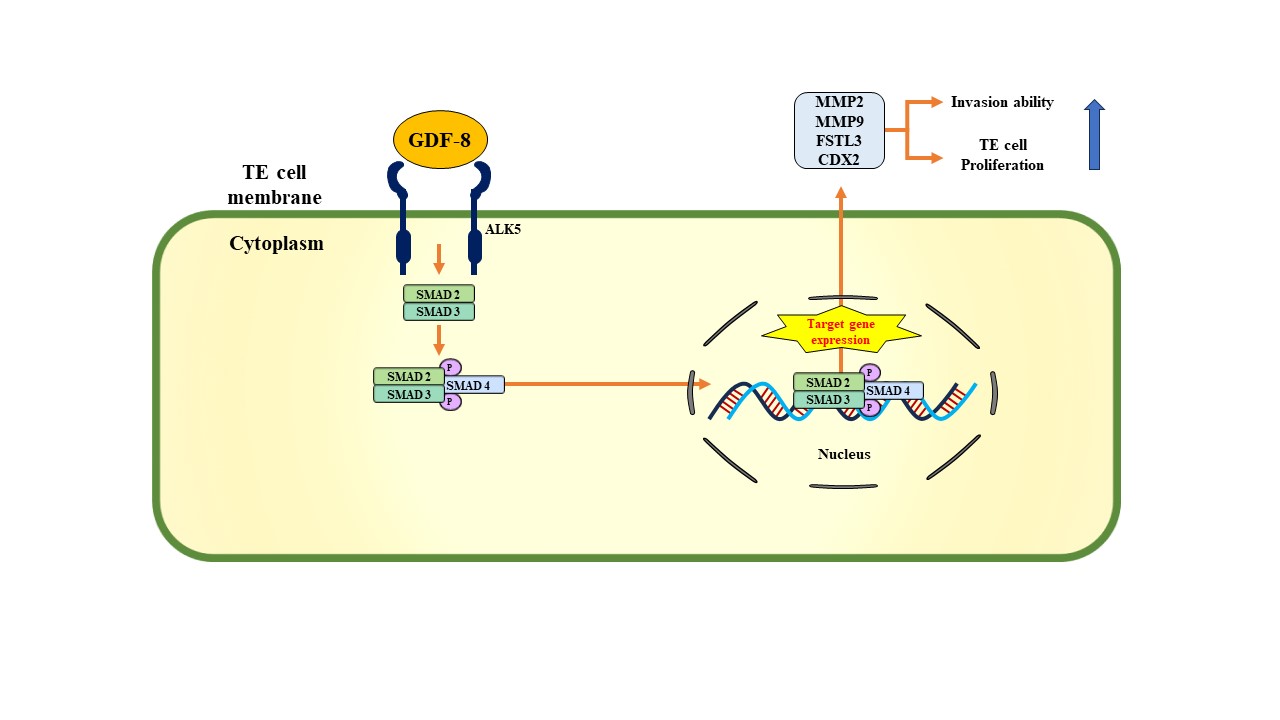

Supplement: Supplementary file 9 [file DataSheet7.ZIP › supplementation figure1&2/Supplementation figure 1.JPG]

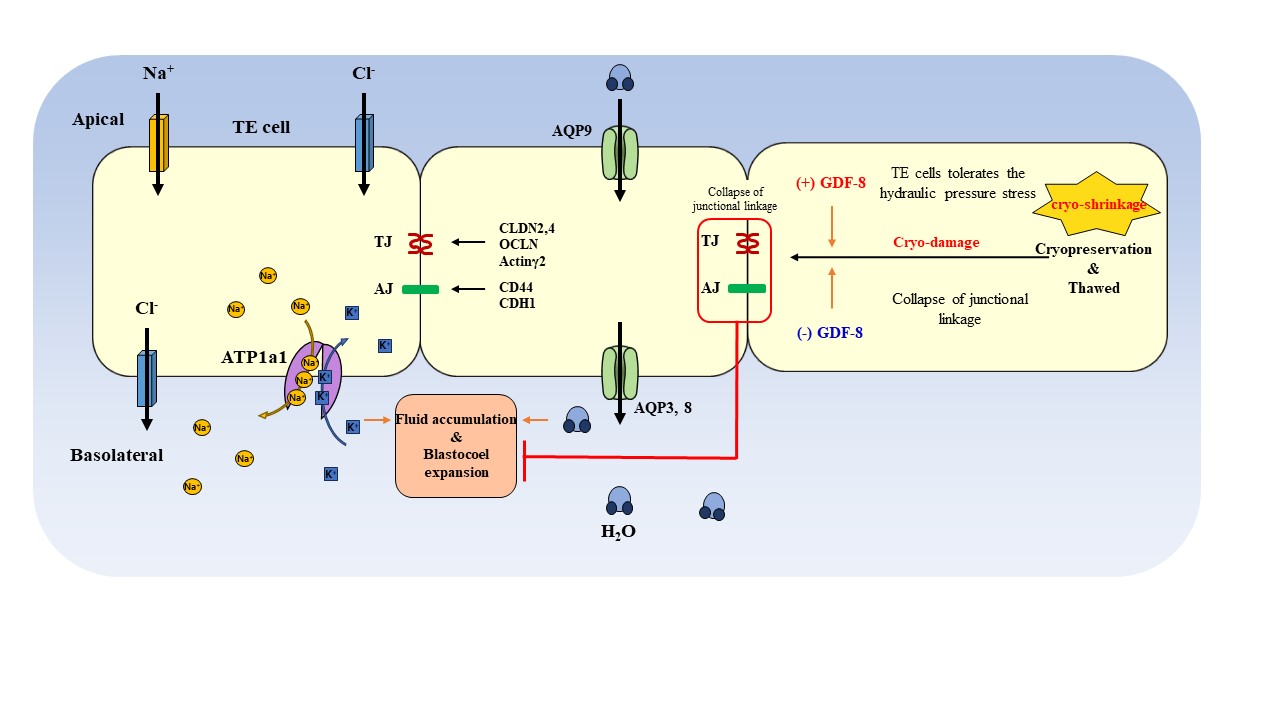

Supplement: Supplementary file 9 [file DataSheet7.ZIP › supplementation figure1&2/Supplementation figure 2.JPG]
